# The SANAD II study of the effectiveness and cost-effectiveness of valproate versus levetiracetam for newly diagnosed generalised and unclassifiable epilepsy: an open-label, non-inferiority, multicentre, phase 4, randomised controlled trial

**DOI:** 10.1016/S0140-6736(21)00246-4

**Published:** 2021-04-10

**Authors:** Anthony Marson, Girvan Burnside, Richard Appleton, Dave Smith, John Paul Leach, Graeme Sills, Catrin Tudur-Smith, Catrin Plumpton, Dyfrig A Hughes, Paula Williamson, Gus A Baker, Silviya Balabanova, Claire Taylor, Richard Brown, Dan Hindley, Stephen Howell, Melissa Maguire, Rajiv Mohanraj, Philip E Smith, SANAD II collaborators, Karen Lanyon, Mark Manford, Manali Chitre, Alasdair Parker, Nina Swiderska, Richard Appleton, James Pauling, Adrian Hughes, Rajat Gupta, Sadia Hanif, Mostafa Awadh, Sharmini Ragunathan, Nicola Cable, Paul Cooper, Daniel Hindley, Karl Rakshi, Sophie Molloy, Markus Reuber, Kunle Ayonrinde, Martin Wilson, Satyanarayana Saladi, John Gibb, Lesley-Ann Funston, Damhait Cassidy, Jonathan Boyd, Mal Ratnayaka, Hani Faza, Martin Sadler, Hassan Al-Moasseb, Clare Galtrey, Damien Wren, Anas Olabi, Geraint Fuller, Muhammed Khan, Chetana Kallappa, Ravi Chinthapalli, Baba Aji, Rhys Davies, Kathryn Foster, Nikolas Hitiris, Melissa Maguire, Nahin Hussain, Simon Dowson, Julie Ellison, Basil Sharrack, Vandna Gandhi, Rob Powell, Phil Tittensor, Beatrice Summers, Sastry Shashikiran, Penelope J Dison, Shanika Samarasekera, Doug McCorry, Kathleen White, Kannan Nithi, Martin Richardson, Richard Brown, Rupert Page, David Deekollu, Sean Slaght, Stephen Warriner, Mansoor Ahmed, Abhijit Chaudhuri, Gabriel Chow, Javier Artal, Danute Kucinskiene, Harish Sreenivasa, Singara Velmurugan,, Christos S Zipitis, Brendan McLean, Vaithianathar Lal, Angelous Gregoriou, Paul Maddison, Trevor Pickersgill, Joseph Anderson, Charlotte Lawthom, Stephen Howell, Gabriel Whitlingum, Wojtek Rakowicz, Lucy Kinton, Alisa McLellan, Nitish Vora, Sameer Zuberi, Andrew Kelso, Imelda Hughes, John Martland, Hedley Emsley, Christian de Goede, RP Singh, Carl-Christian Moor, Julia Aram, Rajiv Mohanraj, Kumar Sakthivel, Suresh Nelapatla, Chris Rittey, Ashwin Pinto, John Paul Leach, Hannah Cock, Anna Richardson, Erika Houston, Christopher Cooper, Geoff Lawson, Albert Massarano, Christine Burness, Anthony Marson, Dave Smith, Udo Wieshmann, Indranil Dey, Puthuval Sivakumar, Lap-Kong Yeung, Philip Smith, Hemalata Bentur, Tom Heafield, Anna Mathew, David Smith, Praveen Jauhari

**Affiliations:** a Department of Molecular and Clinical Pharmacology, University of Liverpool, Liverpool, UK; b Department of Health Data Science, University of Liverpool, Liverpool, UK; c Liverpool Clinical Trials Centre, University of Liverpool, Liverpool, UK; d The Roald Dahl EEG Unit, Alder Hey Children's Health Park, Liverpool, UK; e The Walton Centre NHS Foundation Trust, Liverpool, UK; f School of Medicine, University of Glasgow, Glasgow, UK; g Centre for Health Economics and Medicines Evaluation, Bangor University, Bangor, Wales, UK; h Addenbrooke's Hospital NHS Foundation Trust, Cambridge, UK; i Bolton NHS Foundation Trust, Royal Bolton Hospital, Lancashire, UK; j Department of Neurology, Royal Hallamshire Hospital, Sheffield, UK; k School of Medicine, University of Leeds, Leeds, UK; l Salford Royal NHS Foundation Trust, Manchester, UK; m The Alan Richens Epilepsy Unit, University Hospital of Wales, Cardiff, Wales, UK

## Abstract

**Background:**

Valproate is a first-line treatment for patients with newly diagnosed idiopathic generalised or difficult to classify epilepsy, but not for women of child-bearing potential because of teratogenicity. Levetiracetam is increasingly prescribed for these patient populations despite scarcity of evidence of clinical effectiveness or cost-effectiveness. We aimed to compare the long-term clinical effectiveness and cost-effectiveness of levetiracetam compared with valproate in participants with newly diagnosed generalised or unclassifiable epilepsy.

**Methods:**

We did an open-label, randomised controlled trial to compare levetiracetam with valproate as first-line treatment for patients with generalised or unclassified epilepsy. Adult and paediatric neurology services (69 centres overall) across the UK recruited participants aged 5 years or older (with no upper age limit) with two or more unprovoked generalised or unclassifiable seizures. Participants were randomly allocated (1:1) to receive either levetiracetam or valproate, using a minimisation programme with a random element utilising factors. Participants and investigators were aware of treatment allocation. For participants aged 12 years or older, the initial advised maintenance doses were 500 mg twice per day for levetiracetam and valproate, and for children aged 5–12 years, the initial daily maintenance doses advised were 25 mg/kg for valproate and 40 mg/kg for levetiracetam. All drugs were administered orally. SANAD II was designed to assess the non-inferiority of levetiracetam compared with valproate for the primary outcome time to 12-month remission. The non-inferiority limit was a hazard ratio (HR) of 1·314, which equates to an absolute difference of 10%. A HR greater than 1 indicated that an event was more likely on valproate. All participants were included in the intention-to-treat (ITT) analysis. Per-protocol (PP) analyses excluded participants with major protocol deviations and those who were subsequently diagnosed as not having epilepsy. Safety analyses included all participants who received one dose of any study drug. This trial is registered with the ISRCTN registry, 30294119 (EudraCt number: 2012-001884-64).

**Findings:**

520 participants were recruited between April 30, 2013, and Aug 2, 2016, and followed up for a further 2 years. 260 participants were randomly allocated to receive levetiracetam and 260 participants to receive valproate. The ITT analysis included all participants and the PP analysis included 255 participants randomly allocated to valproate and 254 randomly allocated to levetiracetam. Median age of participants was 13·9 years (range 5·0–94·4), 65% were male and 35% were female, 397 participants had generalised epilepsy, and 123 unclassified epilepsy. Levetiracetam did not meet the criteria for non-inferiority in the ITT analysis of time to 12-month remission (HR 1·19 [95% CI 0·96–1·47]); non-inferiority margin 1·314. The PP analysis showed that the 12-month remission was superior with valproate than with levetiracetam. There were two deaths, one in each group, that were unrelated to trial treatments. Adverse reactions were reported by 96 (37%) participants randomly assigned to valproate and 107 (42%) participants randomly assigned to levetiracetam. Levetiracetam was dominated by valproate in the cost-utility analysis, with a negative incremental net health benefit of −0·040 (95% central range −0·175 to 0·037) and a probability of 0·17 of being cost-effectiveness at a threshold of £20 000 per quality-adjusted life-year. Cost-effectiveness was based on differences between treatment groups in costs and quality-adjusted life-years.

**Interpretation:**

Compared with valproate, levetiracetam was found to be neither clinically effective nor cost-effective. For girls and women of child-bearing potential, these results inform discussions about benefit and harm of avoiding valproate.

**Funding:**

National Institute for Health Research Health Technology Assessment Programme.

**Research in context**

**Evidence before this study**

At the time of the design of this trial (SANAD II), valproate was recommended as a first-line treatment for patients with newly diagnosed generalised epilepsy, which includes syndromes such as absence epilepsies, juvenile myoclonic epilepsy, and generalised epilepsy with tonic-clonic seizures on waking, and for unclassified epilepsy. The evidence base from randomised controlled trials to support this recommendation was scarce, partly because valproate became accepted as a first-line treatment in an era before rigorous trials were undertaken. The first SANAD trial (published in 2007) identified valproate as a clinically effective and cost-effective first-line treatment compared with lamotrigine and topiramate. Prognostic modelling of data from SANAD also found similar treatment responses in the differing epilepsy types. A 14-week double-blind randomised trial in patients with absence epilepsies found valproate and ethosuximide superior to lamotrigine for treatment failure. A Cochrane review identified possible confounding in previous studies due to misclassification of focal epilepsy as generalised epilepsy. That review included an individual participant network meta-analysis, which found no evidence of superiority of valproate over other treatments for seizure control, but valproate was superior to carbamazepine, topiramate, and phenobarbital for treatment failure.

Valproate had also been identified as teratogenic and is associated with around a 10% major malformations rate and with around a third of children exposed to valproate in utero having a significant reduction in IQ. During the conduct of SANAD II, the European Medicines Agency and the Medicines for Healthcare Regulatory Authority implemented a pregnancy prevention programme. Treatment decisions are now particularly difficult for women and girls with generalised epilepsy, which typically starts during childhood and adolescence. The main alternatives to valproate are lamotrigine, which is less effective than valproate, and levetiracetam, which previously had unknown effectiveness compared with valproate as no head-to-head randomised trials had been undertaken.

**Added value of this study**

To the best of our knowledge, this study is the first randomised controlled trial to compare the long-term clinical effectiveness and cost-effectiveness of levetiracetam versus valproate for patients with newly diagnosed generalised or unclassified epilepsy. The study is pragmatic in design and recruited a cohort of participants aged over 5 years from routine UK National Health Service clinics, and the results are relevant to every day clinical practice.

Levetiracetam did not meet our definition of non-inferiority for time to 12-month remission compared with valproate and it was inferior for times to treatment failure, 2-year remission from seizures, and first subsequent seizure. In addition, levetiracetam was not found to be a cost-effective alternative.

**Implications of all the available evidence**

For people with generalised epilepsies, the available evidence identifies valproate as more clinically effective and cost-effective than lamotrigine and levetiracetam. Ethosuximide and valproate have similar efficacy for absence epilepsies but ethosuximide is inefficacious for other seizure types (ie, generalised tonic-clonic seizures and myoclonic seizures). For men, valproate should remain a first-line treatment for generalised epilepsies. For women, there should now be further debate to inform practice and policy about avoiding the most effective treatment to minimise the potential risk of harm in future pregnancies.

## Introduction

Epilepsy is a common condition with a prevalence of 0·5–1% and lifetime incidence of up to 5%.^1^, ^2^ It is also a complex condition with many different causes and several seizure types and syndromes, as defined by the International League Against Epilepsy.^3^, ^4^ Epilepsy is uniquely stigmatising and negatively affects quality of life (QOL), education, and employment prospects.^5^, ^6^

Around a third of people with epilepsy have idiopathic generalised epilepsy, also referred to as genetic generalised epilepsy, which includes several syndromes classified according to seizure types and age of onset, such as childhood absence epilepsy and juvenile myoclonic epilepsy.^4^ Although differing syndromes are recognised, prognostic modelling of data from the first Standard And New Antiepileptic Drug (SANAD I)^7^ study indicates that relative treatment responses are consistent across syndromes. Also, at the time of diagnosis, some people cannot be classified as having either a focal or generalised epilepsy, although for many a syndromic diagnosis can be made, usually following further investigation or as more seizures are witnessed.^8^, ^9^

Valproate is currently recommended as a first-line treatment for generalised and for unclassifiable epilepsy as it has a broad spectrum of action,^10^ although there is little evidence from randomised controlled trials (RCTs) to support this recommendation. Cochrane reviews have not found superiority of valproate over other anti-seizure mediations^11^, ^12^, ^13^ and highlight problems with epilepsy classification and sample size of studies analysed. SANAD I identified valproate as a clinically and cost-effective alternative to either lamotrigine or topiramate,^14^ and a double-blind trial^15^ of 16 week therapy in childhood and juvenile absence epilepsy found valproate and ethosuximide superior to lamotrigine for time to treatment failure.

Levetiracetam has been increasingly used as first-line treatment in both focal and generalised epilepsy, particularly for women of childbearing age with a generalised epilepsy. Although there is evidence from RCTs of efficacy as an add-on treatment for some generalised seizure types,^16^, ^17^ and evidence of tolerability as monotherapy compared with valproate,^18^ there is no evidence from RCTs of the clinical efficacy or cost-effectiveness of levetiracetam when used as monotherapy or as first-line treatment in patients with generalised or unclassifiable epilepsy.

Valproate is not recommended for women of childbearing potential as it is associated with a congenital major malformation rate of around 10%.^15^ Moreover, up to a third of children exposed to valproate in utero have a significant reduction in their IQ^16^ and are at increased risk of autism spectrum disorder.^19^ In 2017, the European Medicines Agency and the UK Medicines and Healthcare Products Regulatory Agency launched a pregnancy prevention programme,^20^ stating that women should not be prescribed valproate unless other treatments are ineffective or not tolerated. For women with an idiopathic generalised epilepsy, the two main alternatives are lamotrigine, which is less effective but safer in pregnancy, and levetiracetam, which has increasing evidence to support its safety in pregnancy,^21^, ^22^ but its effectiveness compared with valproate is unknown.

The aim of SANAD II was to compare the long term clinical effectiveness and cost-effectiveness of levetiracetam compared with valproate in participants with newly diagnosed generalised or unclassifiable epilepsy.

## Methods

### Study design and participants

SANAD-II was a phase 4, multicentre, open-label, randomised controlled trial run in the UK National Health Service (NHS) adult neurology and paediatric services. 85 hospital centres were opened, of which 69 recruited participants to this trial. Participants were eligible for recruitment if they were aged 5 years or older, had a history of at least two unprovoked epileptic seizures requiring treatment, their clinical epilepsy diagnosis was a generalised epilepsy syndrome or was unclassifiable, and had never been treated with an anti-seizure medicine (except for emergency treatment in the 2 week period before enrolment). Exclusion criteria included having provoked or acute symptomatic seizures only, currently taking anti-seizure medicine treatment, and having known progressive neurological disease. Epileptic seizures and syndromes were classified according to International League Against Epilepsy classifications^3^, ^4^ on the basis of seizure semiology and electroencephalogram (EEG) results. Instances in which the precise idiopathic generalised epilepsy syndrome was uncertain (eg, for patients with generalised tonic-clonic seizures and generalised spike and wave changes on their EEG), recruiting clinicians were able to classify such patients as having idiopathic generalised epilepsy not specified. SANAD II was granted ethics approval from the North West-Liverpool East Research Ethics Committee on June 7, 2012. The trial protocol has been previously published.^23^

### Randomisation and masking

After providing consent, participants were randomly allocated (1:1) to receive either levetiracetam or valproate. We used a secure, centrally controlled, 24h web-based facility to implement a minimisation program with a random element utilising factors, which were not made known to reduce the risk of predicting allocation. These factors were centre, sex (ie, male or female), and number of previous seizures (ie, 2, 3–5, 6+), which were not made known. Recruiting clinicians were required to initiate trial treatment within 7 days of randomisation. Participants and investigators were not masked were aware of treatment allocation.

### Procedures

Trial treatments were prescribed as per routine NHS practice and dispensed by hospital and community pharmacies, and clinicians prescribed the formulation they considered most appropriate. The trial protocol provided guidance on initial drug titration and maintenance doses based on routine practice, although clinicians were able to tailor these as appropriate. All medications were taken orally. For participants aged 12 years or older, the initial advised maintenance doses were 500 mg twice per day for both levetiracetam and valproate. For children aged 5–12 years, the initial daily maintenance doses advised were 25 mg/kg for valproate and 40 mg/kg for levetiracetam. Subsequent dose and treatment changes at follow-up visits were made on the basis of treatment response and in accordance with routine clinical practice.

We aimed to complete recruitment over a 4·5 year period and to then follow up the trial cohort for a further 2 years, allowing a minimum follow-up of 2 years and maximum of 6·5 years. Patients were followed up according to clinical need, and minimum trial visits were expected at 3, 6, and 12 months, and annually thereafter. At visits, data were collected for seizures, anti-seizure medication, and adverse reactions. Participants continued in follow-up whether they were still taking their allocated treatment or not. When participants fell out of hospital follow-up, outcome data were sought from their general practitioner.

For adults, QOL outcomes were assessed using subscales of the quality of life in newly diagnosed epilepsy battery (NEWQOL) and the Impact of Epilepsy Scale.^24^ For children and adolescents aged younger than 16 years, QOL assessment involved both patient and parent-based measures: children aged 8–15 years completed a generic health status measure validated for use in epilepsy, the KINDL;^25^ and the epilepsy impact and attitude to epilepsy subscales of the Quality of Life in Epilepsy Inventory for Adolescents (QOLIE-AD).^26^ Parents of all children completed proxy QOL questionnaires. QOL questionnaires were completed at baseline and annually thereafter. Adults and parents also completed a subset of QOL measures at 3 months and 6 months.

Adult and adolescent participants were asked to complete the EQ-5D-3L and the EuroQol visual analogue scale (EQ-VAS); participants aged 8–15 completed the EQ-VAS and self-reported youth EQ-5D-3L-Y, or if not available, proxy EQ-5D-3L, which was completed by a parent or carer. For participants aged 5–7 years, only proxy questionnaires were administered. Participants' resource-use associated with secondary care (ie, inpatient, outpatient, and accident and emergency care), other health-care and social services (ie, primary care and community services), and medicines were measured using routine hospital episode statistics, resource-use questionnaires,^27^ and case report form records. Resource-use was valued in monetary terms (measured in pounds sterling using national unit costs for 2019–20) using national unit costs.^27^, ^28^, ^29^, ^30^

### Outcomes

The primary outcome was time to 12-month remission from seizures, calculated as days from randomisation to the first date at which a period of 12 months had elapsed without any seizures. The secondary seizure outcomes were time to 24-month remission and time to first subsequent seizure. There were three secondary outcomes for treatment failure: (1) time to treatment failure overall, defined as days from randomisation to a decision to withdraw the randomised drug or to add a new anti-seizure medication because of either inadequate seizure control or unacceptable adverse reactions; (2) time to treatment failure due to inadequate seizure control alone; and (3) time to treatment failure due to unacceptable adverse reactions alone. The other secondary outcomes were adverse reactions, QOL, and health economic outcomes based on incremental costs and quality-adjusted life-years (QALYs) gained. At clinic visits, data were collected on adverse reactions reported by the patient and the investigators assessment stated whether the event was possibly, probably, or almost certainly related the anti-seizure medication. These adverse reactions were coded using the MedDRA dictionary.

### Statistical analysis

SANAD-II was designed to detect non-inferiority of levetiracetam compared with valproate for the primary outcome of time to 12-month remission. The International League Against Epilepsy commission on antiepileptic drugs defined limits of equivalence of ±10% for the primary outcome in antiepileptic drug monotherapy studies.^28^ Calculations were informed by the SANAD I study,^14^ which estimated the 12-month remission free probability (at 24 months) as 0·31 (exponential hazard rate of 0·0488) for valproate. Assuming a 10% absolute difference in survival probability, the non-inferiority margin on the hazard ratio (HR) scale was ln(0·31)/ln(0·41)=1·314. Therefore, assuming a HR of 1, 80% power, and a one-sided alpha of 0·025, 260 patients were required in each of two treatment groups, allowing for 5% losses to follow-up, as occurred in SANAD-I (520 patients in total).

Primary analyses were undertaken on an intention-to-treat (ITT) basis. We used a 0·05 level of significance and 95% CIs throughout. The statistical and health economic analysis plans were developed before doing final analyses and are available in appendix 1 and appendix 2. Analyses were done using SAS software (version 9.4; SAS Institute, Cary, NC, USA). Completeness of follow-up statistics were calculated as the total number of days follow-up for all participants as a percentage of the total potential number of days follow-up.^29^

Time to event outcomes were summarised by Kaplan-Meier curves for each treatment group and Cox proportional hazards regression models explored using two different models: (1) the primary analysis, including the treatment effect only; and (2) including the treatment together with gender (ie, male or female), number of seizures before randomisation (ie, 2, 3–5, 6+), and random effects for centre. Models were also fitted to include a stratification variable for epilepsy type (ie, generalised or unclassified epilepsy). The assumption of proportional hazards was investigated by examining Schoenfeld residual plots and incorporating time-dependent covariates in all models. If the assumption of proportional hazards was not valid, an additional extended Cox model with time-dependent covariates was used. Subgroup effects (for patients with absence epilepsies, other generalised epilepsies, or unclassified epilepsy) were explored in a post-hoc analysis by adding treatment-covariate interaction terms to the primary Cox model. All treatment effects were presented as a HR with a two-sided 95% CI of valproate compared with levetiracetam. For the primary outcome (ie, 12-month remission) non-inferiority hypothesis, the upper limit of the 95% CI needed to be less than 1·314 to conclude non-inferiority.

A per-protocol (PP) analysis of the primary outcome was also done using a Fine and Gray model,^30^ with treatment failure included as a competing risk, and censoring participants with drug failure before achieving remission. This analysis excluded participants with major protocol deviations, participants given an alternative diagnosis to epilepsy after random assignment, and participants who did not receive the drug at all.

For time to treatment failure, a competing risks analysis, using the Fine and Gray model,^30^ was done to assess the two main reasons for treatment failure (ie, inadequate seizure control and unacceptable adverse reactions).^31^ Cumulative incidence curves are presented for each treatment group.

The difference in QOL measures between treatment groups was estimated for each population (ie, children, adults, and parent-carers) and for each outcome applicable within that population by fitting a repeated measures random effects model with a baseline QOL variable, treatment group, and time in days using spatial-power covariance structure for repeated measures and unstructured covariance for the random effect.

Analysis sets for the summary of adverse reactions included all patients who received any dose of a study drug. All adverse reactions and serious adverse reactions were coded using the MedDRA dictionary. The number (and percentage) of patients experiencing each reaction, and the number (and percentage) of occurrences of each reaction are presented with no formal statistical testing undertaken.

Interim monitoring was done by an independent data safety monitoring committee, meeting approximately annually. This process included analyses of the primary outcome and five of the secondary outcomes (all using the Haybittle-Peto approach).

The economic analysis (shown in appendix 2) adopted the costing perspective of the NHS and personal social services and was done using data up to 24 months of follow-up. Missing cost and QALY data were imputed using multiple imputation with chained equations.^32^ Based on the imputed data, differences between treatment groups in total costs and QALYs were compared with reference to bootstrapped central ranges, based on 10 000 replications. In the base-case analysis, total costs and QALYs (with year 2 discounted at 3·5%) were adjusted using linear regressions^33^ for treatment allocation, baseline costs or utility, age, sex, and epilepsy classification, with centre as random effects. Incremental costs and QALYs were estimated to identify dominance and calculate the incremental net health benefit as the difference in QALYs between treatments, minus the difference in costs multiplied by the cost-effectiveness threshold (£20 000 per QALY).^34^ The joint uncertainty in incremental costs and QALYs was expressed in terms of the probability of each treatment being cost-effective at the threshold. Sensitivity analyses comprised alternative discount rates, use of complete cases, PP cohort, QALYs based on the NEWQOL-6D^35^ and EQ-VAS, and were based on unadjusted analysis. A subgroup analysis considered cost-effectiveness in children, adults, and adolescents aged 16 years or older at the point of randomisation. QALYs were calculated on the basis of the area under the curve of utility data measured using the EuroQol 5-dimension 3-level (EQ-5D-3L) questionnaire, and applying the UK tariff scores.^25^ This trial is registered with the ISRCTN registry, 30294119 (EudraCt Number: 2012-001884-64).

### Role of the funding source

The funder of the study had no role in study design, data collection, data analysis, data interpretation, or writing of the report.

## Results

**Figure 1:**
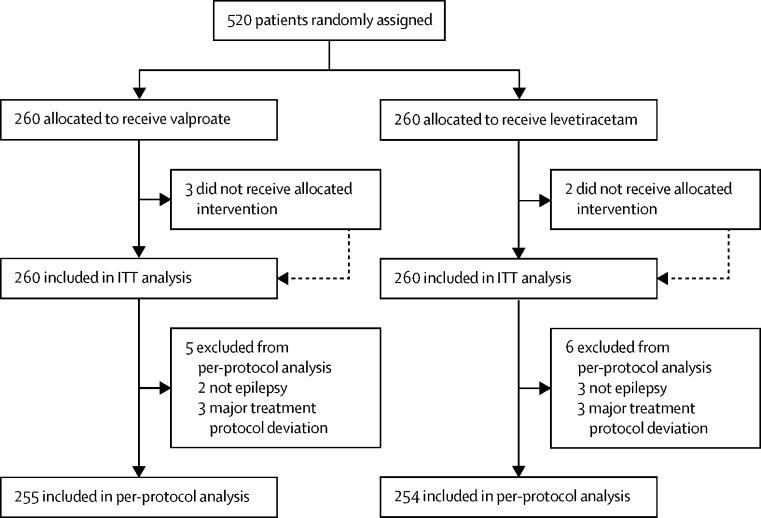
Trial profile Data on non-randomised patients were not collected. ITT=intention-to-treat.

The first participant was randomly assigned on April 30, 2013, and the last participant was randomly assigned on Aug 2, 2016, after which every effort was made to follow up the trial cohort for a further 2 years. The last participant visit was on Jan 13, 2019. 69 UK centres recruited between one and 40 patients each and randomly assigned a total of 520 participants, 260 to start treatment with levetiracetam and 260 to start treatment with valproate (figure 1). Baseline characteristics were well balanced across treatment groups (table 1). The median age of participants was 13·9 years (IQR 8·9–19·7) with a predominance of male participants (65%) showing concern about randomly assigning female participants to valproate. Approximately 10% of participants (51 participants) had a learning disability, 16 (3%) participants had a neurological deficit, and 99 (19%) participants had a first degree relative with epilepsy. Approximately three-quarters of the participants (397 participants) had generalised epilepsy and the remainder of the participants (123 participants) had unclassifiable epilepsies. Of those with generalised epilepsy, 104 (26·2%) had childhood absence epilepsy, 36 (9·1%) had juvenile absence epilepsy, 51 (12·8%) had juvenile myoclonic epilepsy, 23 (5·8%) had generalised epilepsy with tonic-clonic seizures on waking, and 180 (45·3%) were classified as idiopathic generalised epilepsy not specified. Participants were randomly assigned a median of 4 days (0–26) after their most recent seizure.

**Table 1:** Baseline characteristics

	**Valproate (n=260)**	**Levetiracetam (n=260)**	**Total (n=520)**
**Age, years**			
Median (IQR)	13·6 (8·8–19·7)	14·1 (9·1–19·8)	13·9 (8·9–19·7)
Range	5·0–94·4	5·0–83·9	5·0–94·4
**Age group, years**			
5–7	52 (20%)	48 (18%)	100 (19%)
8–11	54 (21%)	56 (22%)	110 (21%)
12–15	54 (21%)	48 (18%)	102 (20%)
16–29	70 (27%)	81 (31%)	151 (29%)
≥30	30 (12%)	27 (10%)	57 (11%)
**Gender**			
Male	167 (64%)	170 (65%)	337 (65%)
Female	93 (36%)	90 (35%)	183 (35%)
**Previous or current neurological disorder**			
Stroke or cerebrovascular	0	0	0
Cerebral haemorrhage	0	2 (1%)	2 (<1%)
Intracranial surgery	0	2 (1%)	2 (<1%)
Head injury*	1 (<1%)	1 (<1%)	2 (<1%)
Meningitis or encephalitis	4 (2%)	0	4 (1%)
Cortical dysplasia or developmental anomaly	0	0	0
Other	11 (4%)	13 (5%)	24 (5%)
**History**			
Learning disability	22 (8%)	29 (11%)	51 (10%)
Febrile convulsions	21 (8%)	23 (9%)	44 (8%)
Any other acute symptomatic seizures	4 (2%)	10 (4%)	14 (3%)
Family history in primary relatives	49 (19%)	50 (19%)	99 (19%)
Neurological deficit	6 (2%)	10 (4%)	16 (3%)
**Epilepsy type**			
Generalised epilepsy	201 (77%)	196 (75%)	397 (76%)
Unclassified epilepsy	59 (23%)	64 (25%)	123 (24%)
**Epilepsy syndrome (generalised epilepsy only)**†			
Childhood absence	52 (26%)	52 (27%)	104 (26%)
Juvenile absence	22 (11%)	14 (7%)	36 (9%)
Juvenile myoclonic	24 (12%)	27 (14%)	51 (13%)
Epilepsy with tonic-clonic seizures on awakening	11 (5%)	12 (6%)	23 (6%)
Other idiopathic generalised epilepsy not specified‡	90 (45%)	90 (46%)	180 (45%)
Other epilepsy syndrome	10 (5%)	7 (4%)	17 (4%)
**Total number of seizures reported**			
Median	10 (3–99+)	10 (3–99+)	10 (3–99+)
Missing	1	5	6
**Days since most recent seizure**			
Median	4 (0–26)	4 (0–25)	4 (0–26)
Range	0–223	0–211	0–223
Missing	2	5	7
**Age at first seizure, years**			
Median	12 (7·2–18)	13 (8·3–18)	13 (7·8–18)
Range	0·5–93	0·2–80	0·2–93
Missing	3	7	10
**Interval between first and most recent seizure, days**			
Median	203 (98–665)	250 (110–603)	228 (100–648)
Range	0–16 136	0–19 662	0–19 662
Missing	3	7	10
**EEG**			
EEG not done	20 (8%)	24 (9%)	44 (8%)
EEG normal	58 (22%)	51 (20%)	109 (21%)
Non-specific abnormality	11 (4%)	9 (3%)	20 (4%)
Generalised abnormality (slow wave activity with spiking)	138 (53%)	133 (51%)	271 (52%)
Generalised abnormality (slow wave activity without spiking)	8 (3%)	7 (3%)	15 (3%)
Focal abnormality (paroxysmal slow activity with spiking)	10 (4%)	8 (3%)	18 (3%)
Focal abnormality (paroxysmal slow activity without spiking)	2 (1%)	7 (3%)	9 (2%)
Other	13 (5%)	21 (8%)	34 (7%)

Data are n (%) or median (IQR), unless otherwise stated. EEG=electroencephalogram.

* Post-traumatic amnesia lasting over 24 h or compound depressed fracture.

† More than one category could be selected.

‡ 150 (83%) of 180 patients in this group reported tonic-clonic seizures.

The completeness of follow-up statistics for the primary outcome was 87% for valproate and 83% for levetiracetam. In this analysis, the median days of follow-up was 427 (IQR 365–731) for valproate and 550 (366–781) for levetiracetam; follow-up was shorter for valproate as participants allocated valproate achieved the primary outcome sooner. Estimates for the primary and secondary analyses are provided in appendix 3 (p 1).

**Figure 2:**
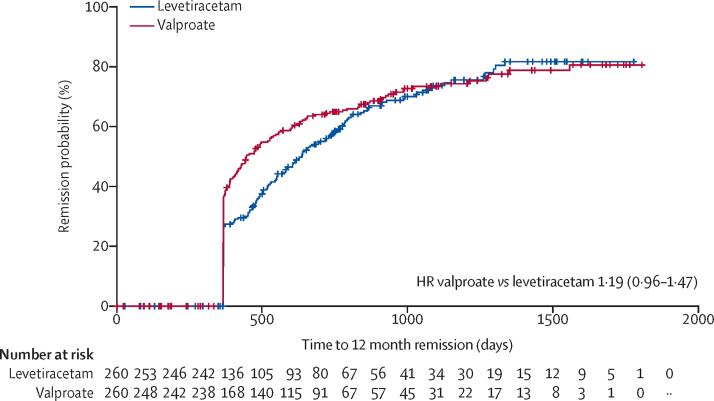
Kaplan Meier plot of time to 12-month remission: levetiracetam versus valproate, intention to treat analysis HR=hazard ratio.

The ITT analysis of time to 12-month remission did not find non-inferiority of levetiracetam compared with valproate as the 95% CI for the HR (1·19 [95% CI 0·96–1·47] unadjusted, 1·23 [0·99–1·52] adjusted) includes the pre-defined non-inferiority margin of 1·314. Consequently, the possibility of a clinically important difference could not be excluded. There is crossing of the Kaplan-Meier survival curves (figure 2) and evidence of non-equality of hazard ratios across time (p=0·001), violating the assumption of proportional hazards. Interval specific HR estimates (appendix 3 p 1) indicate significant benefit of valproate within the first year, but not in subsequent years. Annual differences in 12-month remission probabilities, for example, that at 1 year, 9% fewer patients had entered 12-month remission on levetiracetam than on valproate are shown in appendix 3 (p 2). The Kaplan-Meier estimate of the median time to achieve 12-month remission was also shorter for valproate (445 days [95% CI 406–531]) than for levetiracetam (636 days [553–728]).

The PP analyses (Fine and Gray model) for time to 12-month remission (appendix 3 p 3) excluded patients with major protocol deviations (6; 1%) and patients whose epilepsy diagnosis was a misdiagnosis (5; 1%) and accounted for treatment failures before achieving 12-month remission (82 [32%] participants in the valproate group and 121 [47%] participants in the levetiracetam group). The per-protocol analysis showed that 12-month remission was superior with valproate than with levetiracetam; although this trial was powered for non-inferiority, it was also designed to show superiority. Furthermore, the assumption of a constant HR across time appeared reasonable in the PP analysis, suggesting that treatment failures before remission largely explain the non-constant effect seen in the ITT analysis.

Subgroup effects were explored in a post-hoc analysis (appendix 3 p 4) and indicated an important advantage for initiating valproate in participants with other idiopathic generalised epilepsies (HR 1·55 [95% CI 1·14–2·11]) whereby the difference in immediate 12-month remission rates were 19·1% (6·6–31·7), but not for absence epilepsies (HR 0·90 [0·60–1·35]), or unclassified epilepsy (1·07 [0·69–1·67])

Valproate was shown to be superior to levetiracetam for time to 24-month remission (using ITT analysis; HR 1·43 [95% CI 1·06 to 1·92]). Again, there was a crossing of the Kaplan-Meier survival curves (appendix 3 p 5) and evidence against an assumption of proportional hazards (p=0·002). At 24 months follow-up, the difference in 24-month remission rates was 12% (4 to 20), diminishing to 4% (–8 to 17) at 4 years.

**Figure 3:**
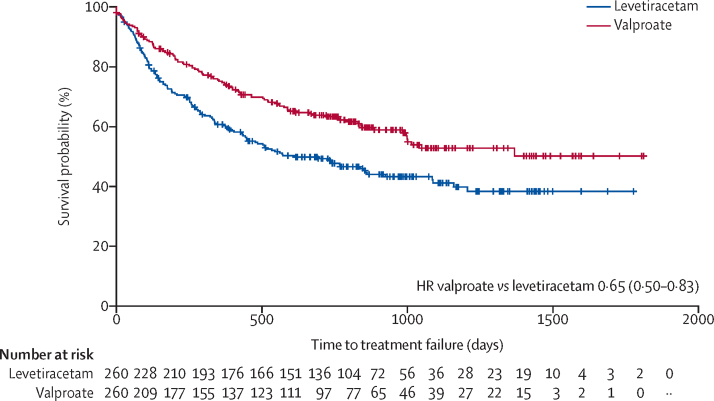
Kaplan-Meier plot of time to treatment failure: levetiracetam versus valproate HR=hazard ratio.

Valproate was superior to levetiracetam for time to first seizure (HR 0·82 [95% CI 0·67–1·00]; appendix 3 p 6) and there was insufficient evidence against an assumption of proportional hazards (p=0·39). Valproate was also superior to levetiracetam for time to treatment failure for any reason (figure 3; 0·65 [0·50–0·83]) with insufficient evidence against an assumption of proportional hazards (p=0·22). Annual treatment failure rates and differences in failure rates between valproate and levetiracetam are shown in appendix 3 (p 7). At 2 years there was a 15% (6–23) difference in the treatment failure rate for levetiracetam compared with valproate. The doses taken at treatment failure or at the point of last follow-up are summarised in appendix 3 (p 8), and the findings indicate that reasonable dose ranges were tried before deciding failure had occurred.

Analysis of the two main reasons for treatment failure found valproate to be superior to levetiracetam for treatment failure due to inadequate seizure control (HR 0·43 [95% CI 0·30–0·63]), but no difference between groups was found for treatment failure due to unacceptable adverse reactions (0·93 [0·61–1·40]; appendix 3 p 9.

SANAD II recorded data for adverse reactions that were judged by the treating clinicians to be possibly, probably, or definitely caused by anti-seizure medicines. The safety analysis included the 258 participants who were randomly assigned to levetiracetam and the 257 participants who were randomly assigned to valproate and who received at least one dose of their allocated treatment. Adverse reactions according to the MedRA system organ classification are shown in table 2, and adverse reactions by MedRA preferred term are given in appendix 3 (p 10). There were 220 adverse reactions in 96 (37%) participants who were randomly assigned to valproate and 223 adverse reactions in 107 (41%) participants who were randomly assigned to levetiracetam (table 2). There were more psychiatric symptoms reported in participants allocated to levetiracetam (109 events reported in 66 [26%] participants) compared with valproate (54 events reported in 36 [14%] participants). There were more reports of weight gain with valproate (26 [10%] participants) than with levetiracetam (eight [3%] participants); appendix 3 p 10. Of those randomly assigned to valproate, ten (4%) participants had a total of 15 severe adverse reactions and of those randomly assigned to levetiracetam, ten (3·9%) participants had a total of 16 severe adverse reactions (appendix 3 p 13). For two (1%) patients who were randomly assigned to valproate and four (2%) patients who were randomly assigned to levetiracetam, the adverse reactions were classified as serious. None were classified as suspected unexpected serious adverse reactions. There were two deaths, one in each group, that were unrelated to trial treatments. One participant randomly assigned to initiate valproate became pregnant. The pregnancy was conceived while taking levetiracetam monotherapy and resulted in a healthy baby without any malformations at postnatal examination. Nine participants randomly assigned to levetiracetam reported a pregnancy with four healthy babies at postnatal examination, three miscarriages (all of whom were taking levetiracetam at the time of reporting pregnancy), one baby with low birthweight (levetiracetam taken at the time of reporting pregnancy) and one baby with major malformations (carbamazepine taken at the time of reporting pregnancy).

**Table 2:** Adverse reactions by the MedRA system organ classification

	**Valproate events**	**Levetiracetam events**	**Valproate patients (n=257)**	**Levetiracetam patients (n=258)**
Psychiatric disorders	54	109	36 (14%)	66 (26%)
Nervous system disorders	58	46	42 (16%)	37 (14%)
Gastrointestinal disorders	24	20	19 (7%)	15 (6%)
Investigations	31	11	29 (11%)	11 (4%)
General disorders and administration site conditions	20	17	16 (6%)	15 (6%)
Metabolism and nutrition disorders	19	8	19 (7%)	8 (3%)
Skin and subcutaneous tissue disorders	11	6	11 (4%)	5 (2%)
Blood and lymphatic system disorders	1	1	1 (<1%)	1 (<1%)
Eye disorders	1	1	1 (<1%)	1 (<1%)
Respiratory, thoracic, and mediastinal disorders	0	2	0	2 (1%)
Congenital, familial, and genetic disorders	0	1	0	1 (<1%)
Immune system disorders	1	0	1 (<1%)	0
Injury, poisoning, and procedural complications	0	1	0	1 (<1%)

221 participants returned a baseline questionnaire and at least one follow-up questionnaire and were included in the QOL analysis. For participants who provided QOL data, the mean follow-up time was 695 days (SD 445) and the maximum was 1883 days. Participants who were not included in this analysis were more likely to be male than female (71% *vs* 57%) and have unclassified epilepsy than generalised epilepsy (27% *vs* 19%). Results from the repeated measures random effects models (appendix 3 p 14) suggested there might be small differences in favour of levetiracetam for QOL emotional (child), family (child and parent), and school (child) domains. However, because of the high level of missing data, these results cannot be considered reliable. We did not consider imputation reasonable because of the high level of missing data.

Health economic analysis data were available for 412 participants, and self-reported resource-use data were available for 243 participants at 3 months, 212 participants at 6 months, 185 participants at 12 months, and 148 participants at 24 months. Most of the costs were related to hospital outpatient clinic attendance, admitted care, and anti-seizure medications (appendix 3 p 15). Total, unadjusted costs for participants randomly assigned to levetiracetam were £4267 (95% central range [CR] 3944 to 5462), compared with £4205 (3827 to 4956) for valproate. The difference of £61 (–651 to 1230) was not significant. In the adjusted, base-case analysis, total costs were £4350 (4136 to 5623) for levetiracetam, compared with £4246 (3979 to 5090) for valproate (table 3). These results correspond to an incremental cost of £104 (–587 to 1234).

**Table 3:** Results of the adjusted base-case and subgroup analyses

	**Levetiracetam**	**Valproate**	**Incremental**
**Base-case all participants (n=520)**			
Total costs (£)	4350 (4136 to 5623)	4246 (3979 to 5090)	104 (−587 to 1234)
QALYs	1·603 (1·500 to 1·631)	1·637 (1·565 to 1·673)	−0·035 (−0·103 to 0·077)
Net health benefit at £20 000 per QALY (QALYs)	1·385 (1·236 to 1·410)	1·425 (1·323 to 1·464)	−0·040 (−0·175 to 0·037)
**Children aged <16 years (n=312)**			
Total costs (£)	4336 (4017 to 5516)	4360 (4046 to 5149)	−24 (−752 to 1065)
QALYs	1·624 (1·506 to 1·646)	1·626 (1·554 to 1·667)	−0·002 (−0·123 to 0·054)
Net health benefit at £20 000 per QALY	1·407 (1·254 to 1·430)	1·408 (1·307 to 1·455)	−0·002 (−0·136 to 0·062)
**Adults and adolescents aged ≥16 years (n=208)**			
Total costs (£)	4316 (3842 to 5898)	3957 (3525 to 5161)	359 (−644 to 1640)
QALYs	1·576 (1·474 to 1·636)	1·654 (1·563 to 1·693)	−0·078 (−0·175 to 0·015)
Net health benefit at £20 000 per QALY	1·407 (1·200 to 1·425)	1·456 (1·330 to 1·497)	−0·090 (−0·208 to 0·018)

Data are mean (95% CI). QALY=quality-adjusted life-years.

EQ-5D utilities were available for 274 participants at baseline, and they could be calculated for 161 participants at 12 months and for 128 participants at 24 months. Levetiracetam was associated with 1·603 QALYs (1·500 to 1·631) in the base-case analysis, compared with 1·637 QALYs (1·565 to 1·673) for valproate. This difference corresponded to an incremental QALY of −0·035 QALYs (–0·137 to 0·032). Levetiracetam was therefore dominated by valproate and was associated with a negative incremental net health benefit of −0·040 QALYs (–0·175 to 0·037) at a cost-effectiveness threshold of £20 000 per QALY. The probability of levetiracetam being cost-effective at this threshold was 0·17. Incremental net health benefits were similarly negative for both subgroups (table 3). Sensitivity analyses are provided in appendix 4 (pp 26–30).

## Discussion

SANAD II found that levetiracetam is neither clinically effective nor cost-effective compared with valproate in patients with newly diagnosed generalised or unclassified epilepsy. This pragmatic, multicentre, open-label, randomised trial was powered to assess non-inferiority of levetiracetam compared with standard treatment, valproate, in patients with newly diagnosed generalised and unclassified epilepsy. Levetiracetam did not meet our non-inferiority definition for time to 12-month remission from seizures. We found a 9% (95% CI 1–18) higher immediate 12-month remission rate with valproate (26% *vs* 36%), but the difference between treatment policies diminished over time as dose and treatment changes were made. Levetiracetam was inferior to valproate for time to treatment failure, time to 2 year remission, and time to first subsequent seizure. Treatment failure due to inadequate seizure control was more likely with levetiracetam than with valproate (HR 0·43 [0·30–0·63]). Inadequate seizure control leading to treatment change was the likely explanation for finding non-proportional hazards in the primary ITT 12-month remission analysis. The PP analysis, which took treatment failure into account, found valproate to be superior to levetiracetam (1·68 [1·30–2·15]). These results are particularly important when considering treatment choices for women of childbearing potential.

To explore the treatment effects further, the cohort was split into three groups: participants with absence epilepsies, participants with other generalised epilepsies, and participants with unclassified epilepsy. For time to 12-month remission, our results indicated a meaningful advantage for starting valproate in the so-called other generalised epilepsy group, for which the difference in immediate remission rate was 19·1% (95% CI 6·6–31·7), whereas there was no clear advantage seen in the absence or unclassified epilepsy subgroups. Participants with other generalised epilepsies were mainly those with generalised tonic-clonic seizures, for whom seizure rates are low and many months of observation are typically required to record seizures and make incremental changes to dose and drug. Conversely, participants with absence seizures typically have a high seizure rate, enabling more rapid decisions about dose and drug changes to gain early seizure control.

The number of female participants recruited (n=80) between the age of 12 and 50 years was lower than the number of men recruited (n=218) and the European Medicines Agency and the Medicines and Healthcare Products Regulatory Agency pregnancy prevention scheme was implemented following the commencement of the study and during most of its recruitment and follow-up period. There were ten pregnancies during the study, none of which were exposed to valproate, and only one of these women was randomly assigned to initiate valproate.

Analysis of QOL outcomes did not indicate benefit for either drug, but the return rate of questionnaires was disappointingly low. The cost utility analysis found that levetiracetam was not cost-effective compared with valproate at thresholds of cost-effectiveness operating in the UK NHS. Levetiracetam was associated with fewer QALYs and higher costs than valproate. The finding of a negative incremental net health benefit for levetiracetam compared with valproate was consistent for both adult and children subgroups and was stable in most sensitivity analyses, apart from two that were limited by the missing data in NEWQOL-6D utilities and costs.

This study has several important limitations. Data for the occurrence of seizures were collected using seizure diaries and reports at clinic visits and it is possible that seizures were missed or not reported. SANAD II was open-label, which might have influenced decisions about dose and treatment changes, thereby biasing results for time to treatment failure, seizure outcomes, and the reporting of adverse reactions. Although 397 (76%) participants were classified as having a generalised epilepsy, only 271 (52%) had generalised spike and wave changes on their EEG, indicating that some of the remaining 24% of participants might have been misclassified. It is not possible to state whether this factor might have increased or diminished the treatment effects observed, but it is interesting to note that in the subgroup analysis for 12-month remission the estimate in participants who were unclassified favoured levetiracetam. In addition, other than for participants with absence epilepsies, the number of participants classified with a specific generalised epilepsy syndrome at the time of random assignment was small, precluding subgroup analyses for syndromes, such as juvenile myoclonic epilepsy.

Additionally, more men than women were recruited (64·8% *vs* 35·2%), which could have introduced unintended bias into the study. Although there is no reason to expect important differences in clinical effectiveness by gender,^7^ differing approaches to dosing and treatment choices for men and some women could influence seizure remission rates, adverse events, and decisions about treatment failure. There was also a low return rate for questionnaires, which diminished our ability to identify QOL consequences and also affected the economic analysis. For costs, the use of free text questions might have introduced bias, as also the assumption that unanswered questions implied no use of resources. However, these issues were largely mitigated by more complete data for the costs of hospital care and anti-seizure medicines, which were more difficult to recall while also being the main cost drivers. For QALYs, this issue was mitigated by use of area under the curve methodology, in which QALYs could be calculated provided two or more EQ-5D questionnaires had been returned. Our use of the EQ-5D-3L-Y and proxy version of the EQ-5D-3L was limited by having to apply the adult tariff for estimating utilities from EQ-5D profiles. This requirement represents a weakness in many economic evaluations of interventions in paediatric populations,^36^ although a valuation of children's EQ-5D-3L-Y health states should soon be available.^37^

These results should be put into context with previous studies, although few studies have assessed the long term effectiveness of treatments for patients with generalised epilepsies. SANAD I^14^ identified valproate as a first-line treatment as it was superior to lamotrigine for seizure control and superior to topiramate for treatment failure.^10^ An individual participant data network meta-analysis,^13^ which included data from SANAD I, failed to show superiority for 12-month remission in participants with generalised epilepsy of any drug among valproate, levetiracetam, gabapentin, phenytoin, carbamazepine, oxcarbazepine, topiramate, or phenobarbital, but the results were heavily confounded by classification errors and the possible inclusion of participants with focal epilepsy. Valproate was superior to carbamazepine, topiramate, and phenobarbital for treatment failure. A clinical trial^14^ in participants with absence epilepsies found valproate and ethosuximide superior to lamotrigine for treatment failure. To the best of our knowledge, SANAD II is the only trial that provides much needed head-to-head data for the long term effectiveness of valproate versus levetiracetam.

These results have important implications for clinical practice and research. For men with generalised onset seizures, valproate should continue as a first-line treatment. For women of childbearing potential, levetiracetam was inferior to valproate, as is lamotrigine,^14^ the other commonly prescribed alternative. Regulators, guideline developers, clinicians, and patient groups should now consider the benefit-to-risk ratio of each treatment. Some women might prefer a drug with greater efficacy notwithstanding the risk of teratogenicity, while others might prefer one that is safer in pregnancy despite lower efficacy, as indicated in a previous discrete choice experiment,^38^ which found that women would accept a 5% reduction in 12-month remission probability for a 1% reduction in fetal abnormality. For people with unclassified epilepsy, the subgroup analysis found no significant difference between treatments, but estimates favour levetiracetam. Future studies should not group generalised and unclassified epilepsy together and the international epilepsy community should identify a better strategy for assessing treatment policies in this common scenario.

## Data sharing

All requests for data sharing should be addressed to the trial co-sponsors. Co-sponsors will process the requests by involving all applicable parties in their decision making outcome (eg, joint data controllers and pharmaceutical companies). Data sharing packs are prepared at the end of trial and involve as a minimum all versions of the trial protocol, all versions of the annotated trial case report forms, and dataset.

## Declaration of interests

AM reports grants from the National Institute for Health Research Health Technology Assessment, during the conduct of the study, as well as grants from UCB Pharma, outside of the submitted work. JPL reports grants from University of Liverpool during the conduct of the study; grants and personal fees from UCB Pharma; and personal fees from Eisai, Janssen CIlag Pharmaceuticals, GW Pharmaceuticals, GSK Pharma, outside of the submitted work. GS reports personal fees from UCB Pharma, Eisai, Arvelle Therapeutics GmbH, outside of the submitted work. CP reports grants from National Institute for Health Research Health Technology Assessment Programme during the conduct of this study. CT reports grants from the National Institute for Health Research, during the conduct of the study. DH reports grants from National Institute for Health Research Health Technology Assessment Programme during the conduct of the study. RM reports personal fees from UCB Pharma and grants from UCB Pharma and Sanofi, outside of the submitted work. PES is a member of the NICE Panel for Epilepsy guideline 2021 and is an editor of the journal Practical Neurology. All other authors declare no competing interests.
