# Department of Error

**DOI:** 10.1016/S0140-6736(21)00886-2

**Published:** 2021-04-24

*Marson A, Burnside G, Appleton R, et al. The SANAD II study of the effectiveness and cost-effectiveness of valproate versus levetiracetam for newly diagnosed generalised and unclassifiable epilepsy: an open-label, non-inferiority, multicentre, phase 4, randomised controlled trial.* Lancet *2021; **397:** 1375–86*—Appendices 3 and 4 of this Article have been corrected as of April 22, 2021.

