# Department of Error

**DOI:** 10.1016/S0140-6736(21)01011-4

**Published:** 2021-05-15

*Marson A, Burnside G, Appleton R, et al. The SANAD II study of the effectiveness and cost-effectiveness of valproate versus levetiracetam for newly diagnosed generalised and unclassifiable epilepsy: an open-label, non-inferiority, multicentre, phase 4, randomised controlled trial.* Lancet *2021; **397:** 1375–86*—In this Article, N Vora should not have been included in the SANAD II collaborators list. This correction has been made to the online version as of May 13, 2021.

