# Supplementary material for: The SANAD II study of the effectiveness and cost-effectiveness of valproate versus levetiracetam for newly diagnosed generalised and unclassifiable epilepsy: an open-label, non-inferiority, multicentre, phase 4, randomised controlled trial
Source: Lancet. 2021 Apr 10;397(10282):1375–86. doi: 10.1016/S0140-6736(21)00246-4 (PMC8047813; doi:10.1016/S0140-6736(21)00246-4)
Supplement: Supplementary appendix 1 [file mmc1.pdf]

THE LANCET

Supplementary appendix

This appendix formed part of the original submission and has been peer reviewed. We post it as supplied by the authors.

Supplement to: Marson A, Burnside G, Appleton R, et al. The SANAD II study of the effectiveness and cost-effectiveness of valproate versus levetiracetam for newly diagnosed generalised and unclassifiable epilepsy: an open-label, non-inferiority, multicentre, phase 4, randomised controlled trial. *Lancet* 2021; **397:** 1375–86.

A pragmatic randomised controlled trial comparing the effectiveness and cost effectiveness of levetiracetam and zonisamide versus standard treatments for epilepsy: a comparison of Standard And New Antiepileptic Drugs (SANAD-II)

Eudract No. 2012-001884-64

Trial registration: ISRCTN 30294119

Statistical Analysis Plan
Version 1.0 28/01/2019

	**ORIGINATED BY**	**APPROVED BY**
**Name**	Barbara Arch	Paula Williamson
**Title**	Statistician	Professor of Biostatistics
**Date**	28/01/2019	
**Protocol Version and Date**	V8 28/11/2018	

1 Change Control

Protocol version	Updated SAP version no.	Section number changed	Description of change	Date changed

2 Approval and agreement

SAP Version Number being approved: 1.0

Trial Statistician* [Trial statistician has seen unblinded data so has not written this SAP. Duty delegated to independent statistician who has not seen unblinded data.]

Name Barbara Arch

Signed ______________________________ Date __________

Senior Statistician* [Senior statistician has seen unblinded data so has not written this SAP. Duty delegated to independent statistician who has not seen unblinded data.]

Name Paula Williamson

Signed ______________________________ Date __________

Chief Investigator/clinical lead

Name ____

Signed ______________________________ Date __________

OR Electronic approval attached ☐

3 Roles and responsibilities

B Arch (Department of Biostatistics, University of Liverpool): Statistician; P Williamson: Professor of Biostatistics (Department of Biostatistics, University of Liverpool); T Marson Professor of Neurology, University of Liverpool: Chief Investigator.

Author's contributions

B Arch proposed the statistical analysis plan (SAP) building on the outlined analyses set out in the trial protocol, and drafted the manuscript. P Williamson and T Marson read, amended and approved the SAP.

4 List of abbreviations and definitions of terms

AED	Anti-epileptic drug
AR	Adverse reaction
CRF	Case report form
EEG	Electroencephalogram
HR	Hazard ratio
IDSMC	Independent Data and Safety Monitoring Committee
ISC	Inadequate seizure control
IQR	Inter-quartile range
LLT	Lower level term
MedDRA	Medical Dictionary for Regulatory Activities
PT	Preferred term
QOL	Quality of Life
SAE	Serious adverse event
SAR	Serious adverse reaction
SD	Standard deviation
SOC	System organ classification
SUSAR	Suspected unexpected serious adverse reaction
TF	Treatment failure
TSC	Trial steering committee
UAE	Unacceptable adverse event

Contents

1 Change Control ... 1
2 Approval and agreement ... 2
3 Roles and responsibilities ... 3
4 List of abbreviations and definitions of terms ... 4
5 Statement of Compliance ... 7
6 Background and Rationale ... 8
7 SANAD-II Study Objectives ... 8
7.1 Arm A: Patients with focal onset seizure ... 8
7.2 Arm B: Patients with generalised onset seizures or seizures that are difficult to classify ... 9
8 Investigational Plan and Study Design ... 10
8.1 Overall study design and plan - description ... 10
8.2 Treatments studied ... 10
8.3 Treatment compliance ... 10
8.4 Patient population studied ... 10
8.4.1 Inclusion criteria ... 11
8.4.2 Exclusion criteria ... 11
8.5 Removal of patients from therapy or assessment ... 11
8.6 Consent process ... 11
8.7 Blinding ... 11
8.8 Method of assignment to treatment ... 12
8.9 Sequence and duration of all study periods ... 12
9 Listing of Outcomes ... 12
9.1 Primary outcome ... 12
9.2 Secondary outcomes ... 12
10 Determination of Sample Size ... 13
11 Study Framework ... 13
12 Confidence Intervals, p-values and Multiplicity ... 13
13 Timing and Objectives of Interim and Final Analyses ... 14
13.1 Interim monitoring and analyses ... 14
13.2 Final analysis ... 14
14 Disposition of Participants ... 14
14.1 Screening, eligibility and recruitment ... 14
14.2 Post randomisation discontinuations ... 17
14.2.1 Completeness of follow-up analysis ... 17

14.2.2 Types of discontinuations 18
14.2.3 Blind review 19
15 Protocol Deviations 19
15.1 Deviations relating to inclusion and exclusion criteria 19
15.2 Deviations relating to treatment and study follow-up visits 19
16 Unblinding 19
17 Effectiveness Evaluations 20
17.1 Data Sets Analysed 20
17.2 Demographic and Other Baseline Characteristics 20
17.3 Compliance with treatment 24
17.4 Analysis of Outcomes 24
17.4.1 Primary outcome: Time to 12-month remission from seizures 25
17.4.2 Secondary Outcome 1: Time to treatment failure 28
17.4.3 Secondary Outcome 2: Time to treatment failure due to inadequate seizure control 30
17.4.4 Secondary Outcome 3: Time to treatment failure due to unacceptable adverse event 31
17.4.5 Secondary Outcome 4: Time to first seizure 31
17.4.6 Secondary Outcome 5: Time to 24-month remission 32
17.4.7 Secondary Outcome 6: Adverse reactions 32
17.4.8 Secondary Outcome 7: Quality of life 33
17.4.9 Secondary Outcome 8: Cost-effectiveness 36
18 Missing data and withdrawals 36
19 Additional analyses 37
19.1 Per-protocol analysis 37
19.2 Sensitivity analysis of primary outcome: different imputation rules for missing data 37
19.3 Sensitivity analysis of primary outcome: exclusion of misdiagnoses 38
19.4 Trial Arm B: Stratification by seizure type 38
19.5 Sensitivity analysis of secondary outcome 1 38
20 Safety Evaluations 39
20.1 Data sets analysed 39
20.2 Presentation of the data 39
20.3 Quality control 39
21 References 40

5 Statement of Compliance

This Statistical Analysis Plan (SAP) provides a detailed and comprehensive description of the pre-planned final analyses for the study “SANAD II”. The planned statistical analyses described within this document are compliant with those specified in brief within the SANAD II protocol v5 22/07/2015. This SAP comprehensively describes the planned final analyses.

This study is carried out in accordance with the World Medical Association Declaration of Helsinki (1964) and the Tokyo (1975), Venice (1983), Hong Kong (1989) and South Africa (1996) amendments and will be conducted in compliance with the protocol, Clinical Trials Research Centre (CTRC) Clinical Trials Unit (CTU) Standard Operating Procedures (SOPs) and EU Directive 2001/20/EC, transposed into UK law as the UK Statutory Instrument 2004 No 1031: Medicines for Human Use (Clinical Trials) Regulations 2004.

These planned analyses will be performed by the trial statistician.

This study is a clinical trial of a medicinal product and is registered on the EudraCT database. The statistical analysis plan has been developed to support the posting of results on the EudraCT system. This is a regulatory requirement which should be fulfilled within 6 months after the end of the study as defined within the clinical trial protocol.
The results of the final analysis described within this statistical analysis plan will be contained within a statistical analysis report. This report will be used as the basis of the primary research publications according to the study publication plan.

All analyses are performed with standard statistical software (SAS version 9.3 or later, and R version 3.3 or later). The finalised analysis datasets, programs and outputs will be archived following Good Clinical Practice guidelines and SOP TM021 Archiving procedure in CTRC.

6 Background and Rationale

The main treatment of epilepsy is anti-epileptic drugs (AEDs). SANAD-II is a phase IV clinical trial with a similar rationale to its predecessor: SANAD-I. While trials are carried out by industry to provide evidence of short term efficacy, there is a need to investigate longer-term effectiveness, quality of life outcomes, and cost-effectiveness of standard and new AEDs currently available.

SANAD-II will measure and compare clinical effectiveness, quality of life, and cost-effectiveness of standard and new AEDs used in the treatment of two mutually exclusive types of epilepsy:

Arm A: Focal onset seizures;

Arm B: Generalised onset seizures or seizures that are difficult to classify.

Arm A patients will be randomised to one of three AEDs: one standard and two new. Arm B patients will be randomised to one of two AEDs: one standard and one new.

See Section 2 of the protocol for further detail.

7 SANAD-II Study Objectives

7.1 Arm A: Patients with focal onset seizure

The aim of this study for patients with this type of epilepsy is (a) to compare the clinical effectiveness and cost-effectiveness of initiating monotherapy with Lamotrigine, Levetiracetam or Zonisamide; and (b) to measure and compare QOL outcomes for each AED and to explore the development and evolution of QOL impairments in patients with newly treated epilepsy.

Primary null hypotheses:

(A1) Levetiracetam is inferior to the standard treatment Lamotrigine with respect to time to 12-month remission. Inferiority margin on hazard ratio scale: 1.329.

(A2) Zonisamide is inferior to the standard treatment Lamotrigine with respect to time to 12-month remission. Inferiority margin on hazard ratio scale: 1.329.

Secondary null hypotheses:

No difference between randomised AEDs with respect to:

(1) Time to treatment failure

(2) Time to treatment failure due to inadequate seizure control

(3) Time to treatment failure due to unacceptable adverse events

(4) Time to first seizure

(5) Time to 24-month remission

(6) Quality of Life

(7) Incremental cost of quality-adjusted years of life (QALY)

7.2 Arm B: Patients with generalised onset seizures or seizures that are difficult to classify

The aim of this study for patients with this type of epilepsy is (a) to compare the clinical effectiveness and cost-effectiveness of initiating monotherapy with Levetiracetam or Valproate; and (b) to measure and compare QOL outcomes for each AED, and to explore the development and evolution of QOL impairments in patients with newly treated epilepsy.

Primary null hypothesis:

(B1) Levetiracetam is inferior to the standard treatment Valproate with respect to time to 12-month remission. Inferiority margin on hazard ratio scale: 1.314.

Secondary null hypotheses:

No difference between randomised AEDs with respect to:

(1) Time to treatment failure

(2) Time to treatment failure due to inadequate seizure control

(3) Time to treatment failure due to unacceptable adverse events

(4) Time to first seizure

(5) Time to 24-month remission

(6) Quality of Life

(7) Incremental cost of quality-adjusted years of life (QALY)

8 Investigational Plan and Study Design

8.1 Overall study design and plan - description

SANAD-II is an open labelled, multi-centre randomised controlled trial in children (aged 5 or older) and adults that are newly diagnosed with epilepsy. It is in effect two randomised controlled trials: arms A and B, run in parallel. Trial arm A is designed for patients with focal onset seizures. Patients are randomised in a 1:1:1 ratio to one of three AEDs – one standard and two new. Trial arm B is designed for patients with generalised onset seizures or seizures that are difficult to classify. Patients are randomised in a 1:1 ratio to one of two AEDs – one standard and one new.

8.2 Treatments studied

Arm A:

Patients will be randomised to one of three possible AEDs:

(1) Lamotrigine (standard treatment)

(2) Levetiracetam (a new treatment)

(3) Zonisamide (a new treatment)

Arm B:

Patients will be randomised to one of two possible AEDs:

(1) Valproate (standard treatment)

(2) Levetiracetam (a new treatment)

See also Sections 7.3.1 and 7.3.2 of the protocol for details of titration and initial maintenance doses for each treatment.

8.3 Treatment compliance

SANAD II is a pragmatic rather than exploratory trial and the intention is to measure outcomes associated with treatment policies, which reflect real life clinical practice in the NHS. There are no formal accountability measures required for the trial, as treatments will be prescribed according to the local medical practices and dispensed by hospital and community pharmacies as they would be normally in clinical practice. See also Section 7.5 of protocol.

8.4 Patient population studied

Patients presenting at participating centres, aged 5 years and over, who have had at least two spontaneous seizures that require antiepileptic drug treatment, and have not previously been treated with antiepileptic drugs.

8.4.1 Inclusion criteria

Patients with the following characteristics will be eligible for inclusion in the trial:

a. Aged 5 years or older
b. Two or more spontaneous seizures that require antiepileptic drug treatment
c. Untreated and not previously treated with antiepileptic drugs, except emergency treatment in the past 2 weeks
d. Antiepileptic drug monotherapy considered the most appropriate option
e. Willing to provide consent (patients parent/legal representative willing to give consent where the patient is aged under 16 years of age or is lacking capacity to consent)

8.4.2 Exclusion criteria

Patients with the following characteristics will be excluded from the trial:

a. Provoked seizures (e.g. alcohol or drug-induced)
b. Acute symptomatic seizures (e.g. within 1 month from acute brain haemorrhage or brain injury or stroke)
c. Currently treated with antiepileptic drugs
d. Progressive neurological disease (e.g. known brain tumour)

8.5 Removal of patients from therapy or assessment

All consenting patients that meet the inclusion criteria and who do not fulfil any of the exclusion criteria will be included in the trial. As SANAD-II is pragmatic, there are no instances of non-compliance that would lead to patients being removed from therapy or assessment.

8.6 Consent process

Potentially eligible patients are invited to participate in the study and provided with a patient information sheet and consent form. The patient (or parent/legally acceptable representative where appropriate) is allowed sufficient time to discuss the trial and decide whether to consent to trial entry (see Section 11.3 of the protocol for further details of consent procedures).

8.7 Blinding

This trial is open-label and there is therefore no blinding.

8.8 Method of assignment to treatment

Participants are randomised using a secure (24-hour) web based randomisation programme controlled centrally by the CTRC. See Section 6.3 of the protocol for details of the randomisation methodology.

8.9 Sequence and duration of all study periods

The study duration of each participant (including follow-up period) is between 2 and 6 years from the date of randomisation. Patients will be followed up as per routine clinical practice and typically at 3, 6, and 12 months and annually thereafter. See Table 5 in Section 8.1 of the protocol for a detailed outline of the assessment schedule.

9 Listing of Outcomes

Both trial arms have the following outcomes measured:

9.1 Primary outcome

Time to 12-month remission from seizures

9.2 Secondary outcomes

Effectiveness outcomes

a. Time to treatment failure;
b. Time to treatment failure due to inadequate seizure control;
c. Time to treatment failure due to unacceptable adverse events;
d. Time to first seizure;
e. Time to 24-month remission
f. Adverse reactions
g. Quality of life (QOL)
h. Cost-effectiveness (Health economic outcomes)
    - Cost of patients’ use of primary and community care services;
    - Cost of patients’ use of secondary care services;
    - Cost of resources triggered by adverse events (where hospitalization is required).

10 Determination of Sample Size

SANAD-II is powered to detect non-inferiority of the new antiepileptic drugs (levetiracetam and zonisamide) compared to standard treatments (lamotrigine or valproate) for the primary outcome time to 12-month remission. Calculations are based on results from the trial SANAD-I for estimates of the probability of a 12-month remission during the first 24 months of follow-up, and assuming an inferiority margin of 10% for this probability for the new treatments. Section 9.4 of the protocol gives full details of the calculation. The total number of patients required for both arms combined is 1510.

Per arm this is broken down to:

Arm A:
With a one-sided alpha of 0.0125, after adjusting for 5% losses to follow-up, 330 patients are required in each of 3 treatment groups. This gives a total of 990 patients.

Arm B:
With a one-sided alpha of 0.025, 260 patients are required in each of two treatment groups allowing for 5% losses to follow-up. This gives a total of 520 patients.

11 Study Framework

This trial has clear objectives to test for non-inferiority of three new treatments over two standard treatments in two separate populations with respect to the primary outcome.

12 Confidence Intervals, p-values and Multiplicity

97.5% 2-sided CI will be used for the primary outcome analysis for arm A (see Section 9.4 of protocol for justification). All other confidence intervals will be calculated at the 95% level, and 2 sided. No formal adjustment will be made for multiple testing. Conclusions drawn from the analysis of all secondary outcomes will be cautionary unless $p<0.001$.

13 Timing and Objectives of Interim and Final Analyses

13.1 Interim monitoring and analyses

Interim monitoring was carried out by an IDSMC, meeting approximately annually. Interim analyses were presented to the IDSMC - planning for these can be found in the document 'SANAD-II SAP IDSMC v1.0'. This included analyses of the primary outcome and five of the secondary outcomes (all using the Haybittle-Peto approach). The IDSMC was asked to give advice on whether the accumulated data from the trial, together with results from other relevant trials, justified continuing recruitment of further patients or further follow-up.

13.2 Final analysis

The trial was planned to finish recruitment in May 2016. However, one arm of the trial was found to have under-recruited at that date. A one-year recruitment extension was granted for Arm A, finishing recruitment in May 2017. This meant that the last follow-up appointment scheduled was in May 2018 for Arm B, and will be in May 2019 for Arm A. The patient records for Arm B will be frozen in February 2019. A final database lock will be completed at the end of 2019, and the final analysis of Arm A and B completed within 6 months of that date.

14 Disposition of Participants

The flow of participants through the study will be displayed in two CONSORT[4] flow diagrams – one for each arm (see Figures 1 and 2 below for the templates that will be used).

14.1 Screening, eligibility and recruitment

SANAD II was not designed to collect any screening data prior to randomisation. As such, there will be no statistics compiled regarding screening or eligibility. Reasons for ineligibility, declining consent and for not being randomised are not recorded, so will not be reported on.

Recruitment graphs will be produced for each arm of the trial, showing recruitment growth over time. Recruitment summary tables will be presented for each trial arm, showing the following for each centre: centre code, hospital name, dates site opened/closed to recruitment, dates of first/last randomisation and total number randomised.

FIGURE 1: TEMPLATE FOR CONSORT DIAGRAM SHOWING FLOW OF RANDOMISED PATIENTS THROUGH THE STUDY IN TRIAL ARM A

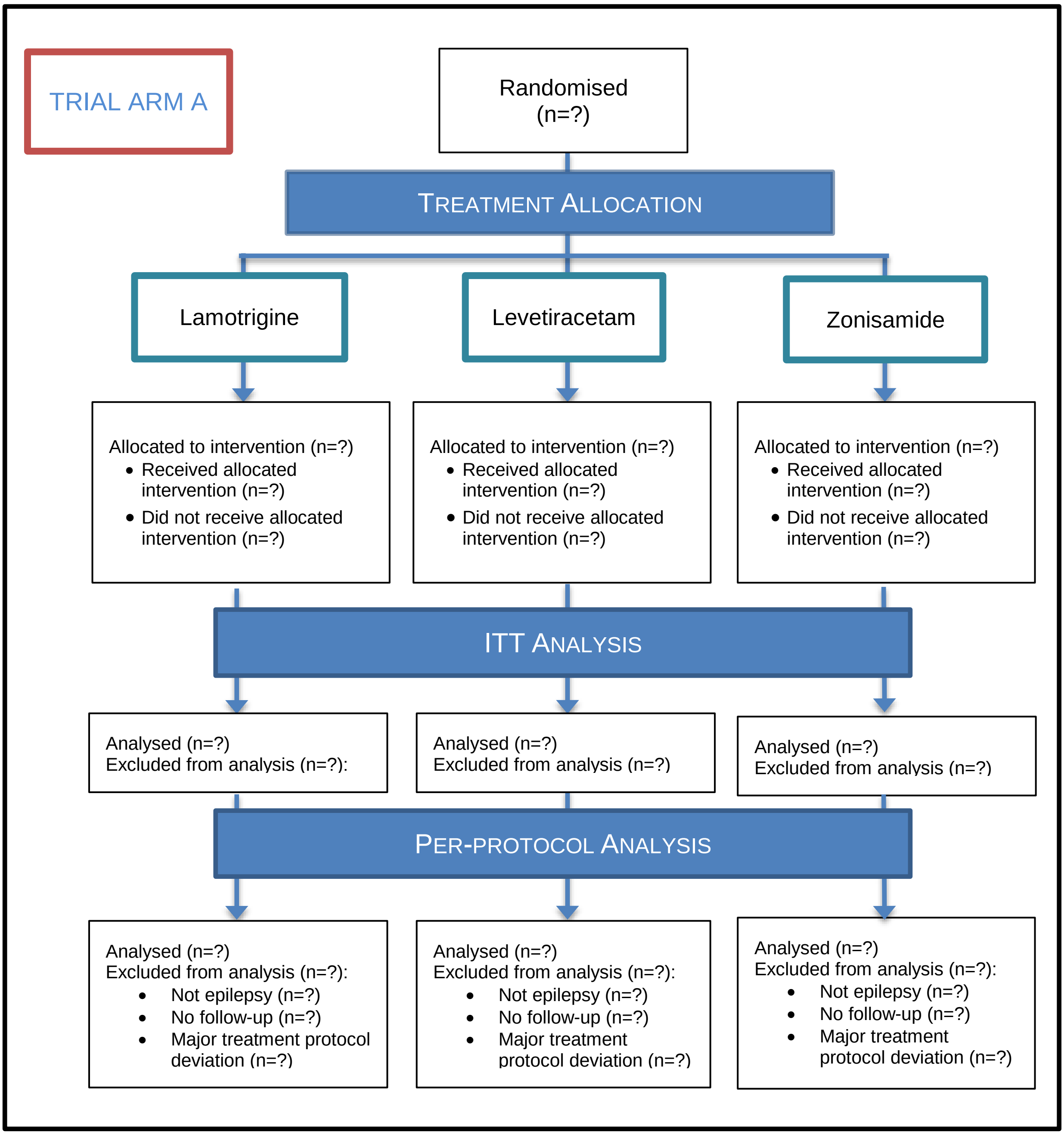

FIGURE 2: TEMPLATE FOR CONSORT DIAGRAM SHOWING FLOW OF RANDOMISED PATIENTS THROUGH THE STUDY IN TRIAL ARM B

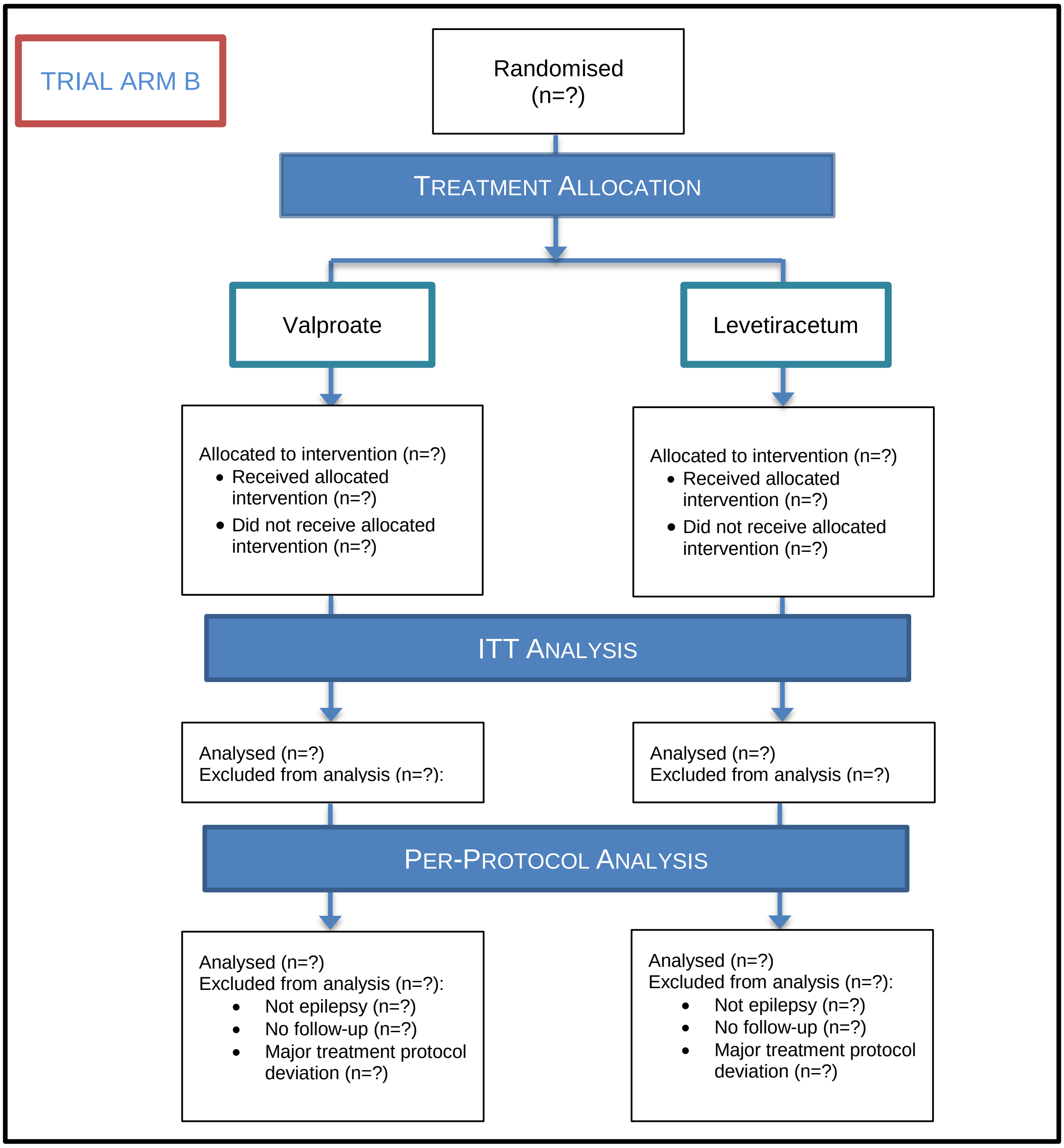

14.2 Post randomisation discontinuations

14.2.1 Completeness of follow-up analysis

A completeness of follow-up statistic will be calculated overall for each arm, and within treatment group using the statistic proposed by Clark *et al* [5]:

$$C = 100 \times \frac{\sum t_i}{\sum t_i^*}$$

where t_i is the length of time (days) patient *i* is actually followed up: from day of randomisation to day that (a) primary outcome occurs; or else either (b) patient completes follow-up (study finishes); or (c) patient is lost to follow-up / withdrawn. t_i^* is the length of time (days) they could potentially have been followed up: from day of randomisation to day that (a) primary outcome occurs; or else either (b) patient completes follow-up (study finishes); or (c) last scheduled follow-up would have taken place if had completed the study (NB $t_i = t_i^*$ if patient *i* in cases (a) and (b)).

Derivation of dates:

Randomisation date – see 17.4.1.1 below.

Primary outcome date - see 17.4.1.1 below.

Date completed follow-up - date of the last recorded follow-up visit (*max*(FLDTPRSD$_i$))

Date withdrawn or lost to follow-up - date patient is withdrawn from the study is recorded in the withdrawal CRF (WDDATEWD). A patient is considered lost to follow-up, if the last recorded follow-up visit is more than a year before the scheduled end of the trial in their arm (Arm A: 31st May 2019; Arm B: 31st May 2018).

The median and range for actual and potential follow-up times will be calculated overall for each arm and within treatment group using the reverse Kaplan-Meier method [6] – i.e. the Kaplan-Meier method with the event indicator reversed so that the outcome of interest becomes the fact of being censored.

A scatter-plot will be produced for each arm, as suggested in Clark *et al* [5], plotting actual follow-up versus time of entry since start of trial. Where the primary outcome occurs, points will be represented by dots; where lost to follow-up, the point will be plotted with a symbol. A solid line will be plotted showing the maximum follow-up possible given entry-date into trial.

14.2.2 Types of discontinuations

The number and percentage that were (i) not-allocated their randomised treatment (denominator: all randomised); (ii) lost to follow-up (denominator: all randomised and received at least one treatment allocation); (iii) discontinued treatment allocation and withdrew from the study (denominator: all randomised and received at least one treatment allocation); and (iv) discontinued treatment allocation and did not withdraw from the study (denominator: all randomised and received at least one treatment allocation) will be tabulated, overall and by randomisation group. Reasons for (i) to (iv) will be categorised as specified below. These will be presented as number and percentage in each category.

(i) Non-allocation of randomised treatment:

- *Reasons to be extracted and listed*

(ii) Loss to follow-up:

- Moved home
- Reason unknown

(iii) Discontinuation of treatment allocation and withdrawal from study due to:

- Inadequate seizure control
- Unacceptable adverse reactions
- Remission of epilepsy
    - > 12 months remission from seizures
    - < 12 months remission from seizures (categorised by patient)
- Diagnosis no longer epilepsy
- Decision of parent/legal representative of child (consent withdrawn)
- Decision of participant (withdrawal of assent)
- Death
    - Related to epilepsy/AED
    - Unrelated to epilepsy/AED
- Other

(iv) Discontinuation of treatment allocation and continuation in study due to:

- Inadequate seizure control
- Unacceptable adverse reactions
- Remission of epilepsy
    - > 12 months remission from seizures
    - < 12 months remission from seizures (categorised by patient)
- Other

Categorisations can be derived from the *Withdrawal* CRF, the *Death* CRF, and page 2 of the *Follow-up* CRF.

14.2.3 Blind review

A blind review of withdrawals from the study will be carried out: information on patients who do not have complete primary outcome data will be provided to reviewers: days to withdrawal (from randomisation), and reasons for withdrawal (see Section 17.4.2.1 below for further details relating to secondary outcomes 1-3).

15 Protocol Deviations

For each trial arm: the overall number of participants experiencing a protocol deviation and the total number of deviations will be reported. Treatment group will be cross-tabulated with type of major and minor deviation. The protocol deviation classifications below are taken from the Monitoring Plan V1.0 (date 12/09/2013).

15.1 Deviations relating to inclusion and exclusion criteria

Major:

- Consent not obtained
- Younger than 5 at recruitment
- Had fewer than 2 seizures prior to screening
- Received previous treatment with AED (i.e. not newly diagnosed at screening)
- Has had a provoked seizure prior to screening
- Has had only acute symptomatic seizures prior to screening.

Minor:

- Has a progressive neurological disease (e.g. known brain tumour)

15.2 Deviations relating to treatment and study follow-up visits

Major:

- Randomised treatment did not start within 7 days of randomisation
- Patient is enrolled in the wrong arm (e.g. has focal epilepsy but has been randomised to a treatment in Arm B of the trial.

16 Unblinding

SANAD-II is an open labelled trial, therefore unblinding is not required.

17 Effectiveness Evaluations

All analyses outlined below will be applied to Arm A and Arm B separately.

17.1 Data Sets Analysed

All primary and secondary outcome analyses (except for safety related outcomes) will be carried out under the intention to treat (ITT) principle. Included will be all randomised participants for whom consent was obtained.
A per-protocol (PP) analysis of the primary outcome will be carried out.

The sample of patients for the PP analysis of primary outcome will be as follows:

(a) Participants who did not receive the drug at all will be excluded
(b) Participants withdrawn from study or drug before achieving a period of remission will be censored at the date of withdrawal
(c) Participants with other AEDs added before achieving a period of remission will be censored at the date of drug addition
(d) Participants with major protocol deviations will be excluded
(e) Participants who were re diagnosed post randomisation as ‘not epilepsy’ will be excluded

Patients to be excluded from analysis populations will be defined in template ST001TEM04: Protocol deviations and data set definitions, will be agreed and approved by the CI and Senior Statistician prior to any release of randomisation codes.

17.2 Demographic and Other Baseline Characteristics

Baseline characteristics of each arm, and then each randomisation group will be summarised using appropriate summary statistics (counts and percentages for categorical variables; mean and SD or median[1] and IQR for numerical variables). Minimum and maximum values will also be presented for numerical variables. Variables to be summarised are given in Tables 1, 2 and 3 below.

[1] Medians and IQR will be used for data that are sufficiently skewed to incur a clinically relevant difference in the mean and median, and/or if the shape of the distribution is not approximately bell-shaped.

TABLE 1: CONTINUOUS BASELINE VARIABLES TO BE REPORTED

Variable	Unit of measure-ment	Precision (decimal places)	CRF where data recorded
Age	Years	1 d.p.	Form 1: Baseline
Weight (if aged ≤ 12 yrs)	Kg	0 d.p.	Form 1: Baseline
QOL (children aged 5-7)	-	1.d.p	Parent baseline questionnaire Parts 1,2 & 3
QOL (children aged 8-11)	-	1 d.p.	Child 8-11 baseline questionnaire
QOL (children aged 12-15)	-	1 d.p.	Child 12-15 baseline questionnaire
QOL (≥ 16 years)	-	1 d.p.	Adult baseline questionnaire

TABLE 2: CATEGORICAL BASELINE VARIABLES TO BE REPORTED

Variable	Categories	CRF where data are recorded
Age-group	5 – 7 yrs<br>8 – 11 yrs<br>12 – 15 yrs<br>16 yrs or older	*Derived from continuous value obtained from*<br>Form 1: Baseline
Age-group[(a)]	Children (2 – 11 yrs)<br>Adolescents (12 – 17 yrs)<br>Adults (18 – 64 yrs)<br>65+	*Derived from continuous value obtained from*<br>Form 1: Baseline
Gender	Male<br>Female	Form 1: Baseline
Learning disability	Yes<br>No	Form 1: Baseline
Neurological deficit	Yes<br>No	Form 1: Baseline
Previous or current neurological disorder (more than one category possible)	Stroke / cerebrovascular<br>Cerebral haemorrhage<br>Intracranial surgery<br>Head injury<br>Meningitis / encephalitis<br>Cortical dysplasia / developmental anomaly<br>Other	Form 1: Baseline
History of symptomatic seizures (more than one category possible)	Febrile convulsions<br>Any other acute symptomatic seizures	Form 1: Baseline

Variable	Categories	CRF where data are recorded
Family history of epilepsy in 1° relative (mother, father or sibling)	Yes<br>No	Form 1: Baseline
Epilepsy syndrome	Focal<br>Generalised<br>Unclassified	Form 1: Baseline
Baseline EEG result (if done)	Normal<br>Abnormality detected:<br>Non-specific<br>Generalised: slow wave activity **with** spiking<br>Generalised: slow wave activity **without** spiking<br>Focal: paroxysmal slow activity **with** spiking<br>Focal: paroxysmal slow activity **without** spiking<br>Other	Form 4: Investigations
Baseline MRI result (if done)	Normal<br>Abnormal:<br>Head injury<br>Tumour<br>Cortical dysplasia<br>Hippocampal sclerosis<br>AVM or other vascular malformation<br>Infarct<br>Haemorrhage<br>Previous infection<br>Other	Form 4: Investigations
Baseline CT scan result (if done)	Normal<br>Abnormal:<br>Head injury<br>Tumour<br>Cortical dysplasia<br>Hippocampal sclerosis<br>AVM or other vascular malformation<br>Infarct<br>Haemorrhage<br>Previous infection<br>Porencephalic cyst<br>Other	Form 4: Investigations

[a] EudraCT defined age-groups

TABLE 3: SEIZURES PRIOR TO RECRUITMENT: VARIABLES TO BE SUMMARISED, SPLIT BY TRIAL ARM

Trial Arm	Variable	CRF where data are recorded
A	No. of seizures of each type: simple partial; complex partial<br>No. of simple or complex seizures with secondary generalisation<br>Days* since first seizure (any type)<br>Days* since most recent seizure (any type)	Form 1: Baseline
B	No. of seizures of each type: myoclonic; typical absence; atypical absence; tonic-clonic (definitely generalised)<br>Days* since first seizure<br>Days* since most recent seizure	Form 1: Baseline
B (unclassified epilepsy)	No. of tonic-clonic (where uncertain whether or not secondary generalised) seizures<br>No. of other types of seizure<br>Days* since first seizure<br>Days* since most recent seizure	Form 1: Baseline

*See note below

Note on calculation of days since first/most recent seizure:

Days since first seizure = Date of randomisation – Date of first seizure

Days since most recent seizure = Date of randomisation – Date of most recent seizure

First and most recent seizure dates are recorded in the Baseline CRF, in separate variables specific to the type of epilepsy. For Arm A, the first seizure date is min(FOSIMDFD, FOCOMDFD, FOTNCDFD), and for Arm B it is min(GENMYDFD, GENTADFD, GENAADFD, GENTCDFD, OTHTCDFD, OTHOTDFD). For Arm A, the most recent seizure date is max(FOSIMDRD, FOCOMDRD, FOTNCDRD), and for Arm B it is max(GENMYDRD, GENTADRD, GENAADRD, GENTCDRD, OTHTCDRD, OTHOTDRD).

Further epilepsy types and sub-types as assessed at baseline will be summarised as number and percentage randomised to each treatment:

Arm A: Focal epilepsy

Type and sub-type of focal epilepsy:

Idiopathic

- Benign childhood epilepsy with centrotemporal spikes
- Childhood epilepsy with occipital paroxysms)

Cryptogenic/symptomatic

- Temporal lobe
- Frontal lobe

- Parietal lobe
- Occipital lobe
- Localisation not specified

Arm B: Generalised epilepsy

Sub-type of idiopathic generalised epilepsy:

Idiopathic

- Childhood absence
- Juvenile absence
- Juvenile myoclonic
- Epilepsy with tonic-clonic seizures on awakening
- Other

Arm B: Other seizures

A line-listing will be presented of other epilepsy syndromes recorded.

17.3 Compliance with treatment

Compliance is not recorded as part of this trial (see protocol, Section 7.5), therefore this section is not applicable.

17.4 Analysis of Outcomes

All analyses outlined below will be applied to Arm A and Arm B separately.

17.4.1 Primary outcome: Time to 12-month remission from seizures

The primary outcome is: Time to 12-month remission from seizures in days.

17.4.1.1 Derivation

Let *E* be the event: patient has had 12 months (365.25 days) remission from seizures. A patient may either: (a) experience the event E; (b) be censored[2] before event E can take place; (c) not experience the event during the course of the study.

Let T_0 be the date of randomisation and T_E be the date that either event E occurs, or the date at which they are censored. Then the primary outcome T is:

$$T = T_E - T_0$$

T is measured in days. T_E is determined using the algorithm in the flowchart below (see Figure 3), from data collected at follow-up visits:

- Date of randomisation (T_0): Randomisation CRF, RANDDTD
- Date of 1st seizure since last visit (T_{S1}): Follow-up CRF, FLFSTSPC
- Date of most recent seizure since last visit (T_{SL}): Follow-up CRF, FLMRCSPS

In using the algorithm, if at any step dates of last and/or most recent seizure are missing or partially complete, imputation will be carried out on a case-by-case basis using all the available information, in the following way: (1) identification of a plausible window for a seizure using all the available information (i.e. what is known about follow-up dates, how many seizures took place since the last follow-up, when the first seizure took place, and when the most recent one took place, working under the assumption that seizures were evenly spaced across between follow-ups); then (2) imputing the date that lies in the middle of the plausible window.

In cases where there is too much uncertainty: i.e. if a date could be any value in a range of more than 3 months; the date will be treated as missing. If missingness means that the primary outcome date is not known, then the patient will be censored at the last time-point where there is certainty that the patient has remained seizure-free.

All participants whose primary outcome is derived on the basis of imputation will be listed in a table and subject to blind review. This will show the derived primary outcome, and all known and unknown data available used in the derivation.

[2] A patient is censored in this time-to-event analysis if they are lost to follow-up or withdraw from the study

FIGURE 3: DECISION FLOWCHART DETERMINING DATE OF EVENT THAT A PATIENT HAS HAD 12 MONTHS REMISSION FROM SEIZURES

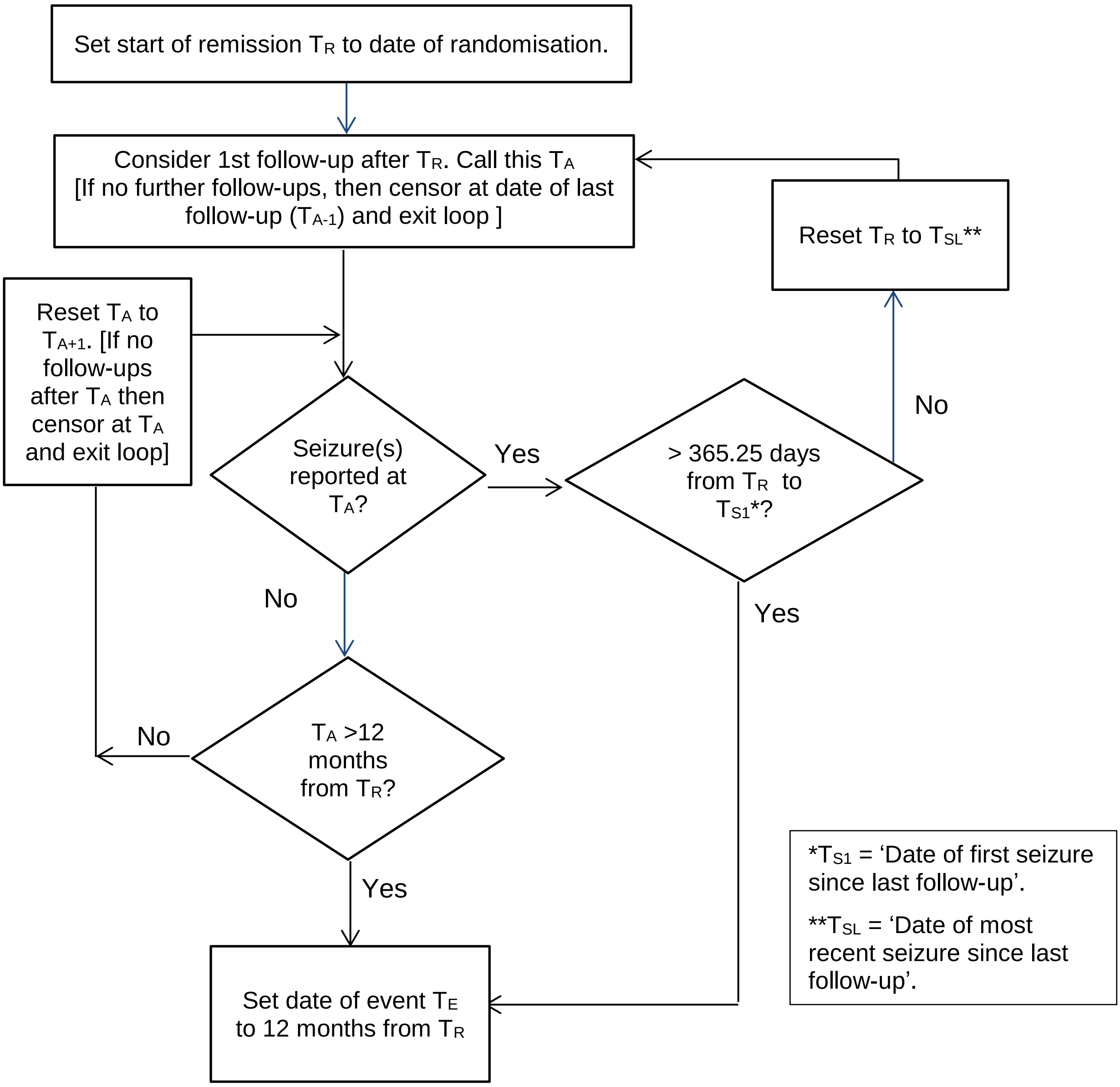

17.4.1.2 Analysis

Separate analyses will be undertaken to test each primary null hypothesis: A1, A2, B1 (see Sections 7.1 and 7.2 above for definitions), each using the following method:

The interval in days *T* will be summarised by a Kaplan-Meier curve for each treatment group. Survival regression models will be explored using two different models:

(i) Including treatment type as the sole explanatory factor;
(ii) Including treatment type **and** covariates.

Cox proportional hazards modelling will be used for Model (i).

Cox proportional hazards modelling with fixed and random effects will be used for Model (ii). Covariates added are the stratification variables for the trial: Fixed effects fitted will be the covariates Gender {M,F} and Number of Seizures prior to Randomisation {2, 3-5, 6+}; centre will be fitted as a random effect.

The assumption of proportional hazards will be investigated by examining Schoenfeld residual plots, and incorporating time-dependent covariates in all models. If residuals are not time-dependent, and the parameter estimate for the time-dependent covariate is not significant at the 5% level, then the assumption of proportional hazards holds; otherwise the extended Cox model with the addition of time-dependent covariates will be used.

All treatment effects will be presented as a hazard ratio with a two-sided 95% confidence interval of standard compared to new. Parameter estimates for covariates will be presented in a results table as hazard ratios with 95% confidence intervals – although the magnitude of these estimates is not of primary interest to this trial. Reference categories will be identified in the results table. For the Arm A analysis, confidence intervals for each of the hazard ratios representing hypotheses A1 and A2, will be concluded as non-inferior if the upper limit is less than 1.329. For the Arm B analysis, the upper limit should be less than 1.314 to conclude non-inferiority in hypothesis B1.

If the upper limit of either hazard ratio confidence interval is less than 1, or the lower limit is great than 1 then the conclusion will be of superiority.

17.4.2 Secondary Outcome 1: Time to treatment failure

Treatment failure (TF) is defined as withdrawal from randomised drug, or addition of a new AED, where the reason is an unacceptable adverse event (UAE) or inadequate seizure control (ISC). TFs, UAEs and ISCs are defined in Table 4 below.

17.4.2.1 Derivation

Let *E* be the event: patient has had a treatment failure during follow-up. A patient will either (a) experience the event; (b) be censored before an event can occur; or (c) not experience the event during the trial.

Let T_0 be the date of randomisation and T_E be either the earliest date that event E occurs, or the date at which a patient is censored. Then the outcome T is:

$$T = T_E - T_0$$

T is measured in days.

TFs and the date at which they occur are captured in the *Follow-up CRF*:

- Patient has withdrawn from randomised drug at date FLDTWBGD, where FLWTHDSC="Yes".
- Patient has a new AED added to their treatment regimen at date FLBVDD, where FLBVNADC="Yes" or at date FLDPRSD where FLTVNADC="Yes".

Table 4 defines whether TFs are considered an event for this outcome. Unacceptable adverse event (UAE) and inadequate seizure control (ISC) are captured in the *Follow-up CRF*:

- UAE: FLPRSWDC=2
- ISC: FLPRSWDC=1 **or** FLBVPRC=1 **or** FLTVPRC=1

If the first TF occurs for another reason (see Table 4 for pre-defined rules indicating whether the TF is an event) the patient is censored at the date of the TF. These categorisations will be carried out during blind review (by an independent statistician blind to data and also the CI). Note that a patient is censored at the date of withdrawal from the study if the reason for withdrawal is not classified as an event; at their last follow-up date if lost to follow-up; or at the date of death if for a reason unrelated to their treatment or epilepsy. Dates due to withdrawal and reasons for withdrawal can be found in the *Withdrawal CRF* and the *Death CRF*. It is generally not possible, but in a very few cases more than one reason category may be applicable for a TF. If at least one of the reasons is a UAE or an ISC, then the TF is an event.

If the exact date of an event is unobtainable, the imputation principles outlined in Section 17.4.1.1 above will be applied.

TABLE 4: METHOD OF CATEGORISING WHETHER TREATMENT FAILURE IS AN EVENT[1]

Reason for withdrawal from randomised drug / addition of a new AED	Categorised as event or censored in 'time to treatment failure'	ISC/UAE
Inadequate seizure control	Event	ISC
Unacceptable adverse events	Event	UAE
Remission of epilepsy categorised by clinician (regardless of length in remission)	Censored	-
Remission of epilepsy categorised by patient (MORE than 12 months remission from seizures)	Censored	-
Remission of epilepsy categorised by patient[2] (LESS than 12 months remission from seizures)	Event	UAE
Diagnosis no longer epilepsy	Censored	-
Study withdrawal - Consent withdrawn[3]	Censored	-
Death (unrelated to epilepsy/AED)[4]	Censored	-
Death (related to epilepsy/AED)[4]	Event	Could be ISC or UAE or neither
Moved from area	Censored	-
Patient non-compliant / did not wish to continue[5]	Event	Could be ISC or UAE or neither
Perceived adverse effect e.g. pregnant or planning pregnancy	Event	UAE

[1] The first two columns of this classification table were also used for SANAD I
[2] Patients decision to withdraw before 12 months freedom from seizures is likely to be highly influenced by side effects or perception of side effects
[3] Study withdrawals are automatically checked to ensure that the patient wants to withdraw from study rather than from drug only
[4] Relatedness recorded in Death CRF
[5] Further information is to be sought if patient withdraws from drug due to "non-compliance" as the underlying reason could be unacceptable adverse events, inadequate seizure control **OR** remission of epilepsy. If further information is unavailable sensitivity analyses will be performed firstly coding non-compliance as event then secondly as a censored observation.

17.4.2.2 Analysis

Time to treatment failure will be compared for each of the three pairwise treatment comparisons described in the primary analysis. The method of model fitting will be the same as described in 17.4.1.2 above. Parameter estimates for covariates will be presented in a results table as hazard ratios with 95% confidence intervals – although the magnitude of these estimates is not of interest to this trial. Reference categories will be identified in the results table. For the Arm A analysis, the confidence interval for each of the hazard ratios representing the comparison of new treatment with standard, will be concluded as consistent with the null hypothesis if the interval includes 1. The same method will be used for testing the null hypothesis comparing new and standard treatments in Arm B.

17.4.3 Secondary Outcome 2: Time to treatment failure due to inadequate seizure control

17.4.3.1 Derivation

Treatment failure due to inadequate seizure control is defined as a treatment failure where the reason given for either withdrawal or addition of another AED is given as 'Inadequate seizure control' (ISC). Derivation of the event and date it occurs can be found in 17.4.2.1 above. Time to treatment failure due to inadequate seizure control is the time in days between the date of the treatment failure and randomisation. Participants that have not experienced this event will be censored at the time of their last follow-up appointment or the date of withdrawal if applicable.

If the exact date of an event is unobtainable, the imputation principles outlined in Section 17.4.1.1 above will be applied. All participants whose time-to-treatment failure due to ISC is derived on the basis of imputation will be listed in a table. This will show the derived date, and all known and unknown data available used in the derivation. This table will be subject to blind review.

17.4.3.2 Analysis

Analysis of time to treatment failure due to inadequate seizure control will be carried out incorporating the analysis of Secondary Outcome 3: time to treatment failure due to unacceptable adverse event. This will be carried out using a competing risks analysis, using the Fine and Gray model [7]. The 'cmprsk' package in software package R, version 3.3.1 or later will be used for this analysis. Cumulative incidence plots will be presented for each randomisation group – one plot per trial arm. Hazard ratios will be calculated and presented with 95% confidence intervals, for each treatment comparison and each risk.

17.4.4 Secondary Outcome 3: Time to treatment failure due to unacceptable adverse event

17.4.4.1 Derivation

Treatment failure due to unacceptable adverse reaction occurring is defined as a treatment failure where the reason given for either withdrawal or addition of another AED is given as 'Unacceptable adverse reaction'. Derivation of the event and date it occurs can be found in 17.4.2.1 above. Time to treatment failure due to unacceptable adverse reaction occurring is the time in days between the date of the treatment failure and randomisation. Participants that have not experienced this event will be censored at the time of their last follow-up appointment.

If the exact date of an event is unobtainable, the imputation principles outlined in Section 17.4.1.1 above will be applied. All participants whose time-to-treatment failure due to UAE is derived on the basis of imputation will be listed in a table. This will show the derived date, and all known and unknown data available used in the derivation. This table will be subject to blind review.

17.4.4.2 Analysis

The analysis of this outcome is included in the analysis of Secondary Outcome 2 (see 17.4.3.2 above.)

17.4.5 Secondary Outcome 4: Time to first seizure

17.4.5.1 Derivation

The first seizure is defined as the first occurrence of a seizure of any type following randomisation. The date of first seizure since last follow-up is recorded in the *Follow-up* CRF at each follow-up visit, by the variable FLFSTSPC. Participants that have not experienced any seizure will be censored at the date of their last follow-up appointment, or if applicable, the date of withdrawal.

If the exact date of an event is unobtainable, the imputation principles outlined in Section 17.4.1.1 above will be applied. All participants whose date of first seizure is derived on the basis of imputation will be listed in a table. This will show the derived date, and all known and unknown data available used in the derivation. This table will be subject to blind review.

17.4.5.2 Analysis

Time to first seizure will be compared for each of the three pairwise treatment comparisons described in the primary analysis. The method of model fitting will be the same as described in 17.4.1.2 above. Parameter estimates for covariates will be presented in a results table as

hazard ratios with 95% confidence intervals – although the magnitude of these estimates is not of interest to this trial. Reference categories will be identified in the results table. For the Arm A analysis, the confidence interval for each of the hazard ratios representing the comparison of new treatment with standard, will be concluded as consistent with the null hypothesis if the interval includes 1. The same method will be used for testing the null hypothesis comparing new and standard treatments in Arm B.

17.4.6 Secondary Outcome 5: Time to 24-month remission

17.4.6.1 Derivation

Let *E* be the event: patient has had 24 months remission from seizures. Let T_0 be the date of randomisation and T_E be the date that event E occurs. Then the outcome T is:

$$T = T_E - T_0$$

T is measured in days. T_E is determined using the algorithm in the flowchart above (see Figure 3 above), replacing the text '12 months' with '24 months'. Data informing this are collected at follow-up visits from the same variables described in 17.4.1.1. If the exact date of an event is unobtainable, the imputation principles outlined in Section 17.4.1.1 above will be applied. All participants whose time-to-treatment 24-month remission is derived on the basis of imputation will be listed in a table. This will show the derived dates, and all known and unknown data available used in the derivation. This table will be subject to blind review.

17.4.6.2 Analysis

Time to 24-month remission will be compared for each of the three pairwise treatment comparisons described in the primary analysis. The methods will be the same as described in 17.4.1.2, but the null hypothesis is that there is no association between treatment type and the time to 24-month remission.

17.4.7 Secondary Outcome 6: Adverse reactions

Detailed presentation of adverse reactions is provided in the safety analysis (see Section 20 below).

17.4.8 Secondary Outcome 7: Quality of life

17.4.8.1 Derivation

Quality of life (QOL) is measured by questionnaire at each follow-up appointment. There are three self-assessed questionnaires: 8 to 11 year-olds, 12 to 15 year-olds, and adults (16 years or over); and one parent/carer questionnaire relating to children aged between 5-15. Each questionnaire is a combination of age-appropriate questions taken from six validated questionnaire source tools: Kiddy-KINDL, Kid-KINDL, Kiddo-KINDL, QUOLIE-AD, NEWQOL and the Impact of Epilepsy Scale. The source tools used for each age-group are defined in Table 6 of the protocol.

Table 5 below lists all possible outcomes derivable from each questionnaire, and the question(s) that contribute to each outcome. Each outcome is generally a sum of the scores for each question, with some scores being reversed to ensure that higher scores all imply higher scoring of the outcome. Scoring of responses, and how to combine responses to derive outcomes is defined in the manuals for each source tool [8]-[11].

TABLE 5: QUALITY OF LIFE OUTCOMES BY QUESTIONNAIRE TYPE

Outcome	Parent/Carer (where child is aged 5-15)	Self-report		
		Child (8-11)	Child (12-15)	Adult (>15)
**Seizure worry**	-	-	-	Q1
**Adverse drug effects**	-	-	-	Q4: 19 sub-questions
**Anxiety**	-	-	-	Q5: sub-questions a,c,e,g,I,k,m
**Depression**	-	-	-	Q5: from sub-questions b,d,f,h,j,l,n
**Mastery**	-	-	-	Q6: 7 sub-questions
**Stigma**	-	-	-	Q7: 3 sub-questions
**Impact of Epilepsy Scale**	-	-	Q25-39	Q8: 12 sub-questions
**Attitude to epilepsy**	-	Q25-27	-	-
**QOL: Physical**	Q1: 4 sub-questions			-
**QOL: Emotional**	Q2: 4 sub-questions			-
**QOL: Self-esteem**	Q3: 4 sub-questions			
**QOL: Family**	Q4: 4 sub-questions			
**QOL: Social**	Q5: 4 sub-questions			-
**QOL: School**	Q6: 4 sub-questions			
**Overall QOL**	-	-	-	Q9

17.4.8.2 Analysis

Completeness of data

Completeness of questionnaire data return will be tabulated. For each age-group, and each time-point the following statistics will be presented: the total number of potential respondents, the actual number of respondents, and the response rate (as a percentage of actual out of potential). NB Number of potential respondents is the total number within the questionnaire age-group, still in the study at the time-point.

Summary statistics:

For each outcome measure, appropriate summary statistics by age-group, scheduled time-point, whether self-assessed or by proxy, split by randomisation group will be calculated: number and percentage of respondents, mean, standard deviation, median, interquartile range, min and max. Mean (SD) score will also be plotted on a graph: one graph per outcome measure and age-group; one line plotted per randomisation group. Excluded data: if the date of completion of a questionnaire is not sufficiently close to the target follow-up time-point (more than +/-2 weeks different for the 3-month follow-up and +/-1 month different for the yearly follow-up), then questionnaire responses will not be included in this analysis.

Longitudinal analysis:

For each arm, each population (child/adult/parent-carer), and for each outcome applicable within that population:

A repeated measures random effects model will be fitted. The baseline measurement of the outcome will be fitted as a covariate along with treatment group, and time-point as a continuous covariate. A treatment-time interaction may be fitted, but dropped if this term does not significantly improve the fit of the model. The recommended covariance structures are a spatial-power covariance structure for the repeated measures, and an unstructured covariance structure for the random effect. The covariance structure for the repeated measures may be changed to an alternative spatial structure in the event that the model does not converge.

The following will be reported for each model: number of observations included in the model, number of participants included within each treatment group, mean (SE) for each treatment group at each key follow-up time-point; mean (95% CI) pairwise difference between treatment groups at 2 years in change from baseline; and a p-value of the treatment effect if there is no interaction present.

Notes:

1. Longitudinal analysis of an outcome for a participant is only possible where a baseline measurement exists. E.g. children that become adults during the study will only be included in models of child-specific outcomes – and only time-points for which they remain a child will be relevant to the analysis.
2. Longitudinal analysis of ‘Attitude to epilepsy’ in 8-11 year olds is restricted to children that begin the trial in that age-group and follow-up measurements where they stay in the same age-group.
3. Longitudinal analysis of ‘Impact of epilepsy’ in 12-15 year olds is restricted to children that begin the trial in that age-group and follow-up measurements where they stay in the same age-group.
4. The sample size for some of these analyses may be too small to enable a model to be fit and for inference to be reported.
5. As time fitted is a continuous covariate in these models, all data may be included regardless of whether at a target follow-up time.
6. Baseline and follow-up for participants aged 5-8 will be derived from parent/carer questionnaires.

17.4.9 Secondary Outcome 8: Cost-effectiveness

A separate Health Economics Analysis Plan (HEAP) will be developed by the Health Economics team working on this trial.

18 Missing data and withdrawals

All analyses outlined below will be applied to Arm A and Arm B separately.

Line listings of patient withdrawals from follow-up with reasons will be presented, overall and split by trial arm and randomisation group. Reasons for dropouts will be recorded by randomisation group. The protocol states that any missing data on CRFs must be explained – either as ‘Not done’, ‘Not applicable’, or ‘Not recorded. Missing data within CRFs that is not confirmed with one of these terms will be queried.

The primary outcome is time-to-event. Patients who drop out of the study may have experienced a 12-month remission prior to drop-out – in which case the primary outcome can be measured – otherwise they will be censored at the time of their last follow-up appointment. The same is true of all time-to-event secondary outcomes. Methodology for handling missing

seizure dates is given in Section 17.4.1.1, Table 4. Longitudinal study of quality of life will use all available data up to participants' last follow-up.

19 Additional analyses

The following are all re-analyses of the primary outcome under different conditions:

- Per-protocol
- Missing data imputation in derivation of primary outcome – under different assumptions
- Excluding misdiagnoses
- Stratified by seizure type

One further sensitivity analysis is also required for secondary outcome 1: time to treatment failure.

19.1 Per-protocol analysis

A per-protocol analysis of the primary outcome will be undertaken to assess the robustness of ITT analyses and to further inform decisions regarding non-inferiority (see Section 17.1 for definition of who is included in the PP analysis set). The clinical and statistical issues of informative censoring for the PP analysis of the remission outcome has been identified: if seizure-related withdrawals (withdrawals from study or drug prior to achievement of a period of remission) or drug additions (additional AED added prior to achievement of a period of remission) are censored at the date of withdrawal (date withdrawal started) or addition, the underlying assumption that time to achieve remission for an individual is independent of any mechanism which causes that individual's time to be censored at some time is violated. For this reason, the preferred analysis will be carried out using a competing risks analysis, using the Fine and Gray model [7]. The 'cmprsk' package in software package R, version 3.3.1 or later will be used for this analysis. Cumulative incidence plots will be presented for each randomisation group – one plot per trial arm. Hazard ratios will be calculated and presented with 95% confidence intervals, for each treatment comparison and each risk. The analysis outlined in Section 17.4.1.2 will also be repeated on the per-protocol dataset for consistency.

19.2 Sensitivity analysis of primary outcome: different imputation rules for missing data

A sensitivity analysis of the primary outcome, specific to participants whose primary outcome is dependent on imputation of missing data. The list of participants for whom data imputation was used to derive the primary outcome will examined. Where imputation leads to a remission period of more than 1 year, the date will be re-imputed such that it leads to a failure of the

primary outcome, and a re-derivation of the outcome using all available data during the rest of follow-up; where imputation leads to a failure of the primary outcome, the date will be re-imputed such that it leads to a remission period of 1 year from the date of the last seizure.

19.3 Sensitivity analysis of primary outcome: exclusion of misdiagnoses

A sensitivity analysis of the primary outcome, specific to misdiagnoses will be carried out. The dataset analysed will be all participants randomised that were not subsequently found to be 'not epilepsy'. Misdiagnosis is recorded in the Withdrawal CRF, variable WDNOTEPC.

19.4 Trial Arm B: Stratification by seizure type

A stratification variable will be added to the ITT primary outcome analysis of trial arm B: seizure type (generalised / unclassified). For patients that are recorded at baseline with a history of both generalised and unclassified seizures, the CI will decide on a single primary classification based on the available medical history (this is expected to be rare). For the Kaplan Meier curve, the plot will be split by treatment group and seizure type. Seizure type is defined on page 5 of the Baseline CRF, variable EPCLASSC.

19.5 Sensitivity analysis of secondary outcome 1

Table 4 defines whether a treatment failure is an event. A sensitivity analysis of 'time-to-treatment-failure' will be carried out, categorising the treatment failure: "Remission of epilepsy categorised by patient (LESS than 12 months remission from seizures)" as **censored** instead of as an event.

20 Safety Evaluations

20.1 Data sets analysed

The safety analysis data set will contain all participants that are randomised and commenced trial treatment.

20.2 Presentation of the data

Descriptions of adverse reactions/serious adverse reactions[3] are coded using the MedDRA dictionary to the most appropriate lower level term (LLT). If the text on the form does not match a MedDRA LLT, the chief investigator will decide on the appropriate LLT. The LLTs are entered in MACRO by the data manager. The LLTs are recoded into preferred terms (PT) to combine differing terms for the same reaction, using the MedDRA lookup tables. Each preferred term is also categorised into the higher level system organ classification (SOC), using the MedDRA lookup tables. This process is carried out by the statistician.

Tables of overall frequencies of adverse reactions (number of events and number of patients) will be presented for each treatment group. The adverse reactions will be reported both at SOC and PT level.

The above tables will be repeated for only those adverse reactions categorised as severe.

Total number of SARs and SUSARs will be presented for each treatment group, along with line listings for each SAR or SUSAR, including SAR description, AEDs taken, seriousness, severity, expectedness, relationship, whether treatment was withdrawn, and outcome.

20.3 Quality control

To ensure quality control, an independent statistician will follow this SAP to independently program the primary outcome analysis and the safety analysis from the raw data. Any discrepancies found will be discussed with the trial statistician to resolve. No programming will be shared or shown between the statisticians. The independent statistician will also check the report against their output obtained from the statistical software.

[3] Adverse events are not recorded in this trial – only adverse reactions

21 References

1. International Conference on Harmonisation. Topic E3 Structure and content of clinical study reports.(CPMP/ICH/137/95). 1995
2. International Conference on Harmonisation. Topic E9 Statistical Principles for Clinical Trials (CPMP/ICH/363/96). 1998.
3. Guidance on Statistical Analysis Plans.
4. Piaggio G, Elbourne DR, Pocock SJ, Evans SJW, Altman DG, for the CONSORT Group. Reporting of noninferiority and equivalence randomized trials. Extension of the CONSORT 2010 statement. JAMA. 2012; 308(24): 2594-2604. doi:10.1001/jama.2012.87802. PMID: 23268518
5. Clark TG, Altman DG, De Stavola BL. Quantification of the completeness of follow-up. Lancet. 2002 Apr 13;359(9314):1309-10. PubMed PMID: 11965278
6. Schemper M, Smith TL: A note on quantifying follow-up in studies of failure time. Controlled Clinical Trials. 1996, 17(4):343-346
7. Fine, J. P., & Gray, R.J. 1999. A proportional hazards model for the subdistribution of a competing risk. Journal of the American Statistical Association, 94(446), 496{509.
8. Jacoby A, Baker G, Smith D, Dewey M, Chadwick D. Measuring the impact of epilepsy: the development of a novel scale. Epilepsy Res. [Research Support, Non-U.S.Gov't]. 1993 Sep;16(1):83-8.
9. Abetz, L., Jacoby, A., Baker, G. A., & McNulty, P. (2000). Patient-Based Assessments of Quality of Life in Newly Diagnosed Epilepsy Patients: Validation of the NEWQOL. EPILEPSIA, (9), 1119.
10. https://www.kindl.org/english/manual/
11. Cramer JA, Westbrook LE, Devinsky O, Perrine K, Glassman MB, Camfield C. Development of the Quality of Life in Epilepsy Inventory for Adolescents: the QOLIE-AD-48. Epilepsia. [Multicenter Study]. 1999 Aug;40(8):1114-21.
