# Supplementary material for: The SANAD II study of the effectiveness and cost-effectiveness of valproate versus levetiracetam for newly diagnosed generalised and unclassifiable epilepsy: an open-label, non-inferiority, multicentre, phase 4, randomised controlled trial
Source: Lancet. 2021 Apr 10;397(10282):1375–86. doi: 10.1016/S0140-6736(21)00246-4 (PMC8047813; doi:10.1016/S0140-6736(21)00246-4)
Supplement: Supplementary appendix 4 [file mmc4.pdf]

THE LANCET

Supplementary appendix

This appendix formed part of the original submission and has been peer reviewed. We post it as supplied by the authors.

Supplement to: Marson A, Burnside G, Appleton R, et al. The SANAD II study of the effectiveness and cost-effectiveness of valproate versus levetiracetam for newly diagnosed generalised and unclassifiable epilepsy: an open-label, non-inferiority, multicentre, phase 4, randomised controlled trial. *Lancet* 2021; **397:** 1375–86.

Methods

Overview

The economic analysis was conducted from the perspective of the National Health Service (NHS) and Personal Social Services (PSS) in the UK. The primary economic analysis compared the costs and consequences of each antiepileptic drug over the first 24 months post randomisation. An analysis at an extended 48-month time horizon was planned for those participants followed up for 4 years or more.

The within-trial economic analysis was performed using individual, patient-level data from the SANAD-II trial. A cost-utility analysis was conducted to estimate incremental cost-effectiveness ratios, expressed as costs per quality-adjusted life years (QALY) gained.

The health economic analysis was carried out in Stata IC version 13 (StataCorp LLc, College Station, TX), and reported according to the CHEERS statement.[1]

Data sources

Resource-use

Participants' use of resources was considered in four broad categories: i) resource-use associated with secondary care (inpatient, outpatient, accident and emergency), ii) other healthcare and social services resource-use (primary care, community services), iii) use of anti-seizure medication, and iv) use of other medications.

The measurement of resource-use was based on complementary approaches, using data collected as part of the trial, and as part of routine care. Resource-use postal questionnaires, completed by the parent or carer for participants under the age of 16, included a modified Client Service Receipt Inventory (CSRI) based on that from the SANAD trial.[2-4] This was used to collect information on participants' use of health service resources, personal social services and medicines. Questions pertained to contacts with health professionals at the GP surgery, in the hospital and in the community, the use of emergency services, and any tests or investigations which participants may have had. The questionnaires were initially administered at 3, 6, 12 months and annually thereafter (up to 60 months); however, from Protocol Version 7 onwards, this questionnaire was also provided during outpatient visits to aid completeness. Questionnaires completed following visits were matched to respective time points for analysis.

In all cases, participants were asked to report their primary and secondary care and social services resource-use for the 3-month period prior to completing the questionnaire, and to report their medicines use over a 4-week period prior to completing the questionnaire due to the additional complexity in the recall. The self-report questionnaires additionally contained free-text sections which allowed participants to record any resource-use which would not otherwise be captured by the questionnaire. During analysis these were assessed for duplication against those resources captured by the questionnaire, and any relevant, non-duplicated resources were extracted. Prior to Protocol Version 7 the questionnaire included additional questions relating to a broader perspective,[5] however these were removed in order to shorten the questionnaire, improve completion rates and to prioritise the NHS and PSS perspective, consistent with the NICE guidance for technology appraisal.[6]

Self-report data were therefore available for months 0-3, 3-6, 9-12, and 21-24. Self-reported resource-use for year 1 was estimated by multiplying the resource-use from months 9-12 by two, and adding the resource-use reported for months 0-3 and 3-6. Self-reported resource-use for year 2 was estimated by multiplying resource-use for months 21-24 by four, and similarly for years 3, 4 and

5. Participants' use of concomitant medicines was multiplied by three (due to the shorter, 4-week recall period), before estimation following the same method.

Anti-seizure medications and their respective doses were recorded directly within case report forms.

Routine Hospital Episode Statistics (HES) were the primary source of data on participants' use of secondary care resources over the trial period. HES data were obtained from NHS Digital (for patients in England)[7] and, from the Secure Anonymised Information Linkage (SAIL) databank (for patients in Wales).[8] HES data were not obtained for patients in Scotland or Northern Ireland. HES provided Health Resource Group (HRG) data on the type of care patients receive at a ward level, outpatient visits and Accident and emergency admissions. HES data were used as the source for baseline resource-use and costs, based on the 6 months prior to randomisation. Adjustments were made where hospital episodes overlapped with randomisation date, in order to apportion the resource-use to the periods prior to, and subsequent to, randomisation.

All resource-use was measured irrespective of whether they were epilepsy related or otherwise.[9]

Unit costs

Resource-use was valued in monetary terms (£ sterling) using sources of national unit costs.[10-13]

For data pertaining to participants from Wales an initial mapping step was performed using the Welsh NHS Data dictionary.[14] Subsequently, HRG codes were obtained from the HES data using the NHS Digital costing grouper.[15] Unit costs were allocated based on the latest available National Schedule.[10]

Unit costs for primary care and community care were taken from the compendium of Unit Costs of Health and Social Care.[11] Unit costs and their sources relating to items within the self-report questionnaire, are presented in Table 1. Unit costs relating to the most commonly reported HRGs are presented in Table 2.

Total costs for resource-use were calculated by multiplying the unit cost per item by the recorded number of times that each resource was used.

Table 1: Unit costs relating to self-reported resource use

Item of resource	Unit cost (child)	Assumption	Source
GP consultation at GP surgery	£39	9.22 minutes	11
Nurse consultation at GP surgery	£10.85	15.5 minutes	11,16
GP home visit	£99.45	11.4 minutes, 12 minutes travel	11,16
Nurse home visit	£40	N02AF	10
Dr at hospital	£185 (£203)	Adult: Service 400<br>Child: Service 223	10
Nurse at hospital	£29.19	15.5 minutes	11
Hospital overnight	£589	Non-elective stay	10
Ambulance	£257	ASS02	10
A&E visit	£192.18	(T01A, T01NA)*	10
Blood test	£3	DAPS05	10
Urine test	£2	DAPS	10
Ultrasound	£54.82	(RD40Z, RD41Z, RD42Z, RD43Z)*	10

X-Ray	£31	DAPF	[10]
CT scan	£88.53<br>(£99.74)	Adult: (RD20A, RD21A)*<br>Child: (RD20B, RD21B)*	[10]
MRI scan	£138.24<br>(£141.87)	Adult: (RD01A, RD02A)*<br>Child: (RD01B, RD02B)*	[10]
EEG	£199<br>(£340)	Adult: AA33C<br>Child: AA33D	[10]
Health visitor	£72	N03G	[10]
Social worker	£50<br>(£51)	1-hour visit	[11]
Occupational therapist	£83<br>(£141)	Adult: A06A1<br>Child: A06C1	[10]
Psychologist	£199	Service 656	[10]
Counsellor	£45<br>(£94)	1-hour visit	[11]
Physiotherapist	£63<br>(£101)	Adult: A08A1<br>Child: A08C1	[10]
Resources identified from free text			
Telephone consultation (GP)	£15.52		[11]
GP out of hours	£72.97	Inflated to 2018/19	[17]
MMR	£7.64	In addition to nurse appointment	[12]
Pharmacist	£11	Band 6, 15 mins	[11]
Repeat prescription	£7.30		[11]
Stool test	£2	DAPS	[10]
MRSA swab / Saliva test	£8	DAPS07	[10]
Psychiatrist	£226<br>(£227)	Adult: Service 713<br>Child: Service 711	[10]
Support worker	£24		[11]
Speech therapist	£107<br>(£100)	Adult: A13A1<br>Child: A13C1	[10]
Dietitian	£90	A03	[10]
Podiatrist	£43	A09A	[10]
Podiatrist minor surgery	£86	A09B	[10]
Midwife	£58	N01A	[10]
Hearing test	£101<br>(£89)	Adult: CA37A<br>Child: CA37B	[10]
Optician	£76	Service 662	[10]
NHS glasses	£39.10	Voucher A	[18]
Dentist	£98	M01B	[10]
Orthodontist	£121	Service 143	[10]
CAMHS	(£221)	CAMHSCC	[10]
School nurse / SENCO	(£68)	N05CO	[10]
Mammogram	£57.37	Inflated to 2018/19	[19]
Cervical smear	£39.76	Inflated to 2018/19	[20]
NHS Direct	£13.02	Inflated to 2018/19	[21]
Anticoagulant Service	£37	Service 324	[10]
Radiofrequency for pain management	£699	AB15Z	[10]
Radiotherapy	£182	SC31Z	[10]
ECG	£72.57	Adult: RD51A	[10]

	(£53.58)	Child: RD51B	
Video telemetry / Long term EEG monitoring	£491	AA81Z	[10]
Cerebral angiogram/ Contrast fluoroscopy	£170	RD31Z	[10]
Spinal fluid test	£617<br>(£882)	Adult: HC72A<br>Child: HC72B	[10]
Cystoscopy	£250<br>(£849)	Adult: LB72A<br>Child: LB72B	[10]
Colonoscopy	£520	FE32Z	[10]
Sigmoidoscopy	£386	FE35Z	[10]
Endoscopy	£454	FE22Z	[10]
Dexa scan	£71.92	RD50Z	[10]
PET scan	£506<br>(£389)	Adult: RN01A<br>Child: RN01B	[10]
Peak flow test	£152	DZ45Z	[10]
Field Exercise Test	£55	DZ32Z	[10]
Cataract operation	£915	BZ34C	[10]
Orthotics	£124	Service 658	[10]
Intermediate sinus procedures	£2344	CA28Z	[10]
Insertion of grommets	£998	CA35B	[10]
Arm fracture & CC	£1417	HE51G	[10]
Rib fracture	£1025	HE71D	[10]
Hand fracture	£384	HE41D	[10]
Minor dental procedures <19	£153	CD03B	[10]
Tooth extraction 18 & under	£491	CD07B	[10]
Minor skin procedures	£215<br>(£288)	Adult: JC43C<br>Child: JC43D	[10]
Diabetic retinopathy screen	£108	BZ88A	[10]
Nasal polypectomy	£1715	CA14Z	[10]
Skin biopsy external nose	£461	CA16Z	[10]
Percutaneous biopsy	£1491	YH32A	[10]
Liver biopsy	£671	YG11A	[10]
Biopsy of prostate	£504	LB76Z	[10]
Sleep apnoea test	£309	DZ50Z	[10]
Pelvis fracture (hip fracture)	£2117	HE11H	[10]
Vaginal tape operation for urinary incontinence	£2020	LB51B	[10]
Minor foot operation	£832<br>(£580)	Adult: HN35A<br>Child: HN35B	[10]
Hernia repair	£2651	FF60D	[10]
Hysterectomy	£3515	MA08B	[10]
Triple heart bypass	£10199	ED28B	[10]
Hip replacement	£6057	HN12F	[10]
Pacemaker fitted	£1085	EY08E	[10]
Implantation of loop recorder	£1270	EY12B	[10]
Removal of loop recorder	£693	EY13Z	[10]
Cholecystectomy (gall bladder removal)	£2861	GA10K	[10]
Knee replacement	£5699	HN22E	[10]
Reconstructive surgery (chest clinic)	£5706	JA30Z	[10]

Cardiac catheterisation	£1142	EY43F	[10]
Walk in centre visit	£72.07	(T02A, T02NA, T03A, T03NA, T04A & T04NA)*	[10]
See & treat (no convey)	£209	ASS01	[10]
*Weighted average of codes			

Table 2 Unit costs relating to the most commonly reported HRGs at baseline and at 24-month time horizon

**Admitted patient care**							
**HRG code**	**Description**			**Elective**	**NEL**	**NES**	**Day case**
AA26G	Muscular, Balance, Cranial or Peripheral Nerve Disorders, Epilepsy or Head Injury, with CC Score 3-5			£3051	£1924	£416	£549
AA26H	Muscular, Balance, Cranial or Peripheral Nerve Disorders, Epilepsy or Head Injury, with CC Score 0-2			£2358	£1713	£357	£595
AA33C	Conventional EEG, EMG or Nerve Conduction Studies, 19 years and over			£1952	£2993	£827	£807
AA80Z	Complex Long-Term EEG Monitoring			£2126	£2960	£1182	£901
PR02B	Paediatric Epilepsy Syndrome with CC Score 1-5			£2835	£3242	£602	£998
PR02C	Paediatric Epilepsy Syndrome with CC Score 0			£1800	£2741	£564	£742
SB97Z	Same Day Chemotherapy Admission or Attendance			£308	£3014	£382	£110
SC97Z	Same Day Radiotherapy Admission or Attendance (excluding Brachytherapy)			£972	-	£287	£1389
WH04E	Poisoning Diagnosis without Interventions, with CC Score 0-1			£1176	£1347	£383	£362
WH50B	Procedure Not Carried Out, for Other or Unspecified Reasons			£578	£1995	£477	£330
**Outpatients**							
**Service**		**Currency**		**Consultation**		**Procedure**	
110	Trauma & Orthopaedics	WF01A	Non-Admitted Face-to-Face Attendance, Follow-up	£120		£245	
110	Trauma & Orthopaedics	N/A	N/A	£120		N/A	
223	Paediatric epilepsy	N/A	N/A	£203		N/A	
320	Cardiology	WF01A	Non-Admitted Face-to-Face Attendance, Follow-up	£139		£193	
400	Neurology	WF01A	Non-Admitted Face-to-Face Attendance, Follow-up	£177		£697	
400	Neurology	WF01B	Non-Admitted Face-to-Face Attendance, First	£177		£410	
400	Neurology	N/A	N/A	£177		N/A	
420	Paediatrics	WF01A	Non-Admitted Face-to-Face Attendance, Follow-up	£217		£889	
421	Paediatric neurology	WF01A	Non-Admitted Face-to-Face Attendance, Follow-up	£339		£1099	
650	Physiotherapy	WF01A	Non-Admitted Face-to-Face Attendance, Follow-up	£58		£80	
**Accident & emergency**							
**Service**		**Currency**					

N/A	N/A	ASS02	See and treat and convey	£257
T01A	Type 01 admitted	VB04Z	Emergency Medicine, Category 2 Investigation with Category 4 Treatment	£318
T01A	Type 01 admitted	VB08Z	Emergency Medicine, Category 2 Investigation with Category 1 Treatment	£220
T01A	Type 01 admitted	VB09Z	Emergency Medicine, Category 1 Investigation with Category 1-2 Treatment	£159
T01NA	Type 01 non admitted	VB07Z	Emergency Medicine, Category 2 Investigation with Category 2 Treatment	£200
T01NA	Type 01 non admitted	VB08Z	Emergency Medicine, Category 2 Investigation with Category 1 Treatment	£179
T01NA	Type 01 non admitted	VB09Z	Emergency Medicine, Category 1 Investigation with Category 1-2 Treatment	£133
T01NA	Type 01 non admitted	VB11Z	Emergency Medicine, No Investigation with No Significant Treatment	£114
T03NA	Type 03 non admitted	VB09Z	Emergency Medicine, Category 1 Investigation with Category 1-2 Treatment	£68
T04NA	Type 04 non admitted	VB09Z	Emergency Medicine, Category 1 Investigation with Category 1-2 Treatment	£53

NEL: Non-elective long-stay; NES: Non-elective short-stay

Table 3: Unit costs relating to trial anti-seizure medicines

ASD	Formulation	Strength	N / vol	Unit cost (£)
Lamotrigine				
	Dispersible tablet	2mg	30	18.81
	Dispersible tablet	5mg	28	7.67
	Dispersible tablet	25mg	56	4.70
	Dispersible tablet	100mg	56	6.29
	Tablet	25mg	56	1.89
	Tablet	50mg	56	2.46
	Tablet	100mg	56	3.48
	Tablet	200mg	56	4.37
Levetiracetam				
	Tablet	250mg	60	5.72
	Tablet	500mg	60	9.97
	Tablet	750mg	60	8.96
	Tablet	1g	60	14.97
	Oral solution, sugar free	100mg/ml	300	7.71
Zonisamide				
	Capsule	25mg	14	7.55
	Capsule	50mg	56	40.01
	Capsule	100mg	56	5.27

Medication costs were taken from the British National Formulary (BNF) using drug tariff prices where available,[12] else the NHS indicative price, and the Prescription Costs Analysis (PCA) for England.[13] Unit costs for trial anti-seizure medications are presented in Table 3. Unless otherwise specified in the data, children aged 9 and over were assumed to be prescribed tablets or capsules, whilst children aged 8 and under were assumed to be prescribed an alternative form (e.g. solution, dispersible) where available.

The cost of each medicine was calculated by calculating the price per dose and multiplying by the quantity prescribed (e.g. number of tablets, capsules, inhalers or prefilled syringes), and the number of days of treatment.

All costs are at 2019/2020 prices and were discounted in the base-case analysis at the NICE recommended rate of 3.5% per annum.[6]

Health Utilities

The primary health outcome measure for the economic analysis was the quality-adjusted life year (QALY), generated from utility data measured using the EuroQol 5-dimension 3-level (EQ-5D-3L) questionnaire.[22] Secondary economic outcome measures were the EQ-VAS, and an epilepsy-specific utility measure, the NEWQOL-6D.[23]

The EQ-5D descriptive system includes five dimensions, relating to mobility, self-care, usual activities, pain and discomfort, and anxiety. For the EQ-5D-3L and EQ-5D-3L-Y, each dimension is measured against 3 statements (no problems, some problems and extreme problems), scored 1, 2

and 3, respectively. The NEWQOL-6D is an epilepsy-specific measure that includes domains of worry, depression, memory, concentration, control and stigma. Responses are measured according to 4 categories. Utility scores are obtained from the EQ-5D-3L-Y, EQ-5D-3L, EQ-5D-3L proxy and NEWQOL-6D using UK tariff values.[23,24]

For participants aged 8 to 15, self-reported responses to the EQ-5D-3L-Y were used, or if not available, proxy questionnaire responses (EQ-5D-3L and NEWQOL-6D), completed by a parent or carer. For participants aged 5-7 years, only proxy questionnaires were administered. All participants aged 8 years or over were administered the EQ-VAS.

All economic outcome measures were completed during the baseline visit, and annually thereafter (up to 60 months), and from Protocol version 7 onwards, were also provided during outpatient visits to aid completeness. Utility scores at 365 days (12 months) and at 730 days (24 months) were interpolated, based on recorded utility scores and actual dates of questionnaire completion. QALY profiles were derived from these utilities, estimated based on the area under the curve (AUC) assuming the trapezoidal rule using all available data. QALYs derived from the secondary health economic outcomes (EQ-VAS and NEWQOL-6D) were estimated in the same way, based on AUC.

All QALYs were discounted at the NICE recommended rate of 3.5% per annum.[6]

Data analysis

Analysis consisted of all randomised participants, which is consistent with the intention to treat approach. All statistical tests were two-sided, with confidence intervals (CIs) and central ranges (CRs) reported at 97.5%.

Costs relating to secondary care were primarily sourced from HES data, but where these data were not available, costs were supplemented with resource-use recorded in the self-report questionnaires. Primary and community care costs and concomitant medication costs were also taken from the resource-use questionnaires. Where resource-use questionnaires were returned, but no response was provided for a given resource, then use of that resource was assumed to be zero. Where participants indicated that they had used a resource but had not given a number for how many times the resource was used, then the number was assumed to be one. Data relating to anti-seizure medications were taken from the baseline and follow-up CRFs. Missing dose data were assigned according to previous or subsequent prescriptions, based on questions relating to dose changes, and where these were unavailable, from the BNF recommended doses.

Data were examined for missingness, and appropriate methods were applied dependent on the level of missingness and likely mechanism of missingness.[25] Missing cost and QALY data were imputed using multiple imputation with chained equations.[26] When the mechanism of data missingness is not missing completely at random, complete case analysis can lead to serious bias which can reverse decisions of cost-effectiveness.[25] Multiple imputation is a flexible approach which provides unbiased results when data are missing at random.[25,26]

In order to maximise data use, data were imputed at the level of utility scores (EQ-5D, EQ-VAS) at baseline, 12 months and 24 months; primary care, community care and concomitant medications costs at 3 months, 6 months, 12 months and 24 months; admitted patient care, outpatients, accident and emergency and anti-seizure medication costs) at 12 months and 24 months. Baseline costs (relating to admitted patient care, outpatients, accident and emergency) were also imputed for those participants where HES data were not available. Imputation models were generated using

predictive mean matching, and data were imputed by randomised treatment group. Variables pertaining to epilepsy classification, seizure type, age, gender, primary outcome and treatment failure were included within the imputation models. Imputation models for baseline measures omitted post-baseline outcomes in order to preserve randomisation. The number of imputations required was based on the level of missingness, according to the fraction of missing information (FMI).[27]

Based on the imputed data, total costs and QALYs during the course of the trial were calculated, with summary statistics generated by randomised treatment group. Differences between treatment groups were compared with reference to bootstrapped central ranges, based on 10,000 replications.

Total costs and QALYs (at 24 months) were adjusted for any imbalances in baseline costs and utilities respectively, and clinical or demographic variables (age, sex, epilepsy classification, with centre as random effects), using ordinary least squares (OLS) regressions.[28,29] OLS was considered to be appropriate given the large sample size.[29]

Incremental analysis

Interventions were ranked according to their effectiveness (from highest to lowest QALYs), and dominance and extended dominance were determined. The incremental cost effectiveness ratio (ICER) was calculated for non-dominated interventions, as:

ICER = (Difference in costs) / (difference in QALY)

Net health benefits (NHB), and incremental net health benefits (INHB) were also calculated at the £20,000 per QALY and £30,000 per QALY thresholds, according to the following formulae:

NHB = (QALYs) – λ . (Costs)

INHB = (Difference in QALYs) – λ . (Difference in costs)

Where λ is the cost-effectiveness threshold.[30]

The base-case was defined as being from the perspective of the NHS and PSS, adopting a 2-year time horizon, and based on the imputed data set of the intention to treat population, with adjusted costs and QALYs.

Sensitivity analysis

Several sensitivity analyses were conducted to assess the robustness of the base-case results to key assumptions. These were:

1) using discount rates of 0% and 6% per annum for costs and QALYs;
2) an unadjusted analysis (i.e. based on mean costs and QALYs, with no regression);
3) using results for complete case cost and QALY data (i.e. those without missing data) to identify the impact of missing data and imputation;
4) based on the population as the per protocol cohort; and
5) using QALYs derived from the NEWQOL-6D and EQ-VAS
6) treating blank values in resource use questionnaires as missing, rather than zero.

A bootstrap analysis was conducted to consider the joint uncertainty in incremental costs and QALYs. This was represented as a cost-effectiveness plane, and as a cost-effectiveness acceptability curve (CEAC) illustrating the probability of each treatment being cost effective for a given cost-effectiveness threshold.[31]

Subgroup analysis

Subgroup analyses were conducted to investigate how cost-effectiveness varied by age, according to whether participants were adults (aged 16 and over) or children (aged under 16).

Results:

HES data were available for a total of 772 participants, relating to 266 participants randomised to lamotrigine, 261 participants in the levetiracetam treatment group and 245 participants randomised to zonisamide. A breakdown of missing data by treatment group and outcome is provided in Table 4.

Seven-hundred and eighty-nine participants completed at least one self-report questionnaire (completing either resource use, EQ-5D, or both sections); 621 completed two questionnaires or more. In total, questionnaires were available for 3039 participant-time points (once child and proxy questionnaires had been resolved).

Questionnaires returned after the change in protocol were assigned to their nearest time-point for presentation purposes. Self-report resource use data were available for 550 participants at 3 months, 527 at 6 months, 465 at 12 months and 398 at 24 months. Resource use data were also available from 496 questionnaires returned at the later time points (36 months, 48 months, 60 months).

Utility data (EQ-5D) were available for 616 participants at baseline, data were interpolated to 12 months for 422 participants and for 319 participants at 24 months. These are lower than the figures reported in Table 4 due to 12- and 24-month questionnaires being dated less than 365 and 730 days post randomisation, respectively. For the NEWQOL-6D, less utility data were available due to a high level of partially completed questionnaires.

A total of 50 data sets were imputed, based on the largest FMI (0.7) and accepting <1% reduction in power compared with 100 imputations. For the bootstrapped results, this was reduced to 10 for efficiency purposes, accepting a higher reduction in power in order to achieve an acceptable computation time.[27] Due to the level of missingness, models containing the NEWQOL-6D were non-convergent, hence only complete case results are presented for the NEWQOL-6D.

Table 4: Summary of data completeness by outcome, time point and treatment group

		Lamotrigine			Levetiracetam			Zonisamide		
Variable		Complete	Incomplete	Total	Complete	Incomplete	Total	Complete	Incomplete	Total
Costs	Time point	Participants (n)								
Admitted patient care	Baseline	266	64	330	261	71	332	245	83	328
Outpatients	Baseline	266	64	330	261	71	332	245	83	328
Accident & emergency	Baseline	266	64	330	261	71	332	245	83	328
Primary care	3 months	182	148	330	186	146	332	182	146	328
Community care	3 months	182	148	330	186	146	332	182	146	328
Concomitant medication	3 months	182	148	330	186	146	332	182	146	328
Primary care	6 months	177	153	330	176	156	332	174	154	328
Community care	6 months	177	153	330	176	156	332	174	154	328
Concomitant medication	6 months	177	153	330	176	156	332	174	154	328
Primary care	12 months	156	174	330	154	178	332	155	173	328
Community care	12 months	156	174	330	154	178	332	155	173	328
Admitted patient care	12 months	298	34	330	286	47	332	272	56	328
Outpatients	12 months	298	34	330	286	47	332	272	56	328
Accident & emergency	12 months	298	34	330	286	47	332	272	56	328
Anti-seizure medication	12 months	291	39	330	293	39	332	280	48	328
Concomitant medication	12 months	156	174	330	154	178	332	155	173	328
Primary care	24 months	135	195	330	133	199	332	130	198	328
Community care	24 months	135	195	330	133	199	332	130	198	328
Admitted patient care	24 months	299	32	330	291	43	332	280	48	328
Outpatients	24 months	299	32	330	291	43	332	280	48	328
Accident & emergency	24 months	299	32	330	291	43	332	280	48	328
Anti-seizure medication	24 months	257	73	330	260	72	332	239	89	328
Concomitant medication	24 months	135	195	330	133	199	332	130	198	328
Primary care	36 months	93	175	268	92	174	266	84	183	267
Community care	36 months	93	175	268	92	174	266	84	183	267
Admitted patient care	36 months	236	32	268	225	41	266	217	50	267
Outpatients	36 months	236	32	268	225	41	266	217	50	267
Accident & emergency	36 months	236	32	268	225	41	266	217	50	267

Anti-seizure medication	36 months	125	143	268	134	132	266	118	149	267
Concomitant medication	36 months	93	175	268	92	174	266	84	183	267
Primary care	48 months	46	125	171	58	117	175	44	130	174
Community care	48 months	46	125	171	58	117	175	44	130	174
Admitted patient care	48 months	150	21	171	151	24	175	141	33	174
Outpatients	48 months	150	21	171	151	24	175	141	33	174
Accident & emergency	48 months	150	21	171	151	24	175	141	33	174
Anti-seizure medication	48 months	62	109	171	66	109	175	52	122	174
Concomitant medication	48 months	46	125	171	58	117	175	44	130	174
Primary care	60 months	26	54	80	29	50	79	24	53	77
Community care	60 months	26	54	80	29	50	79	24	53	77
Admitted patient care	60 months	74	6	80	69	10	79	59	18	77
Outpatients	60 months	74	6	80	69	10	79	59	18	77
Accident & emergency	60 months	74	6	80	69	10	79	59	18	77
Anti-seizure medication	60 months	19	61	80	22	57	80	16	61	80
Concomitant medication	60 months	26	54	80	29	50	79	24	53	77
Utilities										
EQ-5D	Baseline	209	121	330	202	130	332	205	123	328
NEWQOL-6D	Baseline	201	129	330	190	142	332	186	142	328
EQ-VAS	Baseline	188	142	330	187	145	332	190	138	328
EQ-5D	12 months	148	182	330	148	184	332	147	181	328
NEWQOL-6D	12 months	107	223	330	100	232	332	104	224	328
EQ-VAS	12 months	135	194	330	126	206	332	136	192	328
EQ-5D	24 months	121	209	330	124	208	332	122	206	328
NEWQOL-6D	24 months	87	243	330	88	244	332	80	248	328
EQ-VAS	24 months	116	214	330	111	221	332	114	214	328
EQ-5D	36 months	94	174	268	93	173	266	83	184	267
NEWQOL-6D	36 months	69	199	268	58	208	266	61	206	267
EQ-VAS	36 months	93	175	268	89	177	266	78	189	267
EQ-5D	48 months	50	121	171	58	117	175	46	128	174
NEWQOL-6D	48 months	37	134	171	41	134	175	33	141	174
EQ-VAS	48 months	48	123	171	55	120	175	43	131	174
EQ-5D	60 months	31	49	80	31	48	79	26	51	77

NEWQOL-6D	60 months	25	55	80	16	63	79	17	60	77
EQ-VAS	60 months	31	49	80	30	49	79	25	52	77

Resource use and costs

Table 5 presents observed mean disaggregated resource-use based on the self-report questionnaires. Table 6 presents the most common admitted patient care episodes, outpatient and accident and emergency related HRGs and costs observed during the trial period. During the 24-month follow-up period, 339 unique HRGs were recorded in admitted patient care, 262 in outpatients, and 35 in accident & emergency.

Based on the imputed data, the majority of costs related to secondary care, in particular admitted patient care and outpatient clinic attendance (Table 7). Comparing across treatment groups, participants randomised to zonisamide had higher secondary care costs compared with lamotrigine and levetiracetam. Total (unadjusted) costs for participants randomised to zonisamide were £5409 (97.5% CR £4584, £6658), compared with levetiracetam £5074 (97.5% CR £4433, £6049), and lamotrigine £4063 (97.5% CR £3617, £4842). The differences between zonisamide and levetiracetam £336 (97.5% CR -£926, £1634), and between levetiracetam and lamotrigine £1011 (97.5% CR -£36, £2066), were not statistically significant. However, the incremental cost of zonisamide versus lamotrigine of £1347 (97.5% CR £266, £2550) was significant.

Based on imputed data, baseline costs were £1,215 (97.5% CR £1061, £1375) for zonisamide, £1,191 (97.5% CR £1035, £1398) for levetiracetam, and £1,239 (97.5% CR £1036, £1464) for lamotrigine. The base-case analysis which adjusted for baseline costs, age, gender and epilepsy type with centre as random-effects yielded a 2-year total cost of £5400 (97.5% CR £4659, £6770) for zonisamide, compared with £5104 (97.5% CR £4450, £6141) for levetiracetam, and £4042 (97.5% CR £3626, £4983) for lamotrigine. The differences between zonisamide and levetiracetam £297 (97.5% CR -£388, £1550), was not statistically significant. There were significant differences between levetiracetam and lamotrigine £1,062 (97.5% CR £1174, £2133), and between zonisamide and lamotrigine £1,358 (97.5% CR £376, £2563).

Table 5: Observed resource-use based on self-report questionnaire (24-month time horizon)

		Mean [range] (n participants)											
Time point		3 months			6 months			12 months			24 months		
Questionnaires returned (n)		179	183	182	172	170	173	150	147	151	126	124	122
Resource	Treatment group	LTG	LEV	ZON	LTG	LEV	ZON	LTG	LEV	ZON	LTG	LEV	ZON
Primary care													
	GP consultation at GP surgery	1.02 [0-8] (90)	1.13 [0-13] (88)	0.98 [0-10] (92)	0.67 [0-5] (63)	0.87 [0-10] (72)	0.89 [0-12] (71)	0.76 [0-14] (65)	1.01 [0-12] (67)	1.10 [0-8] (76)	0.83 [0-9] (52)	1.09 [0-10] (56)	1.01 [0-20] (52)
	Nurse consultation at GP surgery	0.58 [0-11] (46)	0.50 [0-10] (42)	0.46 [0-10] (47)	0.42 [0-12] (45)	0.38 [0-6] (35)	0.56 [0-24] (42)	0.63 [0-12] (48)	0.71 [0-10] (51)	0.73 [0-8] (52)	0.83 [0-12] (51)	0.85 [0-8] (47)	0.74 [0-16] (41)
	GP home visit	0.01 [0-1] (1)	0.04 [0-6] (3)	0.05 [0-5] (5)	0.02 [0-2] (2)	0.04 [0-2] (5)	0.02 [0-2] (3)	0	0.02 [0-2] (2)	0.01 [0-1] (1)	0.02 [0-2] (1)	0.08 [0-6] (3)	0.02 [0-1] (3)
	Nurse home visit	0.10 [0-2] (14)	0.13 [0-6] (10)	0.05 [0-6] (4)	0.03 [0-1] (5)	0.37 [0-24] (11)	0.05 [0-12] (9)	0.01 [0-1] (1)	0.68 [0-95] (5)	0.01 [0-1] (1)	0.01 [0-1] (1)	0.19 [0-12] (10)	0.05 [0-2] (4)
Community care													
	Health visitor	0.01 [0-1] (2)	0.06 [0-6] (4)	0.04 [0-3] (4)	0.01 [0-1] (1)	0.06 [0-5] (3)	0.02 [0-3] (1)	0.01 [0-1] (1)	0	0.01 [0-2] (1)	0.03 [0-4] (1)	0.04 [0-3] (3)	0.02 [0-2] (1)
	Social worker	0.08 [0-7] (4)	0.04 [0-6] (3)	0.02 [0-2] (2)	0.06 [0-4] (3)	0.06 [0-6] (4)	0.03 [0-3] (3)	0.14 [0-20] (2)	0.07 [0-5] (4)	0.05 [0-4] (4)	0.02 [0-2] (2)	0.06 [0-4] (3)	0.06 [0-3] (4)
	Occupational therapist	0.09 [0-4] (9)	0.15 [0-6] (14)	0.09 [0-4] (9)	0.05 [0-3] (5)	0.10 [0-6] (7)	0.03 [0-2] (5)	0.17 [0-20] (5)	0.07 [0-3] (7)	0.03 [0-2] (3)	0.02 [0-2] (1)	0.29 [0-27] (5)	0.05 [0-5] (2)
	Psychologist	0.07 [0-4] (8)	0.16 [0-8] (10)	0.09 [0-5] (7)	0.06 [0-3] (7)	0.20 [0-18] (10)	0.06 [0-2] (8)	0.03 [0-2] (4)	0.14 [0-11] (5)	0.07 [0-2] (7)	0.07 [0-3] (5)	0.21 [0-6] (8)	0.25 [0-7] (9)
	Counsellor	0.02 [0-2] (2)	0.10 [0-6] (4)	0.18 [0-13] (6)	0.07 [0-6] (3)	0.20 [0-8] (7)	0.29 [0-12] (11)	0.09 [0-9] (4)	0.22 [0-12] (7)	0.15 [0-12] (5)	0.06 [0-6] (3)	0.21 [0-16] (8)	0.22 [0-12] (5)
	Physiotherapist	0.13 [0-6] (7)	0.16 [0-6] (10)	0.14 [0-6] (9)	0.09 [0-12] (4)	0.09 [0-4] (7)	0.13 [0-10] (7)	0.09 [0-7] (5)	0.32 [0-10] (11)	0.16 [0-12] (6)	0.13 [0-6] (6)	0.41 [0-27] (9)	22 [0-10] (7)
Secondary care													
	Doctor at hospital	0.55 [0-3] (74)	0.79 [0-6] (86)	0.70 [0-6] (83)	0.68 [0-3] (86)	1.05 [0-61] (85)	0.79 [0-6] (92)	0.61 [0-4] (64)	0.63 [0-8] (56)	0.64 [0-5] (72)	0.53 [0-6] (49)	0.60 [0-7] (51)	0.61 [0-8] (44)

	Nurse at hospital	0.47 [0-4] (66)	0.59 [0-6] (79)	0.59 [0-6] (77)	0.53 [0-16] (60)	0.46 [0-4] (62)	0.57 [0-6] (72)	0.47 [0-5] (53)	0.68 [0-13] (55)	0.53 [0-20] (45)	0.31 [0-5] (31)	0.41 [0-6] (38)	0.56 [0-10] (42)
	Hospital overnight	0.28 [0-18] (12)	0.16 [0-6] (13)	0.15 [0-7] (15)	0.09 [0-7] (8)	0.09 [0-5] (6)	0.12 [0-6] (10)	0.24 [0-16] (6)	0.52 [0-46] (7)	0.24 [0-10] (10)	0.09 [0-4] (6)	0.84 [0-77] (9)	0.39 [0-28] (9)
	Ambulance	0.18 [0-7] (21)	0.25 [0-7] (22)	0.17 [0-4] (19)	0.07 [0-2] (11)	0.14 [0-6] (13)	0.11 [0-3] (17)	0.08 [0-3] (9)	0.08 [0-2] (8)	0.15 [0-5] (14)	0.13 [0-2] (13)	0.10 [0-3] (9)	0.18 [0-5] (10)
	A&E visit	0.27 [0-7] (28)	0.30 [0-5] (31)	0.23 [0-4] (24)	0.15 [0-2] (22)	0.21 [0-4] (21)	0.21 [0-9] (25)	0.27 [0-8] (24)	0.30 [0-15] (18)	0.23 [0-6] (23)	0.20 [0-3] (19)	0.29 [0-4] (21)	0.24 [0-5] (20)
	Blood test	0.58 [0-11] (58)	0.36 [0-4] (51)	0.46 [0-24] (44)	0.34 [0-12] (42)	0.70 [0-59] (43)	0.46 [0-10] (44)	0.60 [0-16] (45)	0.48 [0-10] (41)	0.50 [0-7] (47)	0.73 [0-12] (47)	0.63 [0-7] (42)	0.52 [0-5] (40)
	Urine test	0.14 [0-4] (20)	0.13 [0-3] (20)	0.22 [0-14] (23)	0.12 [0-2] (18)	0.29 [0-28] (18)	0.18 [0-3] (24)	0.16 [0-3] (18)	0.13 [0-2] (14)	0.07 [0-2] (9)	0.15 [0-3] (16)	0.15 [0-2] (14)	0.28 [0-9] (17)
	Ultrasound	0.09 [0-2] (16)	0.09 [0-3] (13)	0.09 [0-3] (13)	0.06 [0-2] (9)	0.05 [0-3] (7)	0.13 [0-2] (18)	0.07 [0-1] (9)	0.05 [0-4] (5)	0.08 [0-2] (10)	0.04 [0-2] (4)	0.07 [0-2] (8)	0.14 [0-4] (12)
	X-Ray	0.13 [0-6] (10)	0.10 [0-3] (13)	0.15 [0-8] (16)	0.08 [0-3] (10)	0.11 [0-2] (15)	0.16 [0-4] (20)	0.21 [0-3] (25)	0.08 [0-3] (8)	0.09 [0-2] 10	0.19 [0-6] (16)	0.16 [0-5] (14)	0.16 [0-3] (15)
	CT scan	0.07 [0-2] (11)	0.08 [0-2] (14)	0.08 [0-2] (14)	0.03 [0-1] (6)	0.04 [0-1] (7)	0.04 [0-1] (7)	0.05 [0-2] (7)	0.03 [0-2] (3)	0.01 [0-1] (2)	0.02 [0-1] (2)	0.02 [0-1] (3)	0.01 [0-1] (1)
	MRI scan	0.21 [0-2] (36)	0.21 [0-2] (37)	0.24 [0-2] (41)	0.06 [0-2] (10)	0.06 [0-1] (11)	0.09 [0-2] (15)	0.07 [0-2] (9)	0.01 [0-1] (1)	0.05 [0-1] (7)	0.02 [0-1] (2)	0.02 [0-1] (2)	0.02 [0-1] (3)
	EEG	0.21 [0-4] (33)	0.15 [0-2] (26)	0.18 [0-2] (32)	0.04 [0-1] (7)	0.05 [0-1] (8)	0.03 [0-1] (6)	0.03 [0-1] (4)	0.01 [0-1] (2)	0.04 [0-2] (5)	0.01 [0-1] (1)	0.01 [0-1] (1)	0.01 [0-1] (1)
	Other*	0.11 [0-2] (18)	0.12 [0-3] (19)	0.16 [0-7] (19)	0.09 [0-2] (12)	0.12 [0-2] (19)	0.35 [0-18] (18)	0.09 [0-2] (11)	0.07 [0-1] (10)	0.10 [0-2] (14)	0.42 [0-28] (14)	0.17 [0-10] (10)	0.20 [0-3] (18)

**Primary care*: GP out of hours, telephone consultation (GP), MMR, repeat prescription, saliva test
Community care: Dentist, orthodontist, school nurse, SENCO, speech therapist, support worker, psychiatrist, Midwife, CAHMS, optician, NHS glasses, cervical smear, podiatrist, podiatrist minor surgery, dietician, NHS direct, hearing test, mammogram
Outpatients: Anticoagulant service, long term EEG monitoring, ECG, sleep apnoea test, endoscopy, cystoscopy, contrast fluoroscopy, grommets, tooth extraction, cerebral angiogram, audiologist, PET scan, nasal polypectomy, radio frequency treatment, colonoscopy, minor skin procedures, field exercise test, FESS operation, dexa scan, video telemetry, spinal fluid test, diabetic retinopathy screening, percutaneous biopsy, rib fracture, liver biopsy, radiotherapy, hand fracture, arm fracture, MRSA swabs, prostate biopsy, biopsy (nose, external), cardiac catheterisation, peak flow test, minor dental procedures

Admitted patient care: hernia operation, pelvis fracture, implantation of loop recorder, removal of loop recorder, Vaginal tape operation, overnight sleep study, triple heart bypass, foot operation, pacemaker fitted, cholecystectomy, bursa excision, hysterectomy, knee replacement, cyst removal
Accident & emergency: See & treat (no convey), Walk in centre

Table 6. Unit costs of admitted patient care, outpatient and accident & emergency hospital attendances for the most frequent HRG codes for the 24-month trial period. Rounded to nearest 5, * indicates < 10.

**HRG code**	**Description**			**Attendances**			
				**LTG**	**LEV**	**ZON**	**Total**
**Admitted patient care**							
AA26H	Muscular, Balance, Cranial or Peripheral Nerve Disorders, Epilepsy or Head Injury, with CC Score 0-2			15	20	20	60
SC97Z	Same Day Radiotherapy Admission or Attendance (excluding Brachytherapy)			20	0	20	40
AA26G	Muscular, Balance, Cranial or Peripheral Nerve Disorders, Epilepsy or Head Injury, with CC Score 3-5			*	*	*	25
SB97Z	Same Day Chemotherapy Admission or Attendance			25	0	0	25
AA33C	Conventional EEG, EMG or Nerve Conduction Studies, 19 years and over			*	*	*	20
PR02B	Paediatric Epilepsy Syndrome with CC Score 1-5			*	*	*	20
AA80Z	Complex Long-Term EEG Monitoring			*	*	*	15
PR02C	Paediatric Epilepsy Syndrome with CC Score 0			*	*	*	15
WH50B	Procedure Not Carried Out, for Other or Unspecified Reasons			*	*	*	10
WH04E	Poisoning Diagnosis without Interventions, with CC Score 0-1			*	*	*	10
**Outpatients**							
400	Neurology	WF01A	Non-Admitted Face-to-Face Attendance, Follow-up	800	840	825	2465
400	Neurology	WF01B	Non-Admitted Face-to-Face Attendance, First	195	185	160	540
420	Paediatrics	WF01A	Non-Admitted Face-to-Face Attendance, Follow-up	120	155	145	420
400	Neurology	N/A	N/A	65	80	80	220
110	Trauma & Orthopaedics	WF01A	Non-Admitted Face-to-Face Attendance, Follow-up	70	60	65	200
650	Physiotherapy	WF01A	Non-Admitted Face-to-Face Attendance, Follow-up	30	55	50	135
421	Paediatric neurology	WF01A	Non-Admitted Face-to-Face Attendance, Follow-up	50	45	22	120
223	Paediatric epilepsy	N/A	N/A	20	20	80	115
110	Trauma & Orthopaedics	N/A	N/A	40	45	30	115

320	Cardiology	WF01A	Non-Admitted Face-to-Face Attendance, Follow-up	30	40	35	105
**Accident & emergency**							
N/A	N/A	ASS02	See and treat and convey	140	170	185	490
T01NA	Type 01 non admitted	VB09Z	Emergency Medicine, Category 1 Investigation with Category 1-2 Treatment	105	100	90	295
T01NA	Type 01 non admitted	VB08Z	Emergency Medicine, Category 2 Investigation with Category 1 Treatment	50	55	70	180
T01NA	Type 01 non admitted	VB11Z	Emergency Medicine, No Investigation with No Significant Treatment	30	25	30	85
T01A	Type 01 admitted	VB09Z	Emergency Medicine, Category 1 Investigation with Category 1-2 Treatment	25	30	25	75
T01A	Type 01 admitted	VB08Z	Emergency Medicine, Category 2 Investigation with Category 1 Treatment	20	25	25	65
T01NA	Type 01 non admitted	VB07Z	Emergency Medicine, Category 2 Investigation with Category 2 Treatment	15	30	20	60
T04NA	Type 04 non admitted	VB09Z	Emergency Medicine, Category 1 Investigation with Category 1-2 Treatment	*	*	*	45
T01A	Type 01 admitted	VB04Z	Emergency Medicine, Category 2 Investigation with Category 4 Treatment	15	15	15	45
T03NA	Type 03 non admitted	VB09Z	Emergency Medicine, Category 1 Investigation with Category 1-2 Treatment	*	*	*	35

CC – complication or comorbidity

Table 7. Aggregated cost totals (imputed, discounted)

Time period	Totals (discounted) at 24 months			Difference		
Arm	LTG	LEV	ZON	LEV-LTG	ZON-LTG	ZON-LEV
**Primary and Community care**	682 (551, 1018)	1303 (981, 2009)	1013 (786, 1631)	622 (148, 1274)	331 (-31, 940)	-290 (-979, 398)
**Primary care** Mean [95% CR]	332 (284, 423)	532 (416, 724)	411 (347, 567)	200 (59, 391)	79 (-25, 236)	-121 (-306, 82)
**Community care** Mean [95% CR]	350 (228, 646)	771 (489, 1381)	602 (374, 1117)	422 (5, 1028)	253 (-95, 778)	-169 (-795, 409)
**Secondary care**	3025 (2606, 3628)	3263 (2853, 3723)	3882 (3140, 4670)	237 (-486, 847)	857 (-69, 1680)	619 (-215, 1509)
**Admitted patient care** Mean [95% CR]	1170 (855, 1631)	1156 (869, 1443)	1663 (1153, 2246)	-15 (-560, 400)	493 (-178, 1127)	507 (-75, 1207)
**Outpatient** Mean [95% CR]	1519 (1393, 1664)	1705 (1552, 1876)	1784 (1547, 2050)	186 (-26, 401)	266 (-17, 564)	80 (-202, 392)
**Accident & emergency** Mean [95% CR]	336 (269, 425)	402 (314, 528)	434 (316, 582)	66 (-64, 199)	98 (-55, 259)	32 (-153, 220)
**Medicines**	356 (294, 475)	508 (412, 665)	515 (423, 668)	151 (-10, 304)	158 (15, 316)	7 (-154, 193)
**Anti-seizure medication** Mean [95% CR]	125 (103, 158)	248 (213, 292)	269 (244, 298)	28 (75, 171)	14 (104, 184)	-14 (-24, 68)
**Concomitant medication** Mean [95% CR]	231 (175, 348)	260 (172, 403)	246 (161, 390)	123 (-122, 171)	144 (-126, 168)	21 (-165, 162)
**TOTAL**	4063 (3617, 4842)	5074 (4433, 6049)	5409 (4584, 6658)	1011 (-36, 2066)	1347 (266, 2550)	336 (-926, 1634)

Utilities and Quality adjusted life years

The distribution of participants' responses to the EQ-5D-3L-Y and the NEWQOL-6D questionnaires by randomised treatment group are presented in Figures 1 and 2. Based on imputed data, baseline utilities were 0.766 (97.5% CR 0.733, 0.804) for levetiracetam, 0.800 (97.5% CR 0.760, 0.830) for zonisamide and 0.779 (97.5% CR 0.751, 0.818) for lamotrigine. In the base-case, adjusted analysis, levetiracetam was associated with a QALY of 1.474 (97.5% CR 1.393, 1.523) over the 2-year time horizon, whilst zonisamide was associated with a QALY of 1.502 (97.5% CR 1.418, 1.566), compared with lamotrigine 1.605 (97.5% CR 1.547, 1.651). This corresponded to a negative incremental QALY of -0.025 (97.5% CR -0.058, 0.129) between levetiracetam and zonisamide. The incremental QALYs of -0.103 (97.5% CR -0.201, -0.015) between zonisamide and lamotrigine, and -0.128 (97.5% CR -0.219, -0.065) between levetiracetam and lamotrigine were significant.

QALYs based on the NEWQOL-6D were calculated for complete case data only, over the 2-year time horizon. Levetiracetam was associated with adjusted QALYs of 1.703 (97.5% CR 1.678, 1.727) compared with 1.712 (97.5% CR 1.690, 1.735) for zonisamide, and 1.710 (97.5% CR 1.687, 1.733) for lamotrigine. Levetiracetam was therefore associated with a negative incremental QALY of -0.009 (97.5% CR -0.033, 0.019) compared with zonisamide, and associated with a negative incremental QALY of -0.010 (97.5% CR -0.035, 0.019) compared with lamotrigine. The incremental QALY between zonisamide and lamotrigine was 0.002 (97.5% CR -0.021, 0.025).

The distribution of responses to the EQ-VAS is illustrated in Table 8. The adjusted analysis based on the EQ-VAS resulted in a QALY of 1.398 (97.5% CR 1.324, 1.479) for levetiracetam, 1.418 (97.5% CR 1.351, 1.456) for zonisamide, and 1.431 (97.5% CR 1.360, 1.476) for lamotrigine. The negative incremental QALYs of -0.020 (97.5% CR -0.094, 0.085) for levetiracetam versus zonisamide, -0.0.013 (97.5% CR -0.085, 0.060) for zonisamide versus lamotrigine, and -0.033 (97.5% CR -0.112, 0.075) for levetiracetam versus lamotrigine are consistent with the base-case EQ-5D.

Figure 1. Distribution of participants' responses to each EQ-5D attribute, by treatment allocated and time. Levels range from 1 to 3, with 3 representing the most severe problem. The percentage of completed responses (%). (a) Mobility; (b) self-care; (c) usual activities; (d) pain or discomfort; (e) anxiety or depression.

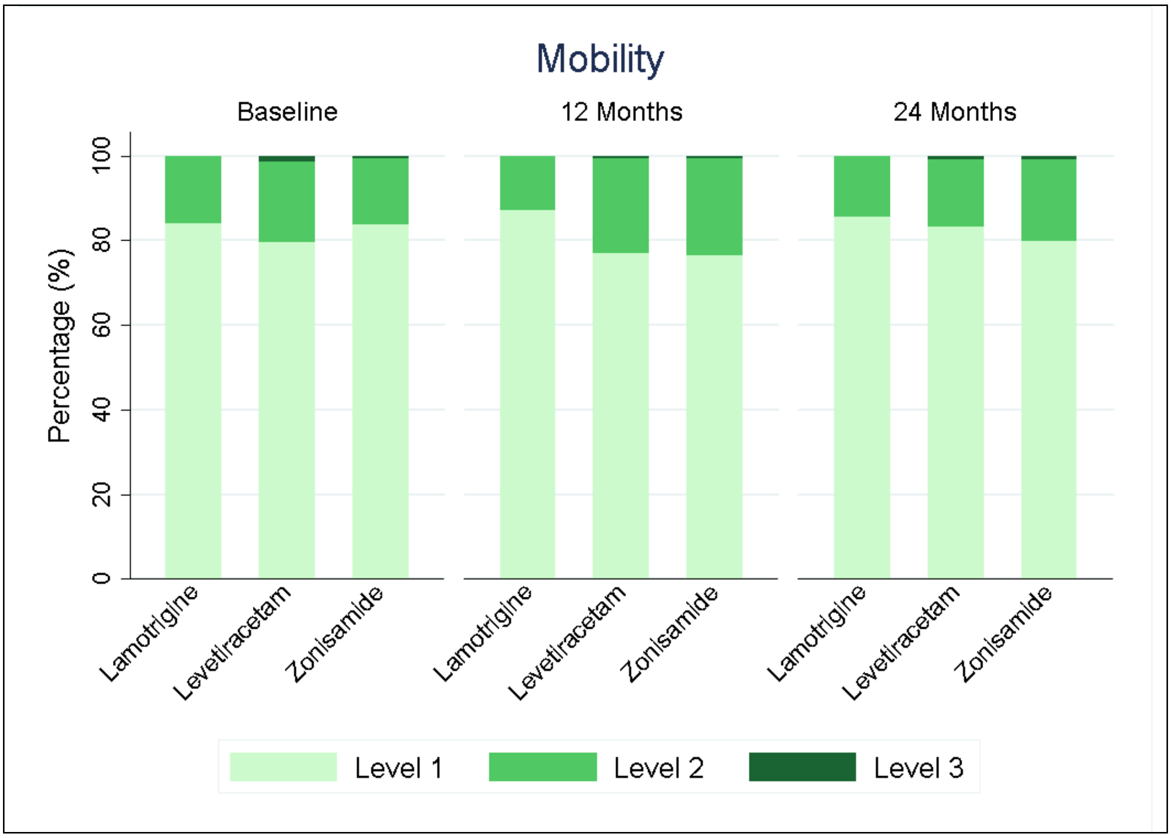

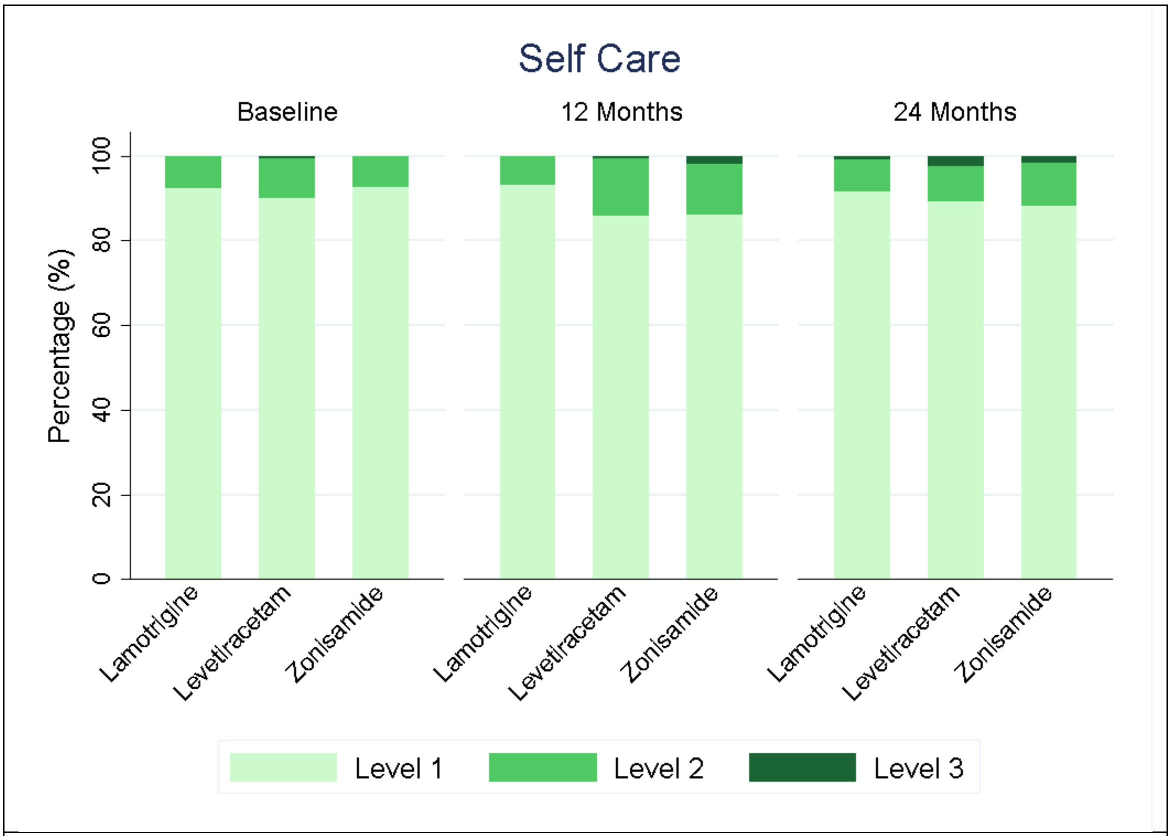
Self Care
Baseline
12 Months
24 Months
Percentage (%)
0
20
40
60
80
100
Lamotrigine
Levetiracetam
Zonisamide
Level 1
Level 2
Level 3

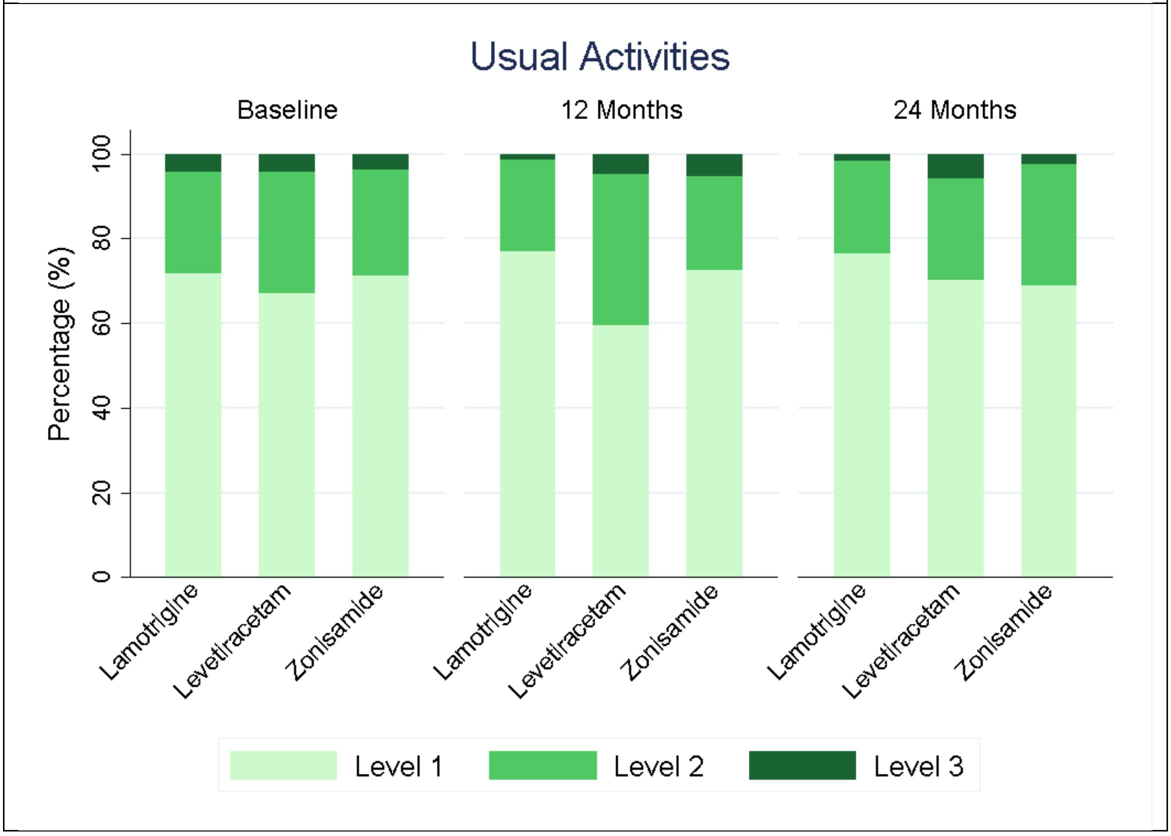
Usual Activities
Baseline
12 Months
24 Months
Percentage (%)
0
20
40
60
80
100
Lamotrigine
Levetiracetam
Zonisamide
Level 1
Level 2
Level 3

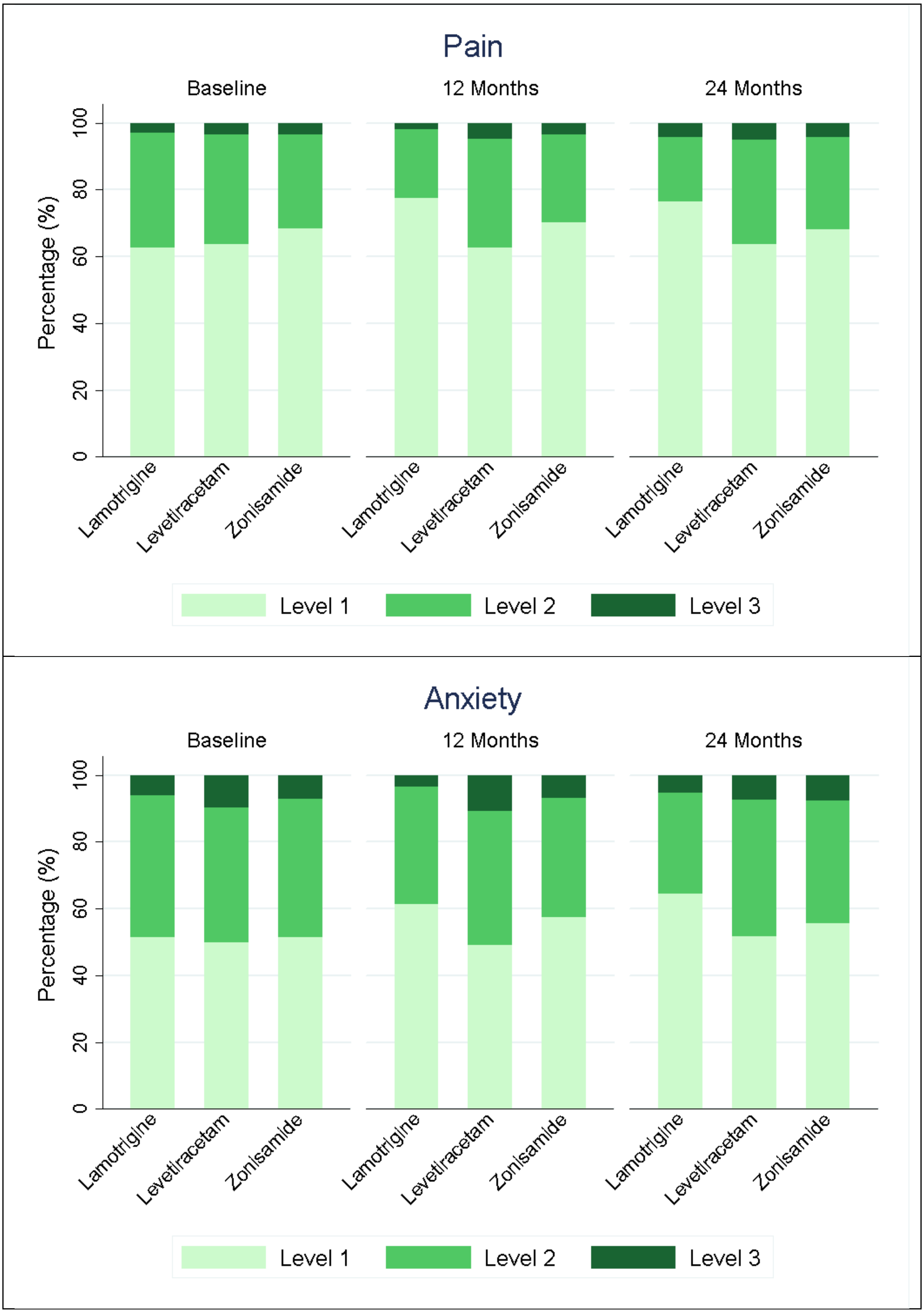
Pain
Baseline
12 Months
24 Months
Percentage (%)
0
20
40
60
80
100
Lamotrigine
Levetiracetam
Zonisamide
Level 1
Level 2
Level 3
Anxiety
Baseline
12 Months
24 Months
Percentage (%)
0
20
40
60
80
100
Lamotrigine
Levetiracetam
Zonisamide
Level 1
Level 2
Level 3

Figure 2. Distribution of participants' responses to each NEWQOL-6D attribute, by treatment allocated and time. Levels range from 1 to 4, with 4 representing the most severe problem. The percentage of completed responses (%). (a) Worry; (b) Depression; (c) Memory; (d) Concentration; (e) Control; (f) Stigma.

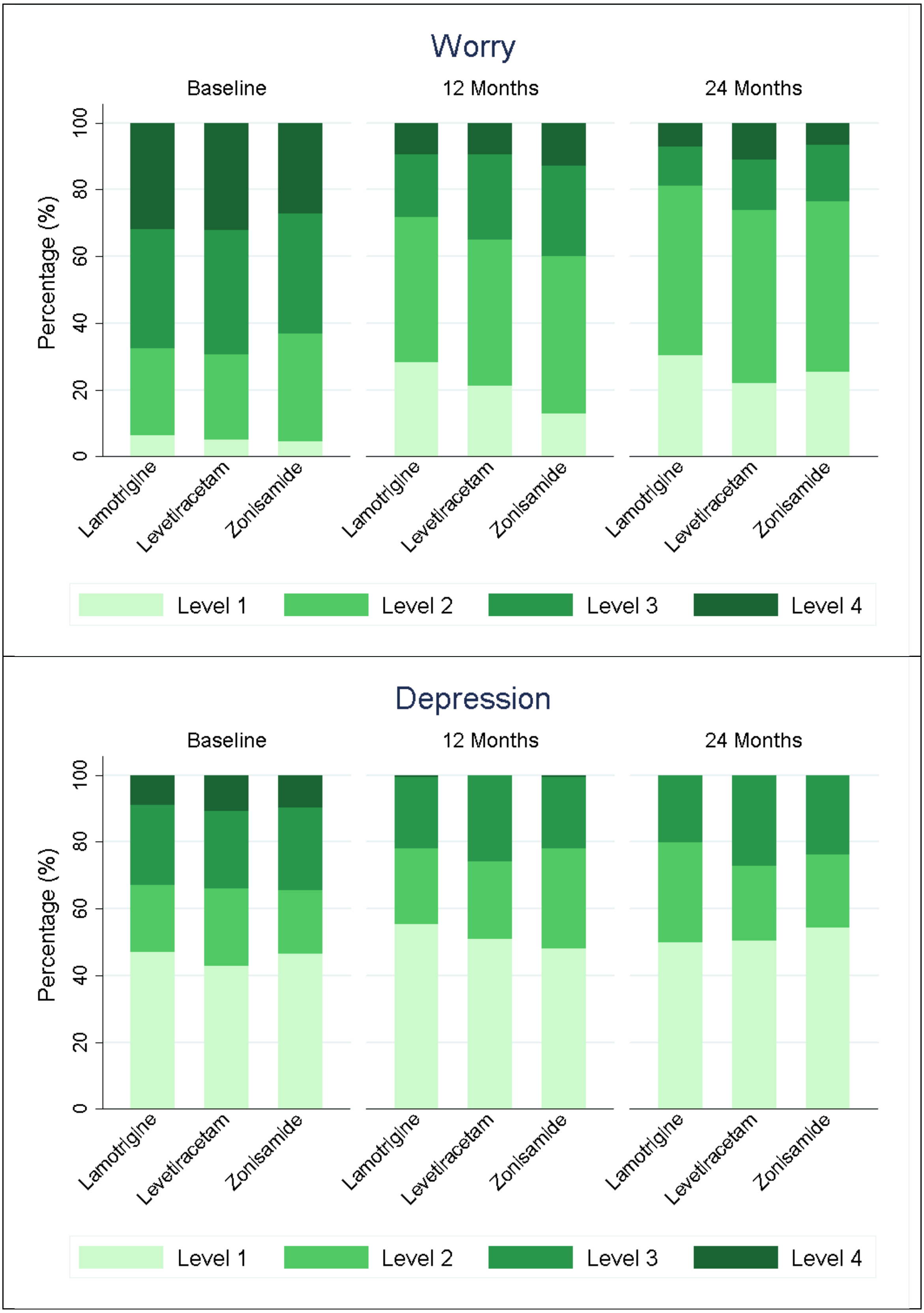

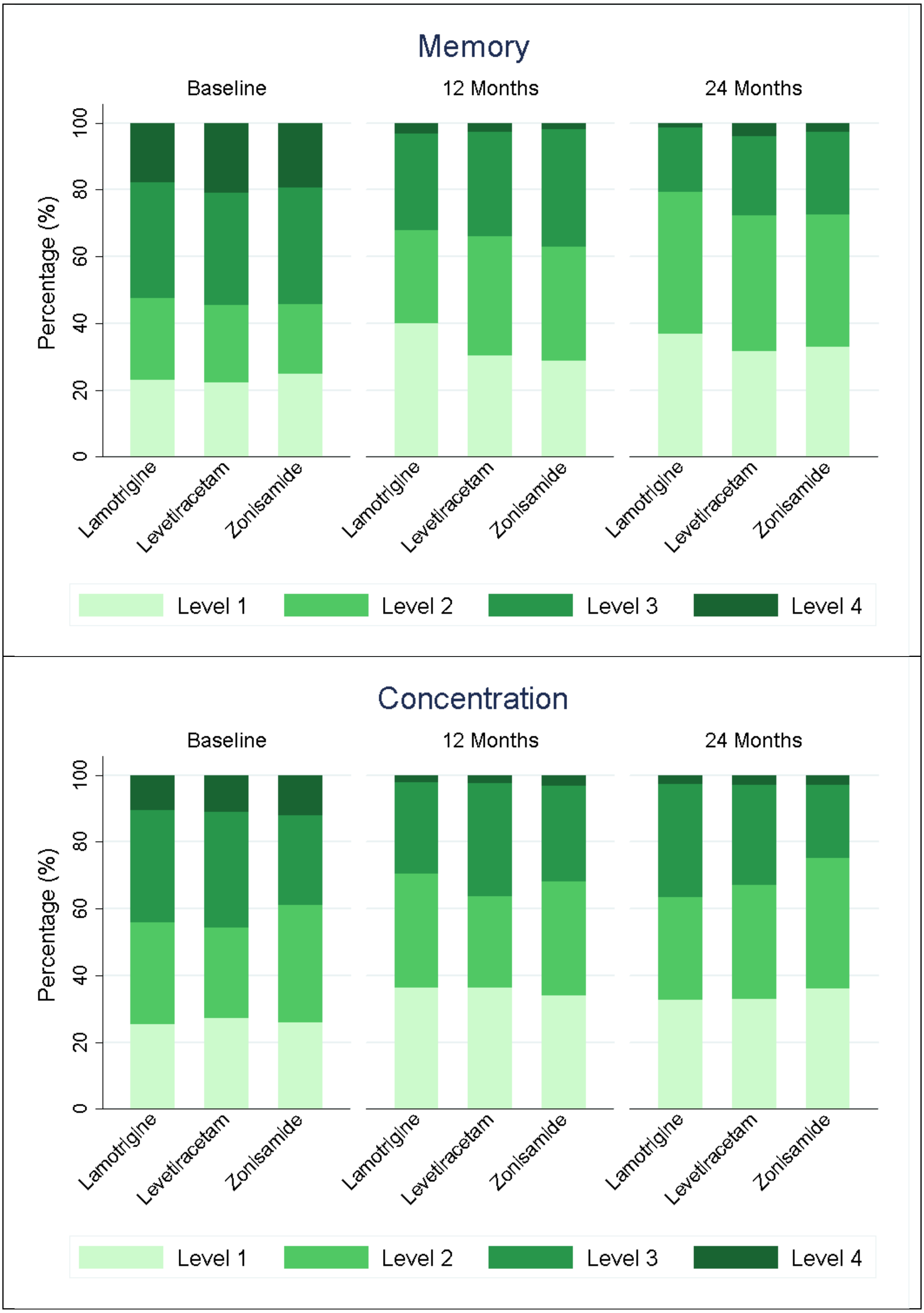
Memory
Baseline
12 Months
24 Months
Percentage (%)
0
20
40
60
80
100
Lamotrigine
Levetiracetam
Zonisamide
Level 1
Level 2
Level 3
Level 4
Concentration
Baseline
12 Months
24 Months
Percentage (%)
0
20
40
60
80
100
Lamotrigine
Levetiracetam
Zonisamide
Level 1
Level 2
Level 3
Level 4

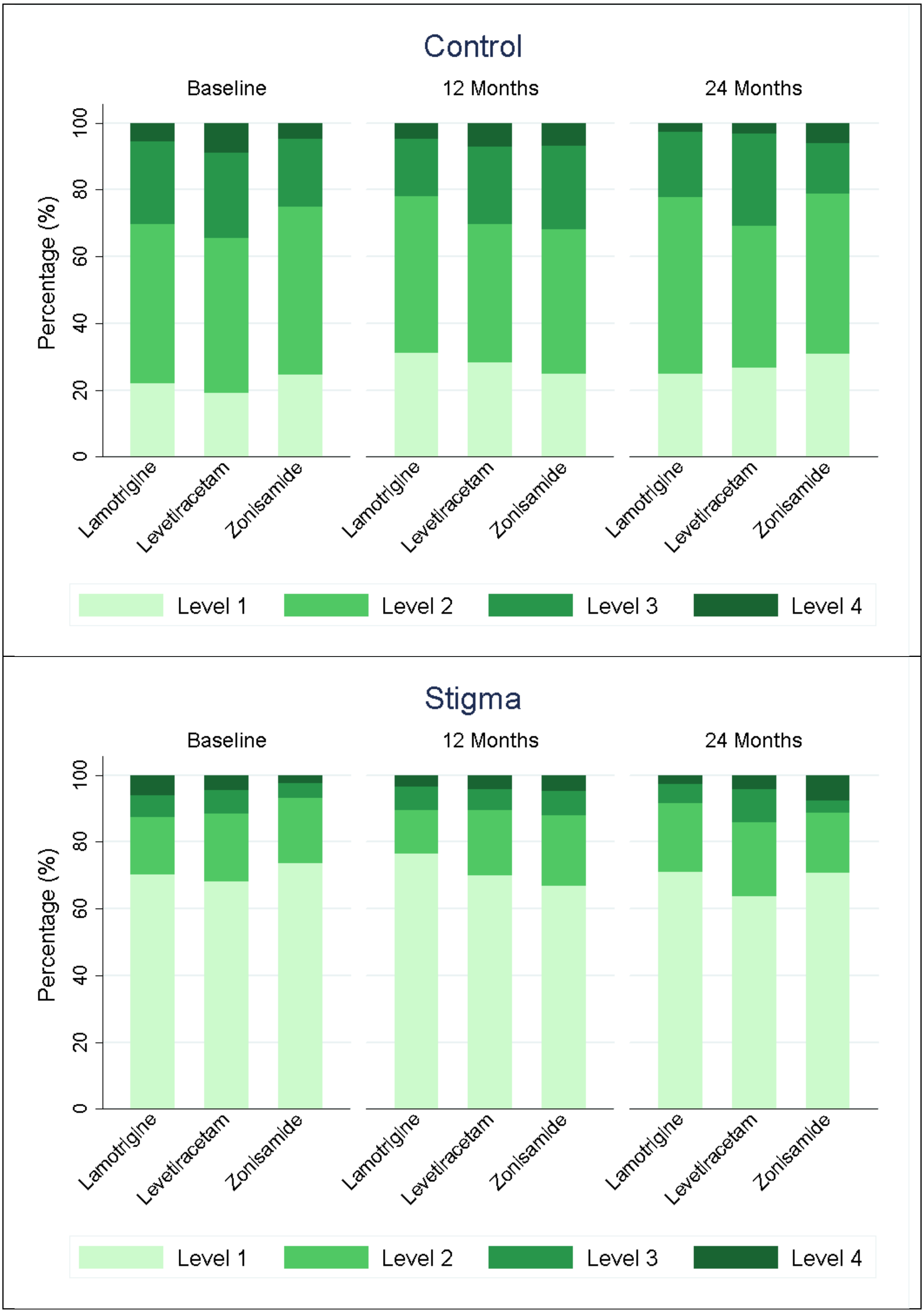
Control
Baseline
12 Months
24 Months
Percentage (%)
0
20
40
60
80
100
Lamotrigine
Levetiracetam
Zonisamide
Level 1
Level 2
Level 3
Level 4
Stigma

Table 8. Responses to the EQ-VAS thermometer, by version and intervention group.

	Lamotrigine		Levetiracetam		Zonisamide	
	n	Mean (97.5 CI)	n	Mean (97.5 CI)	n	Mean (97.5 CI)
Baseline	188	0.712 (0.681, 0.744)	187	0.707 (0.672, 0.743)	190	0.751 (0.717, 0.784)
12 months	127	0.767 (0.722, 0.812)	124	0.706 (0.656, 0.757)	130	0.712 (0.664, 0.759)
24 months	106	0.752 (0.701, 0.803)	106	0.715 (0.656, 0.774)	109	0.726 (0.673, 0.780)

Incremental analysis

Based on the point estimate mean costs and QALYs, both levetiracetam and zonisamide were more costly and less effective than lamotrigine, and were therefore dominated, meaning that they are not considered to be cost-effective. Zonisamide is associated with a negative incremental net health benefit of -0.171 (97.5% CR -0.295, -0.055) compared with lamotrigine, whilst levetiracetam is associated with a negative health benefit compared with zonisamide -0.010 (97.5% CR -0.142, 0.112) at a cost-effectiveness threshold of £20,000 per QALY.

Sensitivity analyses

Table 9 presents the results of the sensitivity analyses, which are consistent with the base-case for all analyses other than the NEWQOL-6D, where the net health benefit for levetiracetam is higher than for zonisamide at the £20,000 per QALY cost-effectiveness threshold, and the complete case analysis where levetiracetam is associated with lower costs than lamotrigine, though lamotrigine is still associated with the higher net health benefit.

The cost-effectiveness acceptability curve (Figure 3) indicates that the probability of levetiracetam being the most cost-effective at a cost-effectiveness threshold of £20,000 per QALY, is 0, whilst the probability for zonisamide is 0.001.

Table 9: Results of sensitivity analyses. Anti-seizure medications ranked by cost-effectiveness, based on net health benefit at a cost-effectiveness threshold of £20,000 per QALY. Unless stated, incremental values are versus the row above.

	Mean (97.5% CR)					
	Total cost (£)	QALYs	NHB at £20,000 per QALY	NHB at £30,000 per QALY	INHB £20,000 per QALY	INHB £30,000 per QALY
Base case (n=990)						
LTG	4042 (3626, 4983)	1.605 (1.547, 1.651)	1.403 (1.319, 1.458)	1.470 (1.399, 1.520)		
ZON	5400 (4659, 6770)	1.502 (1.418, 1.566)	1.232 (1.112, 1.307)	1.322 (1.215, 1.392)	-0.171 (-0.295, -0.055)	-0.148 (-0.261, -0.045)
LEV	5104 (4450, 6141)	1.474 (1.393, 1.523)	1.222 (1.110, 1.283)	1.307 (1.204, 1.361)	-0.010 (-0.142, 0.112)	-0.015 (-0.136, 0.089)
0% Discount rate (costs and QALYs) (base case 3.5%) (n=990)						
LTG	4108 (3682, 5059)	1.633 (1.573, 1.680)	1.428 (1.343, 1.484)	1.496 (1.423, 1.546)		
ZON	5483 (4727, 6872)	1.528 (1.442, 1.592)	1.254 (1.131, 1.330)	1.322 (1.236, 1.416)	-0.174 (-0.300, -0.056)	-0.151 (-0.266, -0.045)
LEV	5189 (4517, 6255)	1.502 (1.417, 1.549)	1.243 (1.128, 1.305)	1.307 (1.224, 1.385)	-0.011 (-0.146, 0.114)	-0.016 (-0.139, 0.091)
6% Discount rate (costs and QALYs) (base case 3.5%) (n=990)						
LTG	3998 (3587, 4935)	1.586 (1.529, 1.632)	1.386 (1.303, 1.440)	1.453 (1.382, 1.501)		
ZON	5344 (4613, 6698)	1.485 (1.402, 1.548)	1.218 (1.100, 1.291)	1.307 (1.201, 1.376)	-0.168 (-0.291, -0.055)	-0.146 (-0.258, -0.044)
LEV	5046 (4405, 6066)	1.461 (1.378, 1.505)	1.208 (1.097, 1.268)	1.292 (1.191, 1.346)	-0.010 (-0.139, 0.111)	-0.014 (-0.133, 0.089)
Unadjusted (base case adjusted) (n=990)						
LTG	4063 (3617, 4842)	1.600 (1.524, 1.649)	1.397 (1.301,1.450)	1.465 (1.374, 1.515)		
ZON	5409 (4584, 6658)	1.521 (1.431, 1.591)	1.251 (1.078, 1.278)	1.341 (1.176, 1.354)	-0.146 (-0.279, -0.006)	-0.124 (-0.247, 0.005)

LEV	5074<br>(4433, 6049)	1.459<br>(1.362, 1.517)	1.205<br>(1.129, 1.339)	1.290<br>(1.233, 1.421)	-0.045<br>(-0.195, 0.095)	-0.051<br>(-0.183, 0.076)
Complete case data (cost n = 178; EQ-5D n=225) (base case imputed)						
LTG	3635<br>(2431, 4828)	1.628<br>(1.576, 1.684)	1.446<br>(1.367, 1.537)	1.507<br>(1.440, 1.583)		
LEV	3294<br>(2063, 4504)	1.481<br>(1.418, 1.545)	1. 316<br>(1.234, 1.401)	1.371<br>(1.299, 1.444)	-0.131 (-0.244, -<br>0.024)	-0.136 (-0.233, -<br>0.045)
ZON	4704<br>(3375, 6255)	1.548<br>(1.483, 1.601)	1.313<br>(1.200, 1.405)	1.391<br>(1.296, 1.466)	-0.003 (-0.094, 0.109)	0.020 (-0.094, 0.109)
Per protocol (n=959) (base case all participants, intention to treat)						
LTG	4052<br>(3626, 5023)	1.605<br>(1.546, 1.650)	1.402<br>(1.315, 1.456)	1.470<br>(1.397, 1.519)		
ZON	5118<br>(4702, 6826)	1.503<br>(1.420, 1.565)	1.229<br>(1.114, 1.304)	1.320<br>(1.217, 1.390)	-0.174<br>(-0.294, -0.059)	-0.150<br>(-0.255, -0.046)
LEV	5480<br>(4465, 6185)	1.478<br>(1.394, 1.523)	1.221<br>(1.401, 1.280)	1.307<br>(1.202, 1.361)	-0.007<br>(-0.137, 0.111)	-0.013<br>(-0.131, 0.088)
NEWQOL-6D (base case EQ-5D) (costs as base case, NEWQOL-6D based on n = 132 complete cases)						
LTG	4042<br>(3626, 4983)	1.710<br>(1.687, 1.733)	1.508<br>(1.455, 1.567)	1.575<br>(1.536, 1.600)		
LEV	5104<br>(4450, 6141)	1.703<br>(1.678, 1.727)	1.448<br>(1.390, 1.488)	1.533<br>(1.489, 1.565)	-0.060<br>(-0.119, -0.004)	-0.042<br>(-0.086, -0.000)
ZON	5400<br>(4659, 6770)	1.712<br>(1.690, 1.735)	1.442<br>(1.368, 1.483)	1.532<br>(1.479, 1.564)	-0.006<br>(-0.081, 0.060)	-0.001<br>(-0.054, 0.045)
EQ-VAS (base case EQ-5D) (n=990)						
LTG	4042<br>(3626, 4983)	1.431<br>(1.360, 1.476)	1.229<br>(1.127, 1.281)	1.296<br>(1.207, 1.346)		
ZON	5400<br>(4659, 6770)	1.418<br>(1.351, 1.456)	1.148<br>(1.044, 1.200)	1.238<br>(1.148, 1.283)	-0.081<br>(-0.183, 0.016)	-0.005<br>(-0.147, 0.028)
LEV	5104<br>(4450, 6141)	1.398<br>(1.324, 1.479)	1.142<br>(1.042, 1.223)	1.227<br>(1.138, 1.308)	-0.150<br>(-0.102, 0.121)	-0.013<br>(-0.093, 0.105)
Treating blank responses in the questionnaire as missing rather than zero						
LTG	4059	1.605	1.402	1.470		

		(1.547, 1.651)				
ZON	5532	1.502<br>(1.418, 1.566)	1.226	1.318	-0.176	-0.152
LEV	5100	1.474<br>(1.393, 1.523)	1.222	1.307	-0.003	-0.010

Figure 3. Cost effectiveness acceptability curve. Dashed lines represent cost-effectiveness thresholds of £20,000 per QALY and £30,000 per QALY.

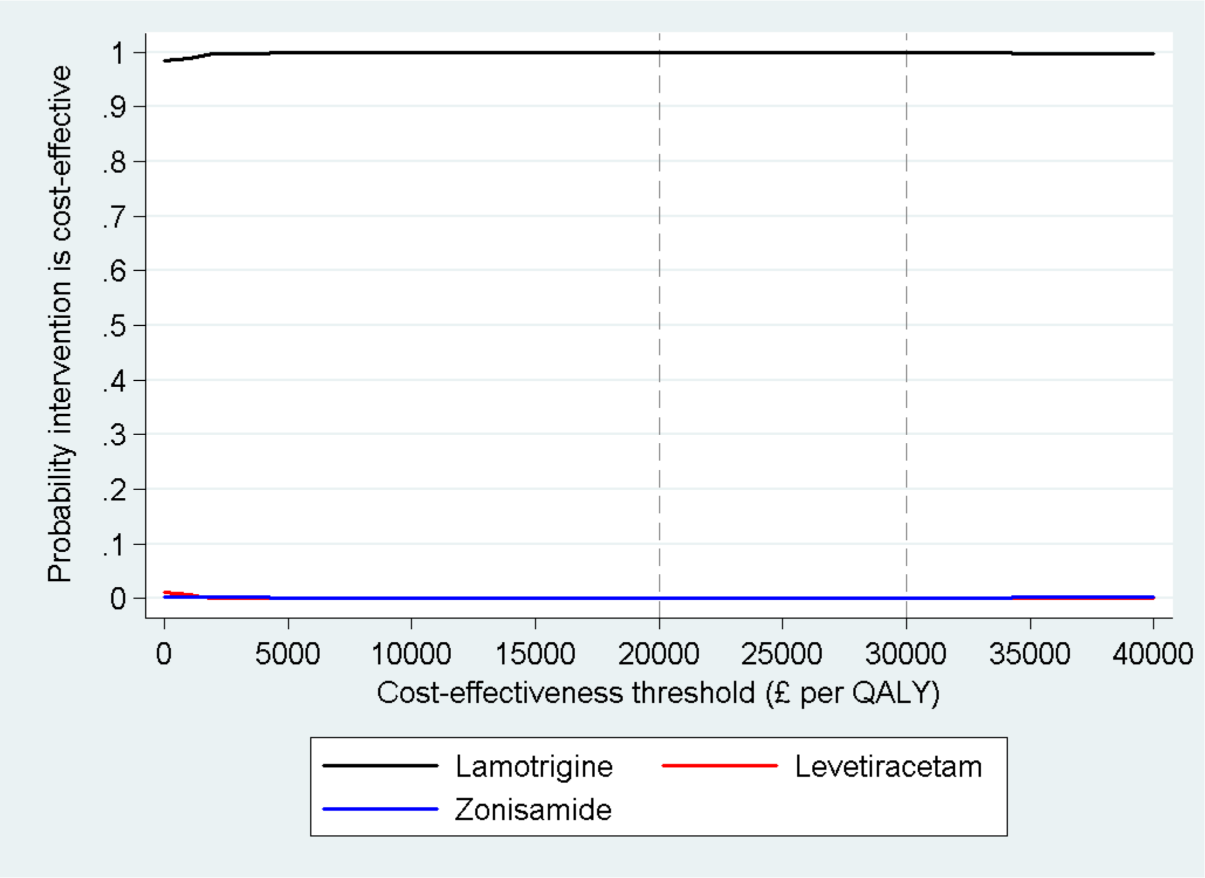

Sub-group analyses

The results of the subgroup analysis for adults, are consistent with the base-case analysis for the whole population (Table 10). For children, however, lamotrigine is associated with the highest costs £5076 (97.5% CR £3815, £7219), compared with levetiracetam £4972 (97.5% CR £3739, £6840), and zonisamide £4638 (97.5% CR £3826, £6974). Levetiracetam is associated with higher QALYs than lamotrigine, and therefore lamotrigine is dominated. Zonisamide has a lower cost, and lower QALYs than levetiracetam, but also a lower net health benefit at a cost-effectiveness threshold of £20,000 per QALY, and is therefore not cost effective at that threshold.

Table 10: Results of sub-group analysis. Anti-seizure medications ranked by cost-effectiveness, based on net health benefit at a cost-effectiveness threshold of £20,000 per QALY. Incremental values are versus the row above.

	**Mean (97.5% CR)**					
	Total cost (£)	QALYs	NHB at £20,000 per QALY	NHB at £30,000 per QALY	INHB £20,000 per QALY	INHB £30,000 per QALY
Base-case (n=990)						
LTG	4042 (3626, 4983)	1.605 (1.547, 1.651)	1.403 (1.319, 1.458)	1.470 (1.399, 1.520)		
ZON	5400 (4659, 6770)	1.502 (1.418, 1.566)	1.232 (1.112, 1.307)	1.322 (1.215, 1.392)	-0.171 (-0.295, -0.055)	-0.148 (-0.261, -0.045)
LEV	5104 (4450, 6141)	1.474 (1.393, 1.523)	1.222 (1.110, 1.283)	1.307 (1.204, 1.361)	-0.010 (-0.142, 0.112)	-0.015 (-0.136, 0.089)
Children aged under 16 years (n=155)						
LEV	4972 (3739, 6840)	1.556 (1.397, 1.618)	1.307 (1.097, 1.394)	1.390 (1.207, 1.463)		
LTG	5076 (3815, 7219)	1.551 (1.432, 1.638)	1.297 (1.107, 1.412)	1.382 (1.221, 1.481)	-0.010 (-0.171, 0.191)	-0.009 (-0.148, 0.173)
ZON	4638 (3826, 6974)	1.508 (1.381, 1.610)	1.277 (1.068, 1.390)	1.354 (1.176, 1.460)	-0.020 (-0.242, 0.175)	-0.028 (-0.214, 0.143)
Adults aged 16 years and over (n=835)						
LTG	3844 (3379, 4478)	1.612 (1.554, 1.661)	1.420 (1.346, 1.475)	1.484 (1.417, 1.536)		
ZON	5509 (4610, 6866)	1.508 (1.413, 1.569)	1.227 (1.101, 1.320)	1.319 (1.209, 1.398)	-0.193 (-0.322, -0.083)	-0.165 (-0.278, -0.067)
LEV	5178 (4435, 6223)	1.466 (1.381, 1.518)	1.207 (1.095, 1.280)	1.294 (1.193, 1.359)	-0.020 (-0.158, 0.112)	-0.025 (-0.149, 0.090)

*Less costly, less effective

References

1. Husereau D, Drummond M, Petrou S, et al. Consolidated Health Economic Evaluation Reporting Standards (CHEERS)--explanation and elaboration: a report of the ISPOR Health Economic Evaluation Publication Guidelines Good Reporting Practices Task Force. *Value Health* 2013; **16**(2): 231-50.
2. Beecham J KM. Costing psychiatric interventions. In: G T, ed. Measuring Mental Health Needs. 2nd ed. London: Gaskell; 2001: 200-24.
3. Marson AG, Appleton R, Baker GA, et al. A randomised controlled trial examining the longer-term outcomes of standard versus new antiepileptic drugs. The SANAD trial. *Health Technol Assess* 2007; **11**(37): iii-iv, ix-x, 1-134.
4. Database of Instruments for Resource Use Management. SANAD-II RUM https://www.dirum.org/instruments/details/93.
5. Balabanova S, Taylor C, Sills G, et al. Study protocol for a pragmatic randomised controlled trial comparing the effectiveness and cost-effectiveness of levetiracetam and zonisamide versus standard treatments for epilepsy: a comparison of standard and new antiepileptic drugs (SANAD-II). *BMJ Open* 2020; **10**(8): e040635.
6. National Institute for Health and Care Excellence. Guide to the Methods of Technology Appraisal 2013. Process and Methods [PMG9]. 2013.
7. NHS Digital Data Linkage & Extract Service. Available from: https://digital.nhs.uk/
8. The Secure Anonymised Information Linkage databank. Available from: https://saildatabank.com/.
9. Lomas J, Asaria M, Bojke L, Gale CP, Richardson G, Walker S. Which Costs Matter? Costs Included in Economic Evaluation and their Impact on Decision Uncertainty for Stable Coronary Artery Disease. *Pharmacoecon Open* 2018; **2**(4): 403-13.
10. Reference costs: https://improvement.nhs.uk/resources/national-cost-collection/ National Cost Collection: National Schedule of NHS costs - Year 2018-19 - NHS trust and NHS foundation trusts.
11. Curtis L BA. Unit Costs of Health and Social Care 2019. Unit Costs of Health and Social Care. PSSRU, Kent, UK, 176 pp. ISBN 978-1-911353-10-2.
12. Joint Formulary Committee. British National Formulary available from https://bnf.nice.org.uk/ [Accessed 17th Aug 2020].
13. NHS Business Services Authority. Prescription Cost Analysis (PCA) data September 2019. https://www.nhsbsa.nhs.uk/prescription-data/dispensing-data/prescription-cost-analysis-pca-data.
14. Welsh NHS Data dictionary 2020. http://www.datadictionary.wales.nhs.uk/#!WordDocuments/livedataitemsaz.htm.
15. NHS Digital. National Casemix Office HRG4+ 2018/19 Payment Grouper. 2019 https://digital.nhs.uk/services/national-casemix-office/downloads-groupers-and-tools/payment---hrg4-2018-19-local-payment-grouper
16. Curtis L BA. Unit Costs of Health and Social Care 2015, Personal Social Services Research Unit, University of Kent, Canterbury. 2015.
17. Department of Health and NHS England. Out-of-hours GP services in England. 2014. https://www.nao.org.uk/wp-content/uploads/2014/09/Out-of-hours-GP-services-in-England1.pdf [Accessed 17th Aug 2020].
18. National Health Service. NHS voucher values for glasses and lenses. https://www.nhs.uk/using-the-nhs/help-with-health-costs/nhs-voucher-values-for-glasses-and-lenses/ [Accessed 17th Aug 2020].
19. Gray E DA, Karssemeijer N, et al. . Evaluation of a Stratified National Breast Screening Program in the United Kingdom: An Early Model-Based Cost-Effectiveness Analysis. doi:10.1016/j.jval.2017.04.012. *Value Health* 2017; **20**(8): 1100-9. .

20. Bains I CY, Soldan K, Jit M. . Clinical impact and cost-effectiveness of primary cytology versus human papillomavirus testing for cervical cancer screening in England. 2019:ijgc-2018-000161. *Int J Gynecol Cancer* 2019.
21. Pope C TJ, Jones J, Pritchard J, Rowsell A, Halford S. . Has the NHS 111 urgent care telephone service been a success? Case study and secondary data analysis in England. *BMJ Open* 2017; **7(5): e014815**.
22. Kind P. The EuroQol Instrument: An Index of Health-Related Quality of Life. Quality of Life and Pharmacoeconomics in Clinical Trials, 2, 191-201.; 1996.
23. Mulhern B RD, Jacoby A, Marson T, Snape D, Hughes D, Latimer N, Baker GA, Brazier JE. . The development of a QALY measure for epilepsy: NEWQOL-6D. . *Epilepsy Behav* 2012; **24**(1): 36-43.
24. Dolan P. Modeling valuations for EuroQol health states. *Med Care* 1997; **35**(11): 1095-108.
25. Gabrio A, Mason AJ, Baio G. Handling Missing Data in Within-Trial Cost-Effectiveness Analysis: A Review with Future Recommendations. *Pharmacoecon Open* 2017; **1**(2): 79-97.
26. White I RP, Wood A. . Multiple imputation using chained equations: Issues and guidance for practice. *Stat Med* 2011; **30**(4): 377-99.
27. Graham JW OA, Gilreath TD. . How many imputations are really needed? Some practical clarifications of multiple imputation theory. *Prev Sci* 2007; **8**: 206-13.
28. van Asselt AD vMG, Dirksen CD, Arntz A, Severens JL, Kessels AG. . How to deal with cost differences at baseline. *Pharmacoeconomics* 2009; **27**(6): 519-28.
29. Mihaylova B BA, O'Hagan A, Thompson SG. . Review of statistical methods for analysing healthcare resources and costs. *Health Econ* 2011; **20**(8): 897-916.
30. Paulden M. Calculating and Interpreting ICERs and Net Benefit. *PharmacoEconomics* 2020; **38**: 785-807.
31. Fenwick E CK, Sculpher M. . Representing uncertainty: the role of cost-effectiveness acceptability curves. *Health economics* 2001; **10**: 779-87.
